# Investigation of anti-diabetic effect of a novel coenzyme Q10 derivative

**DOI:** 10.3389/fchem.2023.1280999

**Published:** 2023-10-19

**Authors:** Xiaojun Tan, Xinyi Yang, Xun Xu, Yuwei Peng, Xin Li, Yongxing Deng, Xueyang Zhang, Wenlong Qiu, Dudu Wu, Yongdui Ruan, Chen Zhi

**Affiliations:** ^1^ School of Pharmacy, Guangdong Medical University, Dongguan, China; ^2^ The First Dongguan Affiliated Hospital of Guangdong Medical University, Dongguan, China

**Keywords:** novel, CoQ10 derivative, insulin resistance, HepG2 cell, oxidative stress, HFD/STZ mice

## Abstract

**Introduction:** The rising incidence of type 2 diabetes has seriously affected international public health. The search for more drugs that can effectively treat diabetes has become a cutting-edge trend in research. Coenzyme Q10 (CoQ10) has attracted much attention in the last decade due to its wide range of biological activities. Many researchers have explored the clinical effects of CoQ10 in patients with type 2 diabetes. However, CoQ10 has low bio-availability due to its high lipophilicity. Therefore, we have structurally optimized CoQ10 in an attempt to exploit the potential of its pharmacological activity.

**Methods:** A novel coenzyme Q10 derivative (L-50) was designed and synthesized by introducing a group containing bromine atom and hydroxyl at the terminal of coenzyme Q10 (CoQ10), and the antidiabetic effect of L-50 was investigated by cellular assays and animal experiments.

**Results:** Cytotoxicity results showed that L-50 was comparatively low toxicity to HepG2 cells. Hypoglycemic assays indicated that L-50 could increase glucose uptake in IR-HepG2 cells, with significantly enhanced hypoglycemic capacity compared to the CoQ10. In addition, L-50 improved cellular utilization of glucose through reduction of reactive oxygen species (ROS) accumulated in insulin-resistant HepG2 cells (IR-HepG2) and regulation of JNK/AKT/GSK3β signaling pathway, resulting in hypoglycemic effects. Furthermore, the animal experiments demonstrated that L-50 could restore the body weight of HFD/STZ mice. Notably, the findings suggested that L-50 could improve glycemic and lipid metabolism in HFD/STZ mice. Moreover, L-50 could increase fasting insulin levels (FINS) in HFD/STZ mice, leading to a decrease in fasting blood glucose (FBG) and hepatic glycogen. Furthermore, L-50 could recover triglycerides (TG), total cholesterol (T-CHO), lipoprotein (LDL-C) and high-density lipoprotein (HDL-C) levels in HFD/STZ mice.

**Discussion:** The addition of a bromine atom and a hydroxyl group to CoQ10 could enhance its anti-diabetic activity. It is anticipated that L-50 could be a promising new agent for T2DM.

## 1 Introduction

Worldwide, the majority of the 537 million people with diabetes are type 2 diabetes mellitus (T2DM) (Ahmad et al., 2022). T2DM is a chronic disease causing various complications that seriously affect the normal function of the physical organism (Vijan, 2019). It is thought that insulin resistance (IR) (Rachdaoui, 2020) and oxidative stress (Rehman and Akash, 2017) are intimately involved in the pathogenesis of T2DM. Currently, the main antidiabetic drugs include metformin, thiazolidinediones and sulfonylureas (BennettMaruthurSingh et al., 2011). However, these hypoglycemic agents present different clinical hazards, such as undesirable cardiovascular reactions (Leonard et al., 2020) and birth defects in men (Louis, 2022). Therefore, the search and development of new anti-hyperglycemic agents is of utmost importance.

CoQ10 is a natural antioxidant found mainly in highly metabolic organs of the liver, kidneys and heart (Martelli et al., 2020). Considerable research efforts have confirmed the important role of CoQ10 in aging (López-Lluch, 2019), cardiac oxidative damage (Liang et al., 2017) and diabetes nephropathy (Couto et al., 2021). Recently, it was reported that CoQ10 was involved in the treatment of diabetes through oxidative stress damage in the organism and prevention of impaired insulin signaling (Nakazawa et al., 2019; Banks and Rhea, 2021). For example, Moazen et al. found that coenzyme Q10 supplementation could decrease the levels of glycated hemoglobin and malondialdehyde (a marker of oxidative stress) significantly in patients with T2DM (Moazen et al., 2015). Fallah et al. demonstrated that CoQ10 supplementation facilitated a decrease in serum levels of insulin, triglycerides and cholesterol in diabetic patients (Fallah et al., 2018). Raygan et al. reported that CoQ10 was helpful in assessing insulin resistance homeostasis model and homeostasis model assessment-β cell function in diabetic patients (Raygan et al., 2016). These researches confirmed the capability of CoQ10 to slow down the progression of diabetes (Yoo and Yum, 2018) and to alleviate the complication symptoms in patients with T2DM (Tabatabaei-Malazy et al., 2019; Amini et al., 2022).

However, the bioavailability of exogenous CoQ10 is quite minimal since it exhibits high lipophilicity, resulting in poor solubility dispersion in the digestive tract (Mantle and Dybring, 2020; Bhalani et al., 2022). Interestingly, the introduction of hydroxyl groups could increase the polarity and hydro-solubility of the drug (Cramer et al., 2019). For instance, Akio et al. obtained new derivatives by introducing tetraethylene glycol side chains on 2-phenylthioalkylhydroquinone dimers, which showed sufficient water solubility (Kamimura et al., 2021).In addition, the introduction of halogen atoms is a traditional approach to drug development (Mendez et al., 2017), as reflected in two aspects: the ability of halogenation to enhance drug binding and penetration into cell membranes (Gerebtzoff et al., 2004) and the ability of halogen bonding to improve the affinity of drugs to their targets (Xu et al., 2014). Yeliz et al. found that bromophenol structure could exert antidiabetic potential by inhibiting aldose reductase (Demir et al., 2018); Pradeep et al. found that bromophenol extracted from seaweeds was an inhibitor of PTP1B and α-glucosidase, and enhanced insulin sensitivity and glucose uptake (Paudel et al., 2019); Necla et al. obtained a novel bromophenol by introducing three bromine atoms and found its antidiabetic properties by *in vitro* bioassay. Necla et al. obtained a new bromophenol by introducing three bromine atoms and found it to possess antioxidant and antidiabetic activities by *in vitro* bioassay (Öztaşkın et al., 2019).

Hence, we propose the hypothesis that the combination of bromine atoms with hydroxyl groups could allow drugs to exhibit greater potential for enhancing original biological activities or developing new ones. We performed an electrophilic addition reaction at the end of the side chain of CoQ10 to introduce a bromine atom and a hydroxyl group thereby exploiting the pharmacological potential of CoQ10 derivatives. Then, the *in vitro* toxic activity of L-50 was investigated by cellular experiments, and the anti-diabetic mechanism of L-50 was analyzed by the ROS and protein signaling pathways. Finally, the effects of L-50 on body weight as well as glucose-lipid metabolism indexes in HFD/STZ mice were also investigated.


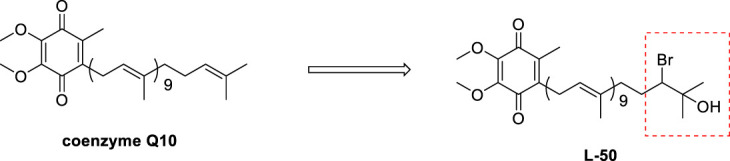


## 2 Materials and methods

### 2.1 Chemistry

#### 2.1.1 General

The NMR spectra data of synthetic compounds was obtained by spectrometers (^1^H at 400 MHz and ^13^C at 100 MHz). Chemical shifts (δ) were given in ppm with reference to solvent signals [^1^H NMR: CDCl_3_ (δ 7.26 ppm); ^13^C NMR: CDCl_3_ (δ 77.0 ppm)].

#### 2.1.2 Synthesis of L-50

To a stirred solution of coenzyme Q10 (CoQ10) (80 mg, 0.09 mmol) in dry THF (3 mL) was slowly added water at 0 C until the mixture remained turbid. And THF was then added to the resulting mixture until the reaction just cleared. Then N-bromosuccinimide (NBS) (13 mg, 0.07 mmol) was added to the reaction mixture, which was stirred at room temperature for 4 h. Finally, the reaction was quenched with water (10 mL). The resulting mixture was extracted with EA (3 × 10 mL). The combined organic layers were washed with water (10 mL), brine (10 mL), dried over Na_2_SO_4_, filtered and concentrated *in vacuo*. The residue was purified by silica gel chromatography (petroleum ether/EtOAc, 5:1) to give compound L-50 (48 mg, 54%) as a yellow oil. R_f_ = 0.22 (petroleum ether/EtOAc, 9:1).

^1^H NMR (400 MHz, CDCl_3_) δ 5.20 (td, *J* = 6.1, 5.7, 3.1 Hz, 1H), 5.10 (q, *J* = 8.2, 7.6 Hz, 7H), 4.96–4.90 (m, 1H), 3.98 (d, *J* = 5.8 Hz, 7H), 3.18 (d, *J* = 7.0 Hz, 2H), 2.13 (d, *J* = 8.1 Hz, 1H), 2.11–2.02 (m, 18H), 2.01 (s, 3H), 2.00–1.92 (m, 17H), 1.73 (d, *J* = 1.3 Hz, 3H), 1.59 (d, *J* = 1.6 Hz, 21H), 1.57 (d, *J* = 1.2 Hz, 3H), 1.34 (s, 3H), 1.32 (s, 3H).^13^C NMR (100 MHz, CDCl_3_) δ 184.76, 183.90, 144.34, 144.19, 141.66, 138.85, 137.61, 135.23, 134.98, 134.92, 134.67, 132.93, 126.00, 124.43, 124.23, 124.13, 123.82, 118.82, 72.42, 70.99, 61.12, 39.72, 39.69, 39.59, 38.14, 32.12, 26.69, 26.61, 26.49, 25.72, 25.28, 16.32, 16.01, 15.82, 11.93.

HRMS (ESI): *m/z* calcd for C_59_H_91_BrNaO_5_ [M + Na]^+^: 981.5948, found:981.5952.

### 2.2 Cell culture

Human HepG2 cells were obtained from China-US Institute of Oncology, Guangdong Medical University, China. HepG2 cells were cultured at 37°C, 5% CO_2_, in high glucose medium (DMEM, GIBCO, USA) containing fetal bovine serum (FBS, GIBCO, USA). In each experiment, the cells in each group were repeated three times in parallel experiments. In the following experiments, we referred to the cells in the control group (normal cells) as HepG2 cells, and the cells in the model group (which were insulin-induced to develop insulin resistance) as IR-HepG2 cells. We considered rosiglitazone as a positive control drug, which belonged to the thiazolidinediones class of antidiabetic drugs for the treatment of type 2 diabetes mellitus by effectively controlling blood glucose by increasing the sensitivity of target tissues to insulin (Derosa et al., 2009).

### 2.3 Cell viability assay

Cell viability was determined to avoid inaccuracies in experimental results due to the effects of drugs on the physical and metabolic state of the cells. Also, this could be used as a cellular level drug toxicity assessment. The effect of different concentrations of L-50 on the viability of HepG2 cells was examined by CCK8 (China Tong Ren Chemical Co.). HepG2 cells were inoculated into 96-well plates at a density of 5.0 × 10^3^ cells/well and cultured in DMEM for 24 h. After that, the medium was changed and the cells were treated with rosiglitazone (Rosi), Coenzyme Q10 (CoQ10) and L-50 (1, 10, 20, 50, 100 μM) for 24 h. After treatment, 10 μL of CCK8 was added to each well, incubated at 37°C for 1 h, and OD was measured at 450 nm with a microplate reader.

### 2.4 Cellular glucose consumption measurement test

HepG2 cells in logarithmic growth phase were inoculated into 96-well plates (5.0 × 10^3^ cells/well) and divided into blank group (no cells), control group (without insulin), model group (insulin pretreatment for 24 h), Rosi group (insulin pretreatment for 24 h followed by addition of 20 μM rosiglitazone), CoQ10 group (insulin pretreatment for 24 h followed by addition of 20 μM CoQ10), as well as treatment group (insulin pretreatment for 24 h followed by addition of different concentrations of L-50). The glucose consumption of HepG2 cells in the medium is measured using the Glucose Assay Kit (China Nanjing Construction Co.). A 96-well plate was prepared by adding the prepared working solution, taking cell supernatant or calibration solution and mixing it with the working solution. It was incubated in a cell incubator for 10 min and the absorbance was measured by an enzyme meter. The method used in the kit was the glucose oxidase-peroxidase assay. The principle of the assay was as follows: glucose contained in the cell supernatant was generated by glucose oxidase to gluconic acid and hydrogen peroxide, the latter of which, in the presence of peroxidase, condenses reduced 4-aminoantipyrine coupled with phenol to quinone compounds that can be measured. The supernatant glucose content could be obtained by formula calculation. The consumption of glucose by the cells was equal to the initial glucose content (the results of the blank group) minus the remaining glucose content (the results of the other groups).

### 2.5 Lactate concentration measurement

HepG2 cells in logarithmic growth phase were inoculated into 96-well plates (1.0 × 10^4^ cells/well), with the control group, the model group, the Rosi group, the CoQ10 group and the treatment group. Lactic acid content of cell supernatants was measured using Lactate Assay Kit-WST (Tong Ren Chemical, Japan). The cell supernatant and calibration solution were added to the 96-well plates, and then the prepared working solution was added and mixed well. The plates were incubated in a cell culture incubator for 30 min, and then the absorbance was measured with an enzyme meter. Finally, the lactate concentration of the cell supernatant was calculated using a standard curve.

### 2.6 Measurement of ROS production

Reactive oxygen species (ROS) was measured to determine the relationship between insulin resistance and oxidative stress. It could also be used as one of the bases for drugs to improve insulin resistance. Intracellular ROS levels could be detected by laser confocal microscopy. HepG2 cells were inoculated in 6-well plates at 3.0 × 10^5^ cells/well and divided into control group (without insulin and L-50), model group (insulin pretreatment for 24 h), Rosi group (insulin pretreatment for 24 h followed by addition of 20 μM rosiglitazone), as well as treatment group (insulin pretreatment for 24 h followed by addition of different concentrations of L-50). After the modeling and drug administration treatments, 1.5 mL of diluted DCFH-DA (China Biyuntian Biotechnology Co.) was added to each group of cells sepamiceely. In this case, we prepared DCFH-DA at a final concentration of 10 µM. After incubation for 30 min at protection from light, the cells were replaced with serum-free medium and transferred to a laser confocal microscope under dark conditions for observation and photography.

### 2.7 Western blotting analysis

HepG2 cells were inoculated in 6-well plates at 1 × 10^6^ cells/well and divided into control group (no insulin and L-50), model group (insulin pretreatment for 24 h) and treatment group (insulin pretreatment for 24 h followed by different concentrations of L-50). Groups of cells were lysed with RIPA buffer and collected. Primary antibodies include JNK (51151-1-AP, Proteintech, China), phosphorylated JNK (p-JNK, 4668T, CellSignalingTechnology, USA), AKT (10176-2-AP, Proteintech, China), phosphorylated AKT (p-AKT, 66444-1-lg, Proteintech, China), GSK3β (22104-1-AP, Proteintech, China), β-actin (66009-1-Ig, Proteintech, China), and GAPDH (10494-1-AP, Proteintech, China) were used in this study. The dilutions of primary antibodies were as follows: 1:500 for JNK; 1:1000 for p-JNK, GSK3β and GAPDH; and 1:2000 for AKT, p-AKT and β-actin.Protein expression was measured with luminescent solutions, incubated with the relevant secondary antibodies, both of which were at a dilution of 1:2000. Then, the color was developed by a multifunctional molecular imaging system, and the results of relevant protein band densities were analyzed with ImageJ software.

### 2.8 HFD/STZ mice model and L-50 treatment

A total of 48 clean male KM mice (45 g ± 5 g) (Guangdong Animal Center) were used. They were acclimatized in an environment with a temperature of 26°C and 55% humidity for 3 days before the experiment. Mice were randomly divided into a normal group (8 mice, *ad libitum* feeding and drinking) and a model group (40 animals, *ad libitum* feeding and drinking). We established a diabetic mice model using high-fat diet (HFD) and streptozotocin (STZ). The STZ solution was prepared as follows: 2.10 g citric acid was dissolved in 100 mL of double-distilled water to form 0.1 mol/L citric acid master batch as solution A; 2.94 g sodium citrate was dissolved in 100 mL of double-distilled water to form 0.1 mol/L sodium citrate master batch as solution B. A:B was mixed in the ratio of 1:1.32 (v/v), and pH = 4.5 was adjusted to obtain sodium citrate buffer solution. 1 g STZ was dissolved in 100 mL of sodium citrate buffer solution to obtain a concentration of 10 mg/mL (1% STZ). Mice in the model group were fed with a high-fat diet (HFD) for 4 weeks, and 1% STZ injection (35 mg/kg/d) was administered intraperitoneally for 7 consecutive days at the third week. Tail vein blood collection was performed on days 3, 5 and 7 after the completion of STZ modeling to measure their random blood glucose, and the T2DM model was considered successful when all three times were higher than 16.7 mmol/L. After successful modeling, the model group mice were randomly divided into four groups (6 mice in each group): model group, positive group (Rosi, 200 mg/kg/d, administered by gavage for 2 weeks), CoQ10 group (200 mg/kg/d, administered by gavage for 2 weeks), low concentration L-50 group (LC, 200 mg/kg/d, administered by gavage for 2 weeks) and high concentration L-50 group (HC, 400 mg/kg/d, administered by gavage for 2 weeks).

The mice were weighed and recorded at the same time each week during the medication period. Mice were fasted for 12 h after the end of dosing and fasting blood glucose (FBG) was measured in tail vein blood using a glucometer. Blood samples were collected from mice, and serum was obtained by centrifugation to measure fasting insulin levels (FINS) as well as triglyceride (TG), total cholesterol (T-CHO), low-density lipoprotein (LDL-C), and high-density lipoprotein (HDL-C) levels in mice. Finally, the mice were euthanized and their liver tissues were taken to determine the content of liver glycogen in the liver tissues. Homeostatic Model Assessment for Insulin Resistance (HOMA-IR) = fasting glucose (mmol/L) × fasting insulin (mIU/L)/22.5.

Glucometer provided direct readings of glucose concentration in mouse blood. The GPO-PAP method for determining TG in mouse serum was based on the reaction of TG with lipase, glycerol kinase, glycerol-3-phosphate oxidase, and peroxidase to produce a red quinone compound. T-CHO in mouse serum was measured by the reaction of T-CHO with cholesterol esterase, cholesterol oxidase and peroxidase to form red quinone compounds. The amount of TG and T-CHO could be calculated by measuring the absorbance of the quinone compound. Determination of LDL-C and HDL-C in mouse serum was based on the principle that LDL-C and HDL-C were chemically modified by CHER and CHOD to produce benzoquinone pigments in the presence of peroxidase. The levels of LDL-C and HDL-C could be calculated by measuring benzoquinone pigments. The principle of liver glycogen determination was as follows: Glycogen was dehydrated under the condition of strong acid to form 5-hydroxymethylfurfural, and then reacted with anthrone to form blue-green and blue-green furfural derivatives, and the product appeared a characteristic absorption peak at 620 nm, and the change of absorbance was used to detect the content of glycogen quantitatively.

### 2.9 Statistical analysis

Data were expressed as mean ± SD. GraphPad Prism 9.0 software was applied for statistical analysis, using one-way ANOVA or two-way ANOVA followed by Tukey’s multiple comparisons test. In this study, *p* < 0.05 and *p* < 0.01 were considered significant.

## 3 Results and discussion

### 3.1 Synthesis of L-50


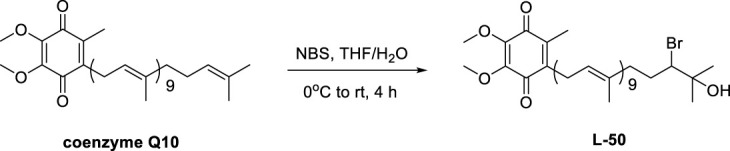


We envisaged that the introduction of a bromine atom and a hydroxyl group to the end of the side chain in CoQ10 would be realized by a chemselective electrophilic addition, Much to our delight, after a series of unsuccessful trials, we found that treatment of CoQ10 with N-bromosuccinimide (NBS) in the presence of THF/H_2_O successfully delivered the desired product L-50.

### 3.2 Cell cytotoxicity and glucose consumption of L-50

**FIGURE 1:**
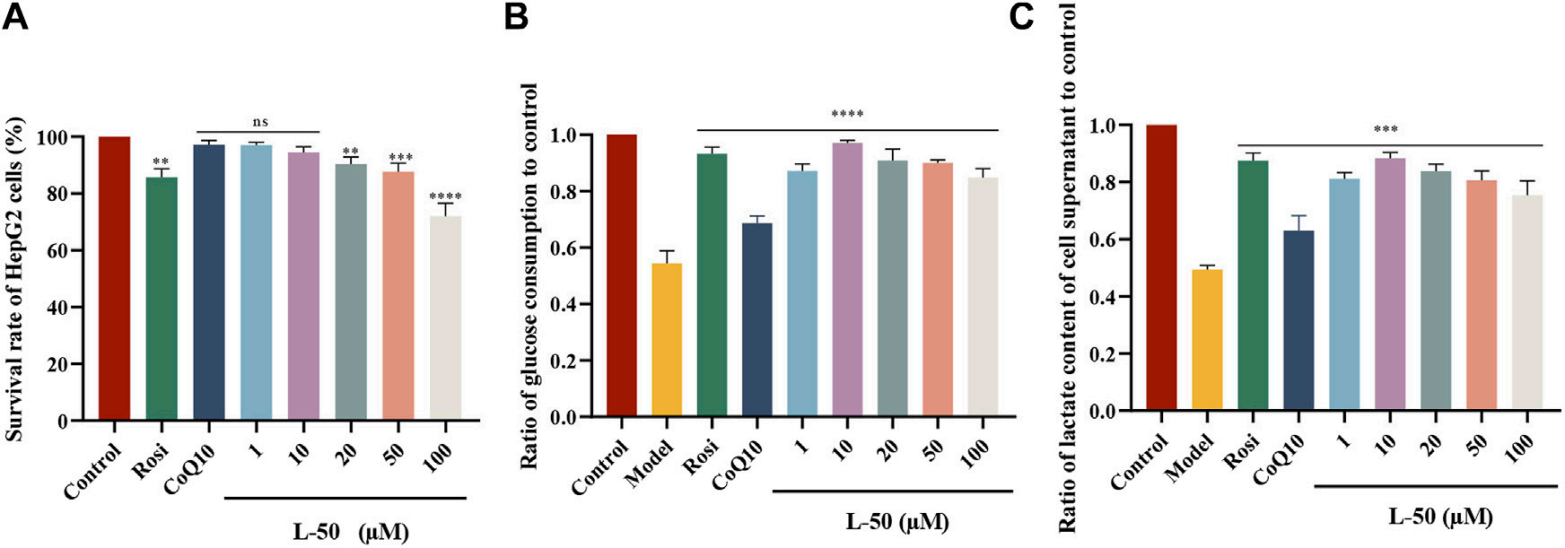
**(A)** L-50, CoQ10 and Rosi stimulated HepG2 cells for 24 h. CCK8 assay showed cell viability of HepG2, *n* = 6. ^ns^P>0.05, **p < 0.01, ***p < 0.001 and ****p < 0.0001 compared with the control group. **(B)** Treatment with L-50, CoQ10 and Rosi promoted glucose consumption in IR-HepG2 cells (Model, pretreated with insulin for 24 h). *n* = 6, ****p < 0.0001 compared with the model group. **(C)** Lactate concentration in the supernatant of each group of cells was assayed after treatment with L-50, CoQ10 and Rosi. *n* = 6, ****p < 0.0001 compared with the model group.

The cell cytoxicity of L-50, CoQ10 and Rosi on HepG2 cells (Figure 1A), and glucose consumption in insulin resistant HepG2 (IR-HepG2) are investigated (Figure 1B). As shown in Figure 1A, the viability of HepG2 cells in the L-50-treated group was decreased in a concentration-dependent manner. Compared with the control group, the survival rate of HepG2 cells was higher than 95% under the stimulation of L-50 below 10 μM, indicating that the low concentration of L-50 exhibited no significant cytotoxic effect. In addition, the viability of HepG2 cells under stimulation with L-50 below 10 μM was higher than that of the Rosi group, suggesting a good safety profile of the cells with L-50.

In a high-insulin environment, HepG2 cells become less sensitive to insulin and develop insulin resistance (IR). This results in diminished glucose uptake and utilization by HepG2 cells, manifested by a significant decrease in glucose consumption (Xuguang et al., 2019). As shown in Figure 1B, the glucose consumption of IR-HepG2 cells in the model group was significantly reduced compared with that of HepG2 cells in the control group. This effect of inducing HepG2 cells to become IR-HepG2 cells in agreement with the study of Fan et al. (2019). After the intervention of drugs (L-50 and CoQ10), glucose consumption was substantially increased compared with model group, showing that L-50 and CoQ10 had effects on hypoglycemia *in vitro.* Increased glucose consumption implied an elevated cellular uptake and utilization of glucose, suggesting a positive effect of the drug on insulin resistance (IR), which was accordance with the findings of Zheng et al. (Zheng et al., 2016). In addition, the glucose consumption in the L-50 group was significantly higher than that in the CoQ10 group and close to the Rosi group. These results suggest that L-50 could not only enhance the ability of cells to absorb and utilize glucose, but also alleviate IR better than CoQ10.

### 3.3 Determination of lactate in cell supernatants

Many studies had confirmed that cells utilize glucose mainly through glycolysis, aerobic oxidation and glycogen synthesis, and lactate was one of the metabolic products of glycolysis (Sun et al., 2023). In our lactate content determination experiments, the cells were cultured under anaerobic conditions. Therefore, we considered that cells consumed glucose mainly through glycolysis after glucose intake. As shown in Figure 1C, the lactate concentration was significantly lower in the model group compared to the control group, indicating the inhibition of cellular glycolysis. Compared with the model group, the lactate concentration in the L-50 group was significantly higher, showing that L-50 could promote the glycolysis process of the cells. These results validated our thoughts and verified each other with the results of Figure 1C.

### 3.4 Effect of L-50 on ROS level

**FIGURE 2:**
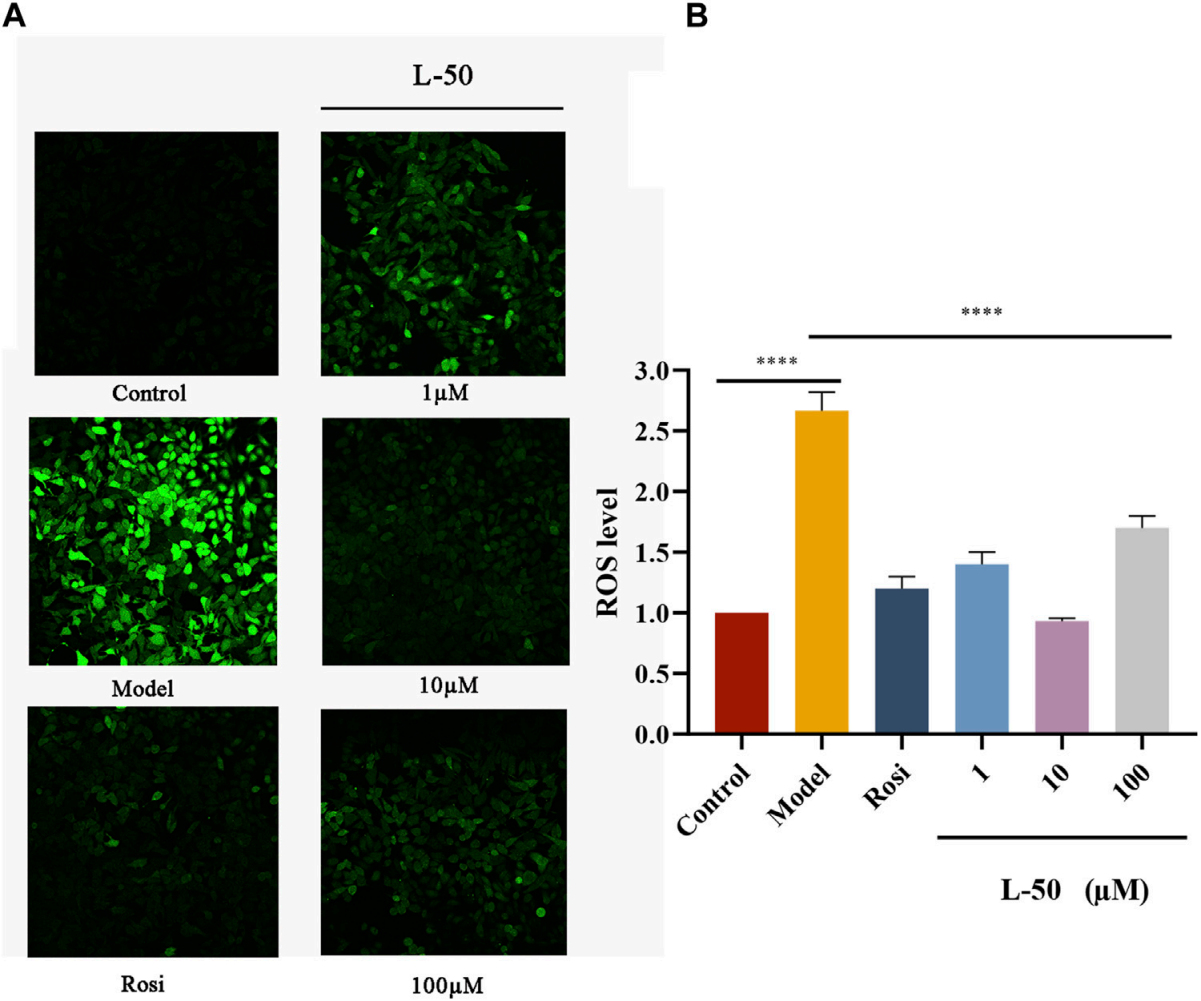
**(A)** Representative photographs of IR-HepG2 cells (Model, pretreated with insulin for 24 h) treated with Rosi and L-50 for 24 h and stained with H2DCFDA followed by laser confocal detection of ROS are shown. **(B)** Histogram of the effect of Rosi and L-50 on intracellular ROS content in IR-HepG2 cells. *n* = 3, ****p < 0.0001 compared with the model group.

Figure 2 showed the effect of L-50 and Rosi on ROS in IR-HepG2 cells. As compared to the control group, the cells in the model group were in a state of high-glucose due to its difficulty in consuming glucose. Many studies had confirmed that high-glucose could induce cells to produce excessive ROS (Luo et al., 2018; Park et al., 2023). Excessive ROS could cause oxidative stress and thus induce insulin resistance in HepG2 cells (Newsholme et al., 2016), which was a critical factor in the development of T2DM (Nascè et al., 2022). As seen in Figure 2, the ROS fluorescence was apparently brighter in the model group compared to the control group, confirming that excess ROS is a contributing factor to IR. This is consistent with the mechanism discussed above. The ROS fluorescence brightness of IR-HepG2 cells was significantly reduced after treatment with L-50, suggesting that intracellular ROS deposition can be reduced after drug administration.

In addition, the fluorescence intensity of intracellular ROS was the weakest after 10 μM L-50 treatment, indicating that L-50 was the most effective in reducing the accumulation of intracellular ROS, which is concordance with the results of previous studies on cellular activity. Thus, these results suggested that L-50 could alleviate IR by reducing the accumulation of ROS.

### 3.5 Effect of L-50 on JNK/AKT/GSK3β signaling pathway

**FIGURE 3:**
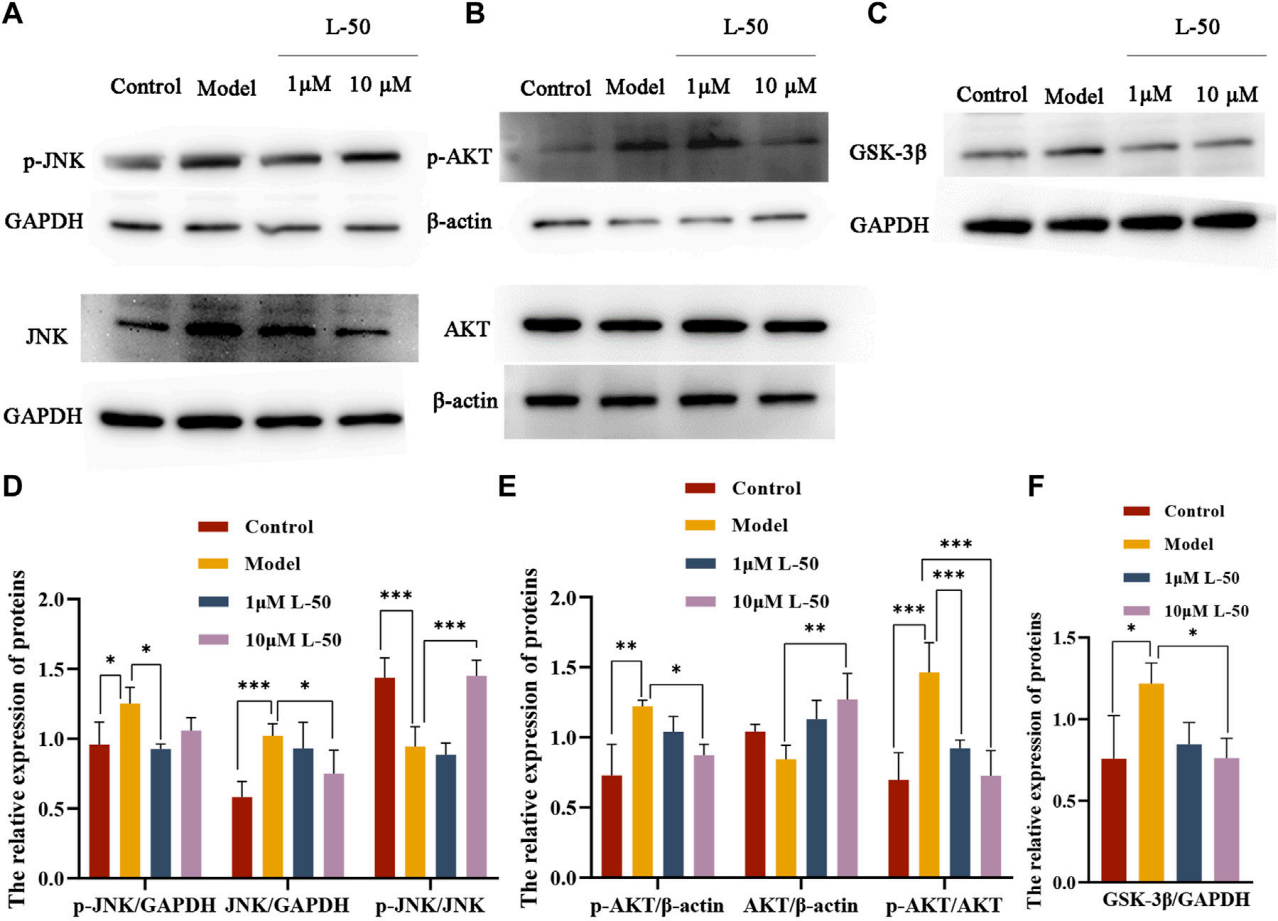
**(A–C)** Western blot showed JNK, p-JNK, AKT, p-AKT, GSK-3β protein bands after treatment of IR-HepG2 cells (Model, pretreated with insulin for 24 h) with L-50. **(D–F)** Image J analysis of the relative expression levels of the proteins. n = 3, *p < 0.05, **p < 0.01and ***p < 0.001 compared with the model group.

Many studies identified that exorbitant ROS could disrupt insulin signaling by activating the serine/threonine kinase c-Jun NH-terminal kinase (JNK), and that phosphorylation of JNK (p-JNK) could stimulate exacerbation of insulin resistance (Mo et al., 2019; Feng et al., 2020). It was noted that JNK was involved in the regulation of the insulin signaling pathway as a “promoter” along with AKT (a downstream molecule of the phosphatidylinositol 3-kinase signaling pathway) (Liu et al., 2017; Luo et al., 2019). As shown in Figures 3A, D, the expression of p-JNK and JNK proteins was increased in IR-HepG2 cells compared with the control group, while both expressions were decreased after L-50 treatment. These changes affected the activation and phosphorylation of the downstream molecule AKT. As seen in Figures 3B, E, in IR-HepG2 cells, AKT expression was decreased and p-AKT expression was increased, while we found that AKT expression was upregulated and p-AKT expression was downregulated after L-50 treatment. These results revealed that L-50 could inhibit the expression of JNK and p-JNK, improve insulin sensitivity, and enable correct insulin signaling to the downstream molecule AKT, which was in keeping with the results of changes in ROS content detected by laser confocal assay.

Furthermore, it was demonstrated that GSK3β was a downstream substrate of insulin and Akt signaling, which played an important function in maintaining glucose homeostasis by negatively regulating glycogen synthesis in the insulin signaling pathway (Yang et al., 2018; Xia et al., 2022). As shown in Figures 3C, F, GSK3β expression was increased in IR-HepG2 cells compared with the control group, and GSK3β expression was downregulated after L-50 treatment, suggesting that L-50 could decrease intracellular glucose concentration and increase glucose uptake by regulating intracellular glycogen synthesis, which was validated with the previous cellular activity results. These results suggested that the mechanism of action of L-50 in reversing insulin resistance in HepG2 cells might be related to the JNK/AKT/GSK3β signaling pathway.

### 3.6 Changes in bodyweight of HFD/STZ mice during medication

**FIGURE 4:**
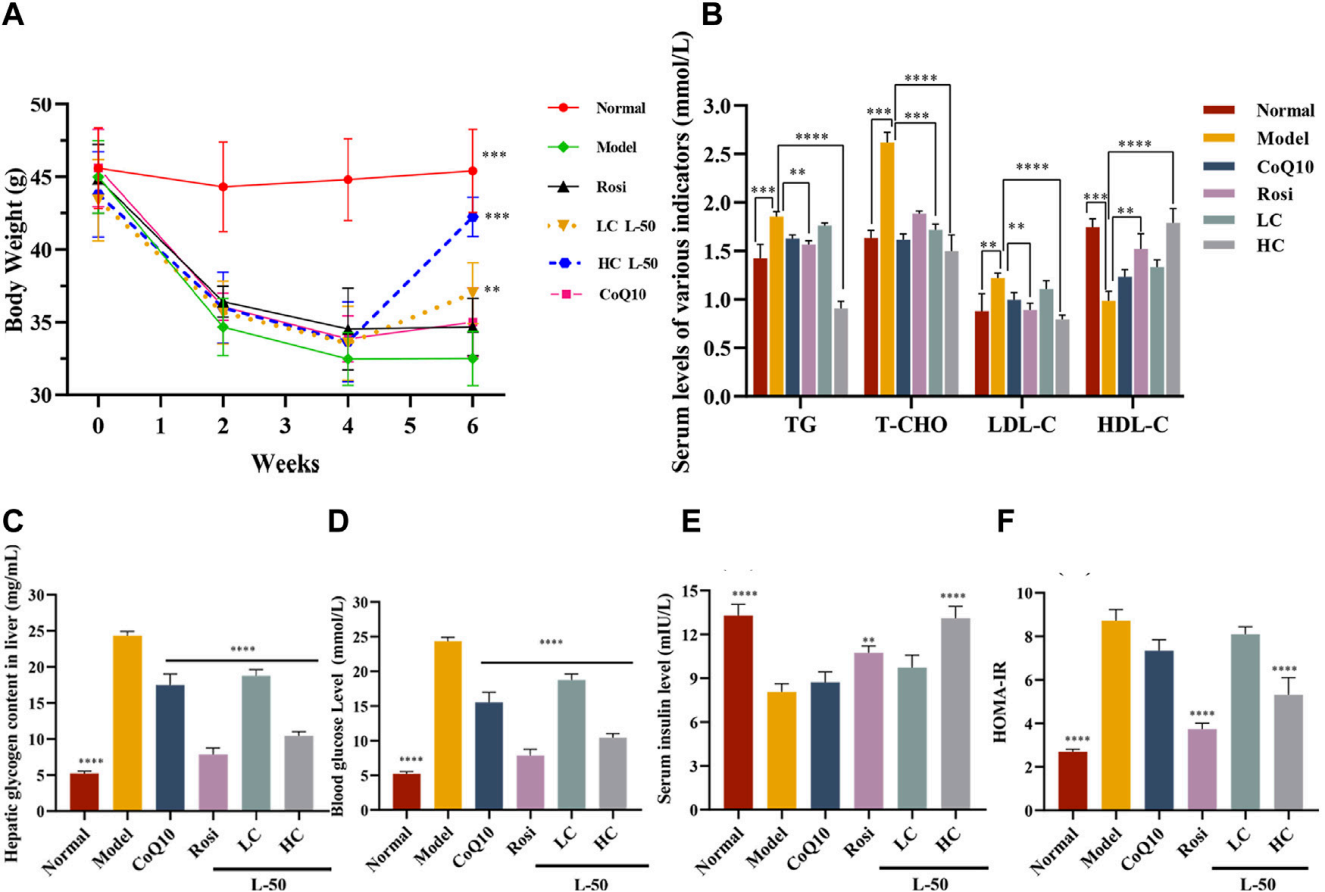
**(A)** Body weight changes in mice during modeling (HFD/STZ, 0–4 weeks) and medicine delivery (Rosi, CoQ10, L-50, 4–6 weeks). **(B)** Effects of Rosi, CoQ10 and L-50 on serum levels of triglycerides (TG), total cholesterol (T-CHO), low-density lipoprotein (LDL-C) and high-density lipoprotein (HDL-C) in HFD/STZ mice. **(C)** Effects of Rosi and L-50 on hepatic glycogen content in the liver of HFD/STZ mice. **(D–F)** Effects of Rosi, CoQ10 and L-50 on HFD/STZ mice fasting blood glucose (FBG), fasting insulin (FINS) and HOMA-IR levels in serum. *n* = 8,**p < 0.01, ***p < 0.001 and ****p < 0.0001 compared to model group.

Generally speaking, T2DM patients suffered from insulin deficiency and the body was unable to utilize glucose fully, which was reflected in weight loss (Ramos et al., 2021). Therefore, weight control is one of the indicators to determine the efficacy of diabetes mellitus. As shown in Figure 4A, the body weight of HFD/STZ mice was decreased after successful establishment of the animal model within 0–4 weeks. It agreed with the results previously reported by Almugadam that HFD/STZ could lead to weight loss (Almugadam et al., 2021). Then, the weight of HFD/STZ mice was increased after L-50 treatment. Notably, the recovered weights of the mice in the high-concentration (HC) L-50 group were close to the normal group. In contrast, mice in the Rosi and CoQ10 groups were unable to reverse the trend of weight loss, which were consistent with the reported findings (Derosa et al., 2009; Boots et al., 2016). These results indicated that L-50 could reverse the trend of weight loss in HFD/STZ mice, suggesting that L-50 might be a good therapeutic agent for diabetes.

### 3.7 Impact of L-50 on glycemia and lipids metabolism in HFD/STZ mice

HFD/STZ mice developed insulin resistance after successful modeling. After L-50 intervention, serum insulin (FINS) levels increased significantly (see Figure 4E), allowing a dramatic decrease in serum fasting blood glucose (FBG) levels in HFD/STZ mice (see Figure 4D). In addition, hepatic glycogen content was significantly decreased in HFD/STZ mice (see Figure 4C), suggesting that the ability of hepatic tissues to absorb and utilize glucose was enhanced. Correspondingly, homeostatic model assessment of insulin resistance (HOMA-IR) calculated by FBG and FINS was also improved (see Figure 4F). HFD/STZ mice were in a hyperlipidemic state after successful modeling. After treatment with L-50, triglyceride (TG), total cholesterol (T-CHO), and high-density lipoprotein (HDL-C) levels were decreased, while low-density lipoprotein (LDL-C) levels was increased (see Figure 4B). Although CoQ10 slightly improved hyperglycemic hyperlipidemia parameters in HFD/STZ mice, the difference failed to reach statistical significance. It was supported by the clinical trial reported by Fallah et al. (Gholnari et al., 2018; Zhang et al., 2018).These results indicated that L-50 could reverse hyperglycemia and hyperlipidemia and maintain the stability of glucose and lipid metabolism, suggesting that L-50 could effectively improve insulin resistance in HFD/STZ mice, further revealing the potential of L-50 as a therapeutic agent for T2DM.

## 4 Conclusion

In summary, a novel CoQ10 derivative was successfully obtained after the modification of CoQ10 terminus. Cellular assays showed that L-50 had low toxicity and increased glucose consumption by IR-HepG2 to improve cyto-insulin resistance. Moreover, animal experiments revealed that L-50 could reverse the weight of HFD/STZ mice and regulate the metabolism of glycemia and lipids, which included hepatic glycogen, FBG, FINS, HOMA-IR, TG, T-CHO, LDL-C, and HDL-C in HFD/STZ mice. Furthermore, L-50 could return insulin resistance to a normal homeostasis, possibly attenuating oxidative stress by reducing the accumulation of ROS, as well as engaging the JNK/AKT/GSK3β protein signaling pathway to alleviate insulin resistance. Therefore, L-50, a novel CoQ10 derivative, has a very promising future as an anti-hyperglycemic agent to help insulin-resistant T2DM patients.

## Data Availability

The original contributions presented in the study are included in the article/Supplementary Material, further inquiries can be directed to the corresponding authors.
