# Supplementary figures and images for: Investigation of anti-diabetic effect of a novel coenzyme Q10 derivative
Source: Front Chem. 2023 Oct 19;11:1280999. doi: 10.3389/fchem.2023.1280999 (PMC10620959; doi:10.3389/fchem.2023.1280999)

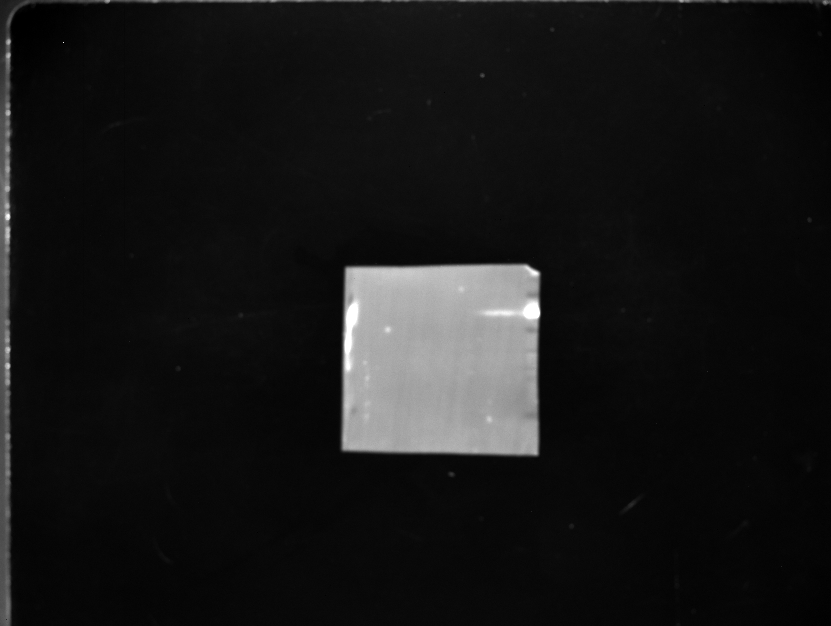

Supplement: Supplementary file 1 [file DataSheet3.ZIP › WB Original data/AKT/1 2023-05-11_15-49-30_3_16bit.png]

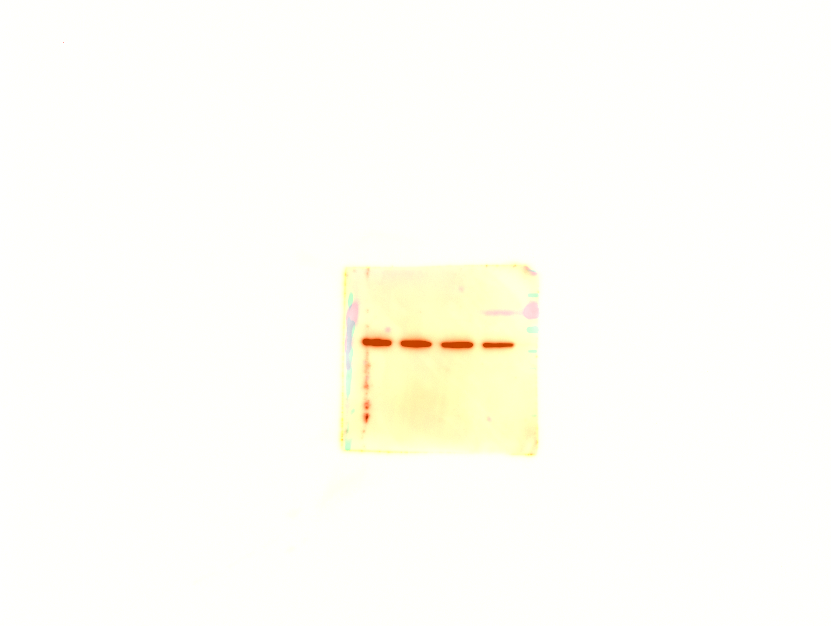

Supplement: Supplementary file 1 [file DataSheet3.ZIP › WB Original data/AKT/1 Actin with marker.png]

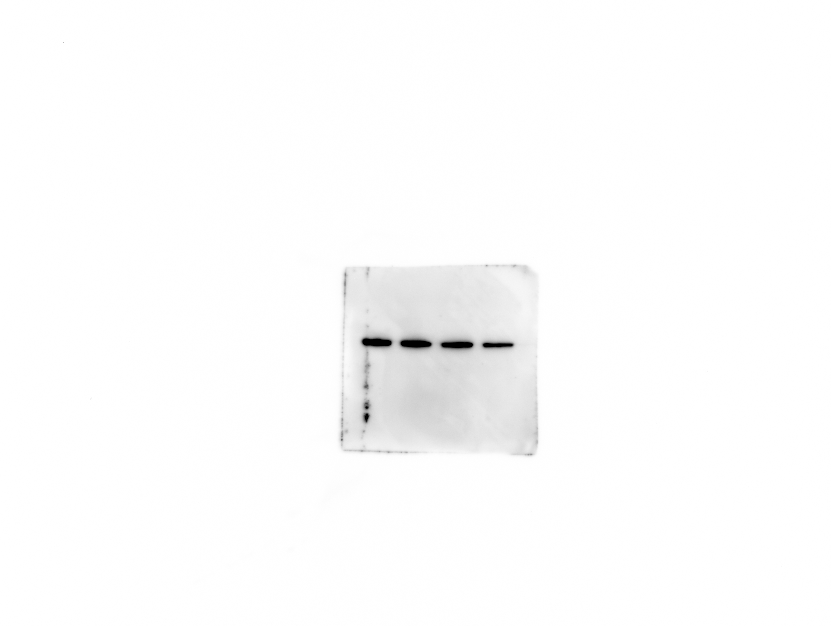

Supplement: Supplementary file 1 [file DataSheet3.ZIP › WB Original data/AKT/1 Actin.png]

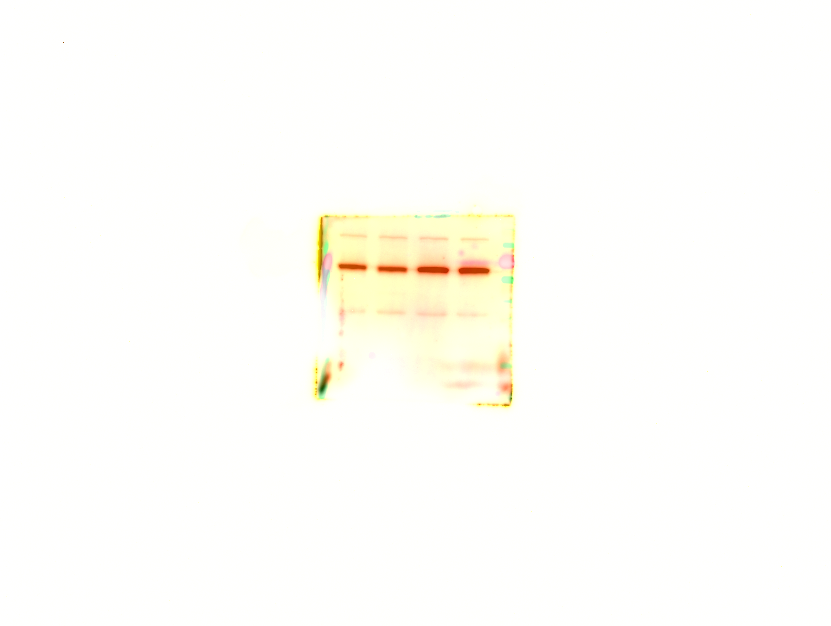

Supplement: Supplementary file 1 [file DataSheet3.ZIP › WB Original data/AKT/1 AKT with marker.png]

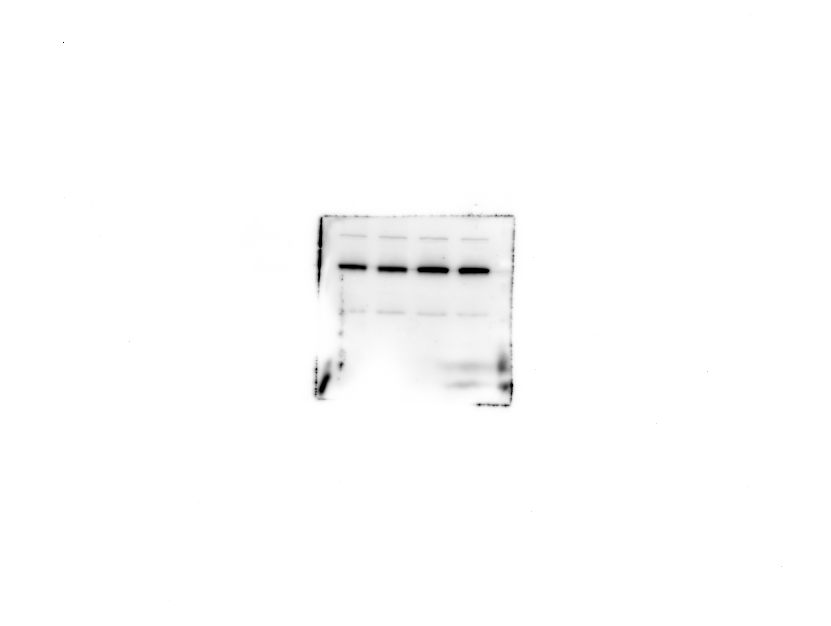

Supplement: Supplementary file 1 [file DataSheet3.ZIP › WB Original data/AKT/1 AKT.png]

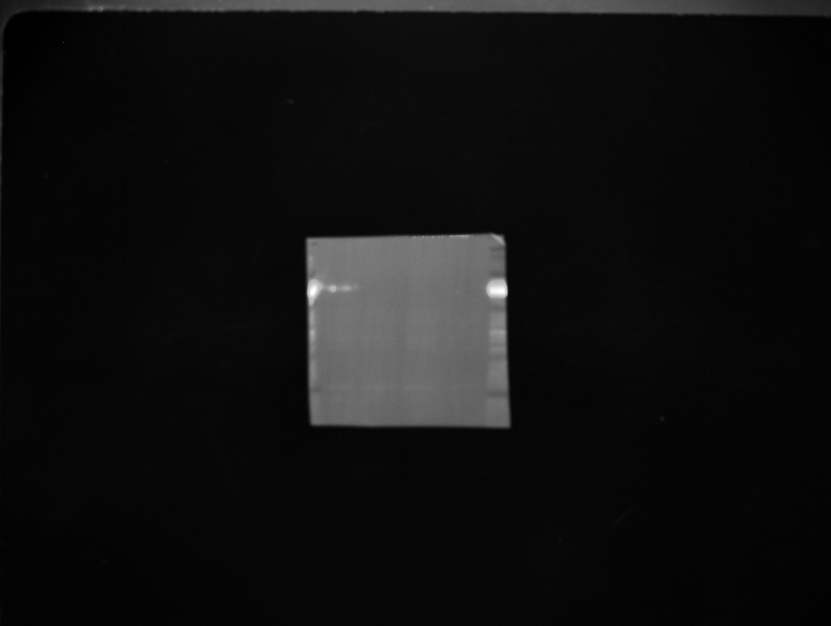

Supplement: Supplementary file 1 [file DataSheet3.ZIP › WB Original data/AKT/2 2023-05-03_18-32-50 5M_3_16bit.png]

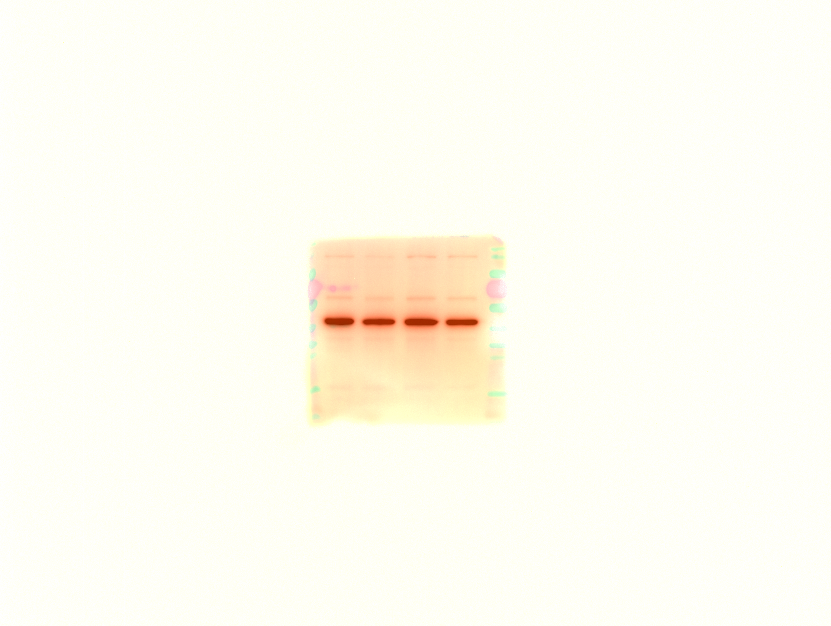

Supplement: Supplementary file 1 [file DataSheet3.ZIP › WB Original data/AKT/2 Actin with marker.png]

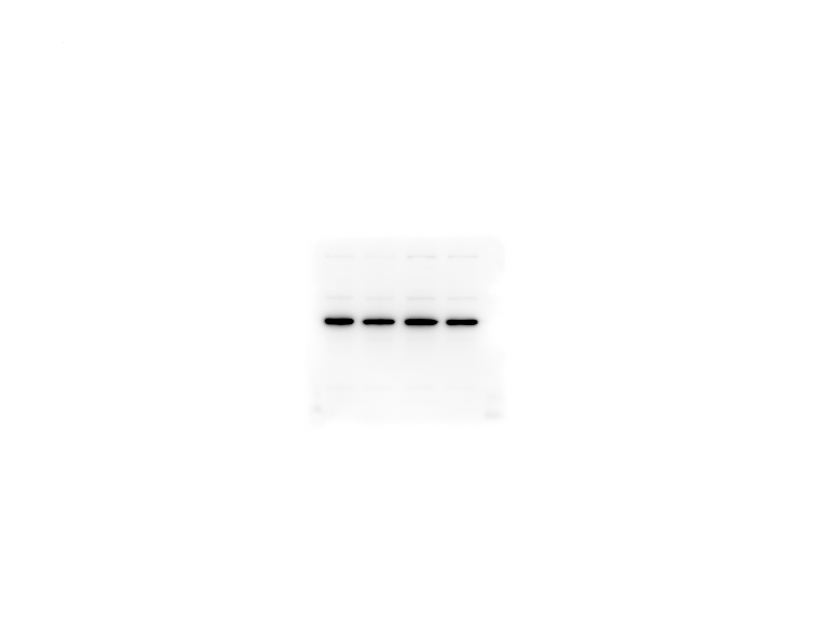

Supplement: Supplementary file 1 [file DataSheet3.ZIP › WB Original data/AKT/2 Actin.png]

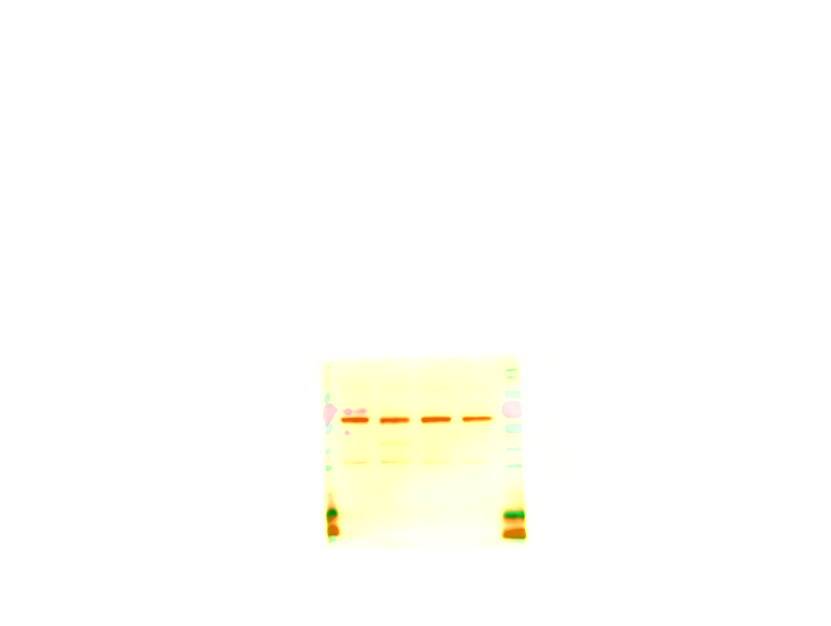

Supplement: Supplementary file 1 [file DataSheet3.ZIP › WB Original data/AKT/2 AKT with marker.png]

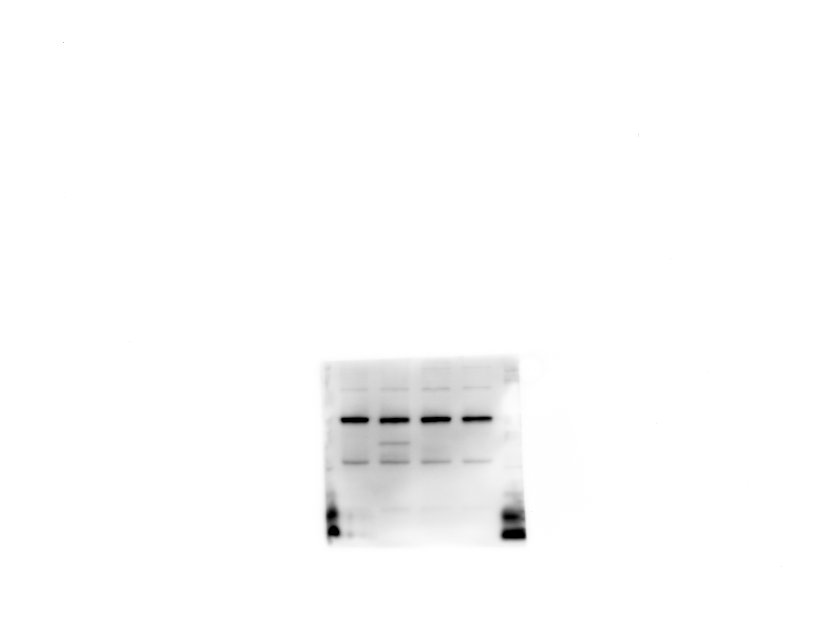

Supplement: Supplementary file 1 [file DataSheet3.ZIP › WB Original data/AKT/2 AKT.png]

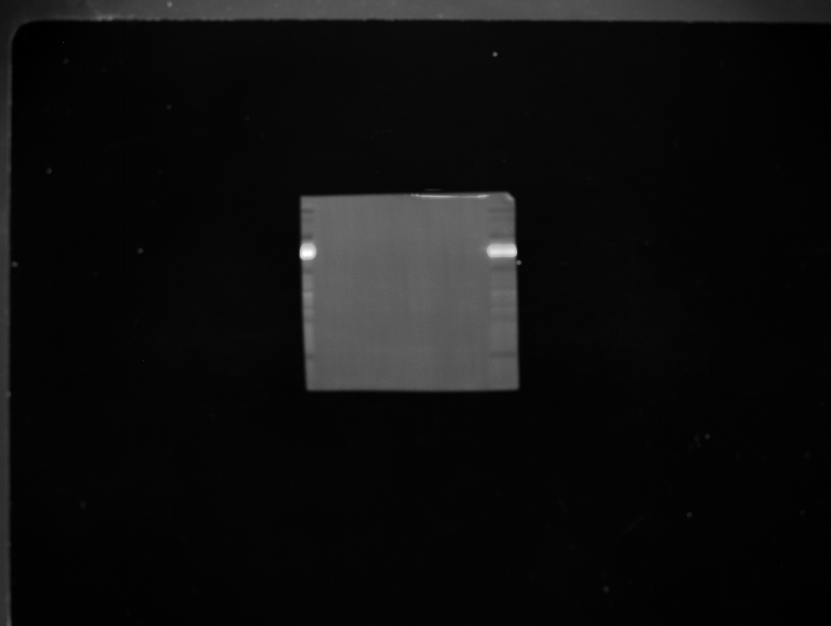

Supplement: Supplementary file 1 [file DataSheet3.ZIP › WB Original data/AKT/3 2023-05-03_18-37-25 6M_3_16bit.png]

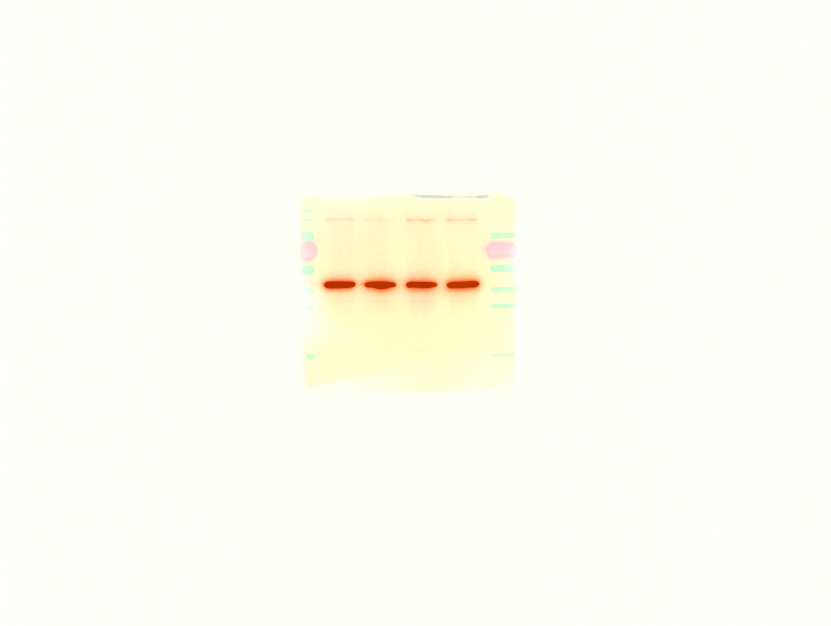

Supplement: Supplementary file 1 [file DataSheet3.ZIP › WB Original data/AKT/3 Actin with marker.png]

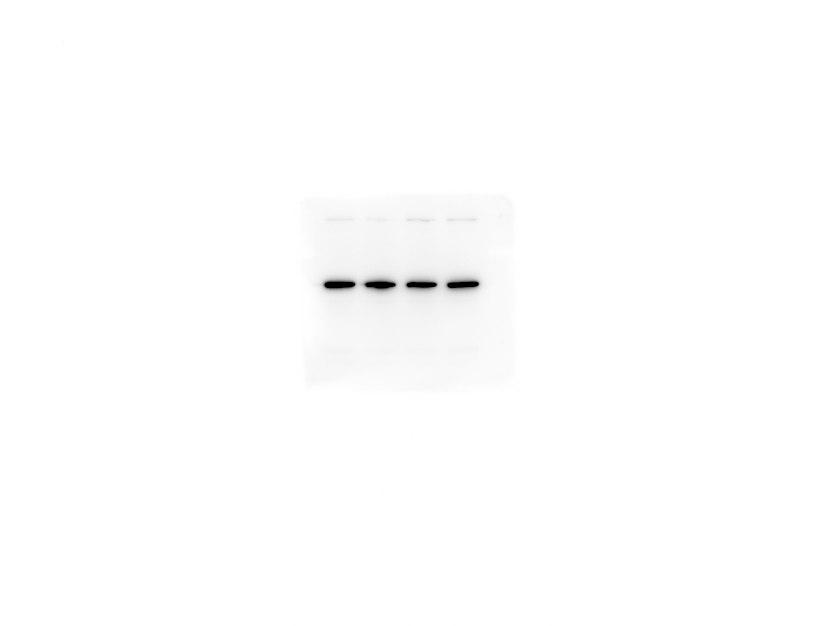

Supplement: Supplementary file 1 [file DataSheet3.ZIP › WB Original data/AKT/3 Actin.png]

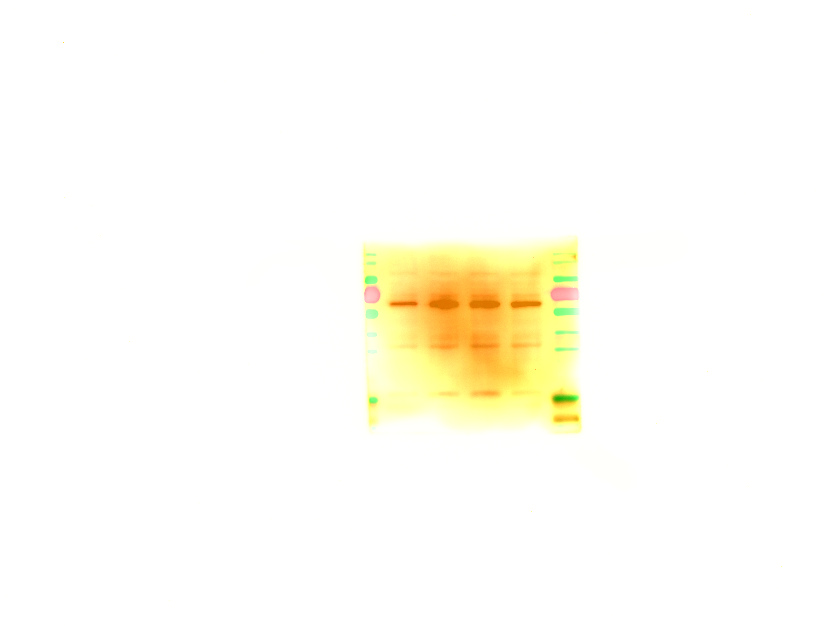

Supplement: Supplementary file 1 [file DataSheet3.ZIP › WB Original data/AKT/3 AKT with marker.png]

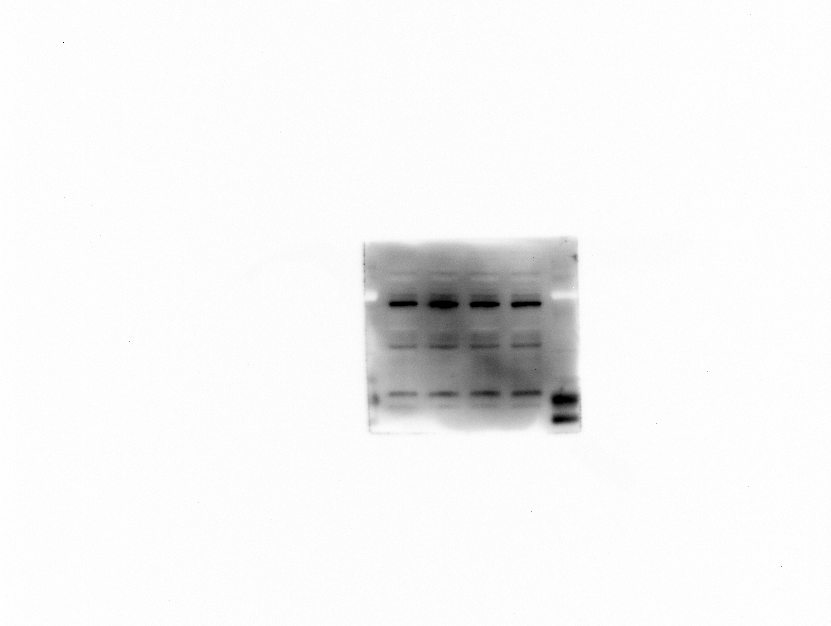

Supplement: Supplementary file 1 [file DataSheet3.ZIP › WB Original data/AKT/3 AKT.png]

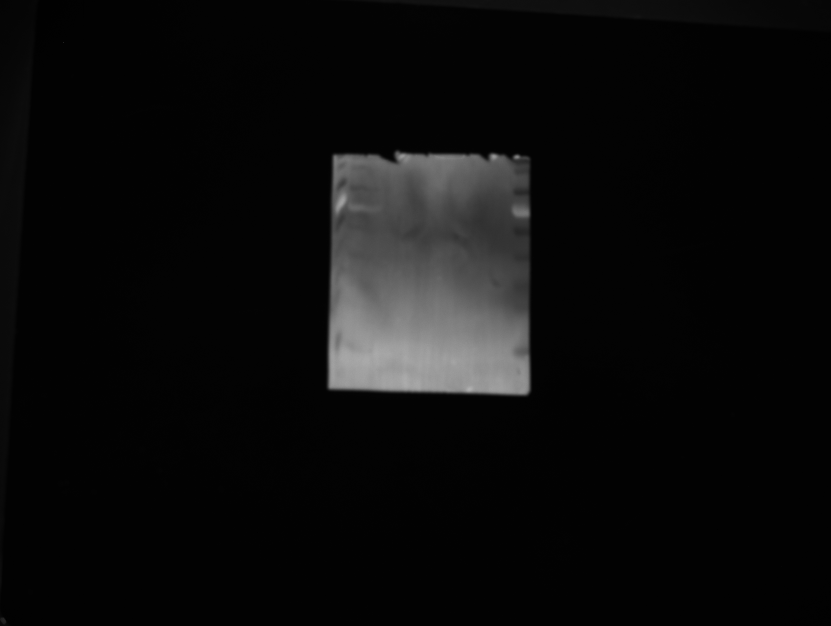

Supplement: Supplementary file 1 [file DataSheet3.ZIP › WB Original data/GSK3β/1 2023-04-05_21-10-20 _16bit.png]

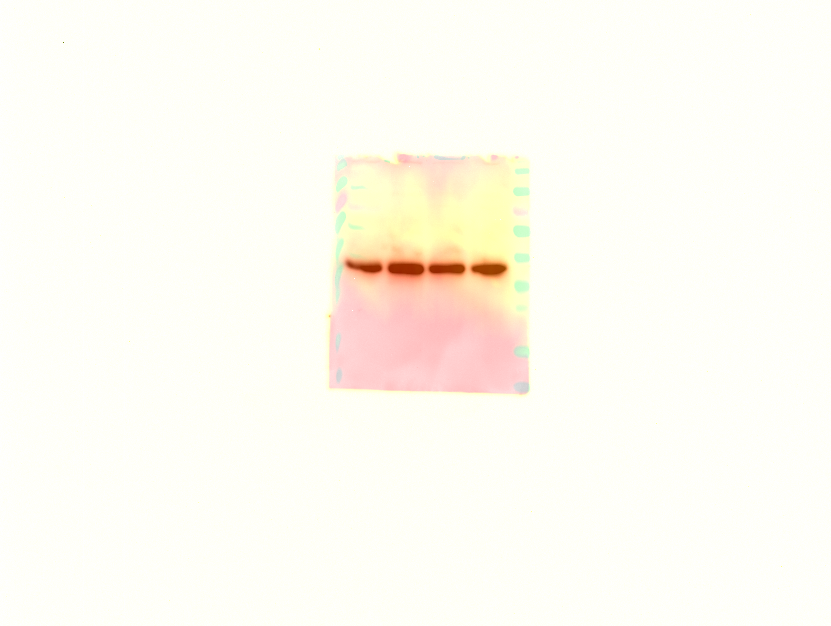

Supplement: Supplementary file 1 [file DataSheet3.ZIP › WB Original data/GSK3β/1 GAPDH with marker.png]

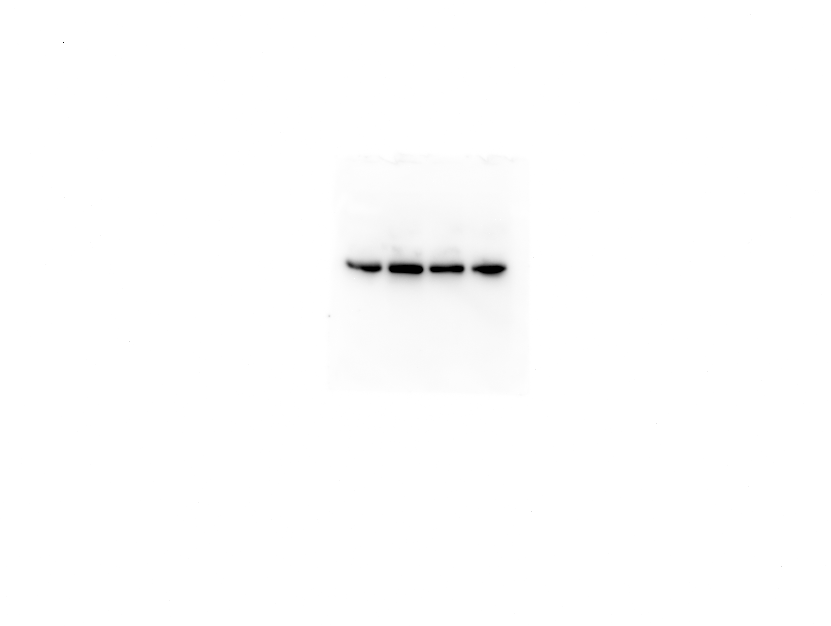

Supplement: Supplementary file 1 [file DataSheet3.ZIP › WB Original data/GSK3β/1GAPDH.png]

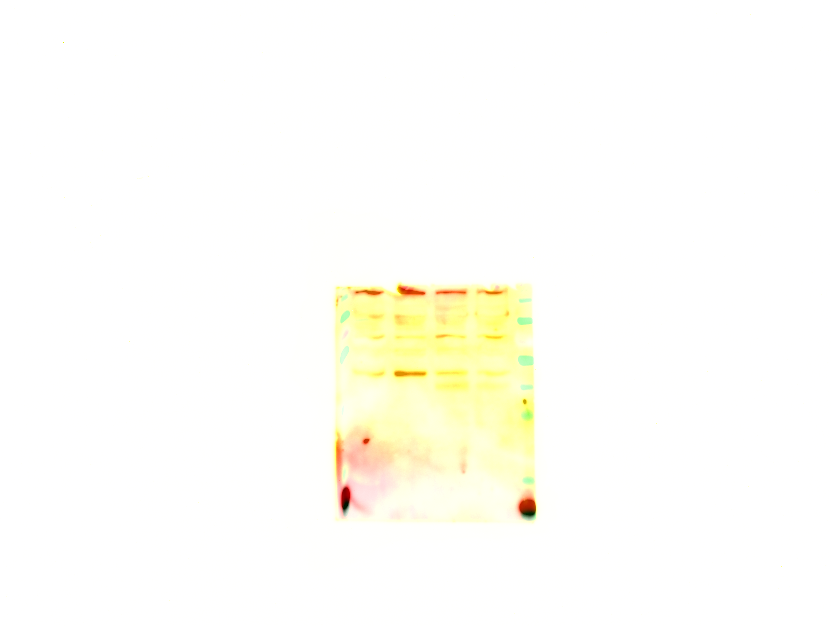

Supplement: Supplementary file 1 [file DataSheet3.ZIP › WB Original data/GSK3β/1Gsk3β with marker.png]

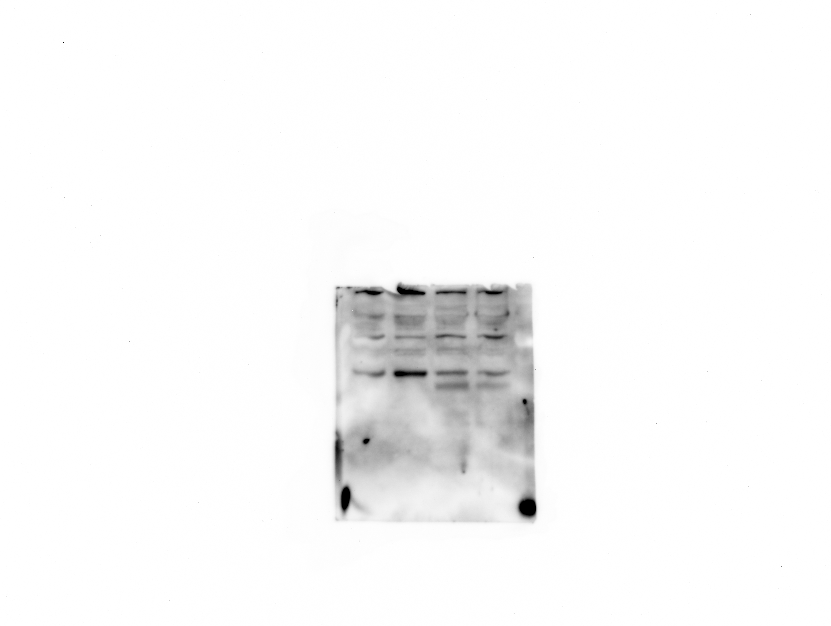

Supplement: Supplementary file 1 [file DataSheet3.ZIP › WB Original data/GSK3β/1Gsk3β.png]

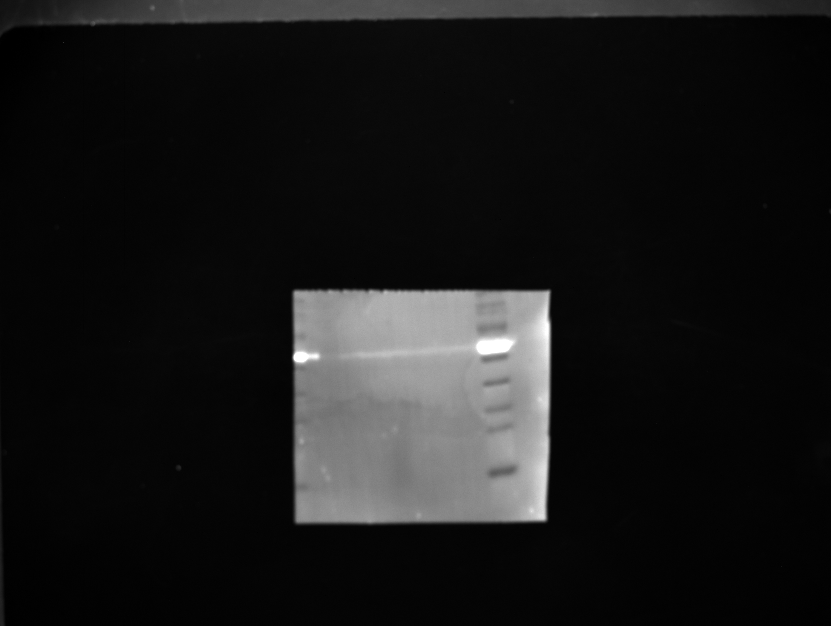

Supplement: Supplementary file 1 [file DataSheet3.ZIP › WB Original data/GSK3β/2 2023-04-09_15-49-46 2_16bit.png]

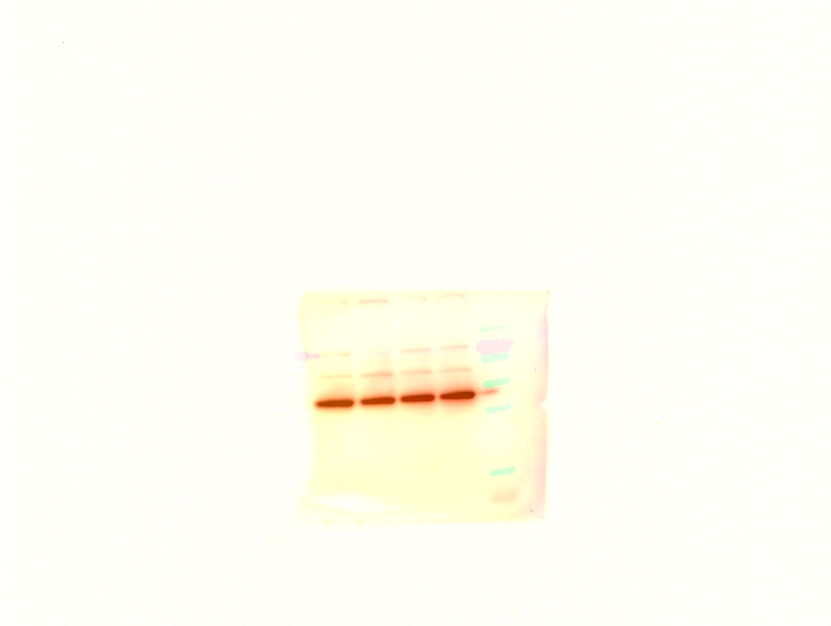

Supplement: Supplementary file 1 [file DataSheet3.ZIP › WB Original data/GSK3β/2 GAPDH with marker.png]

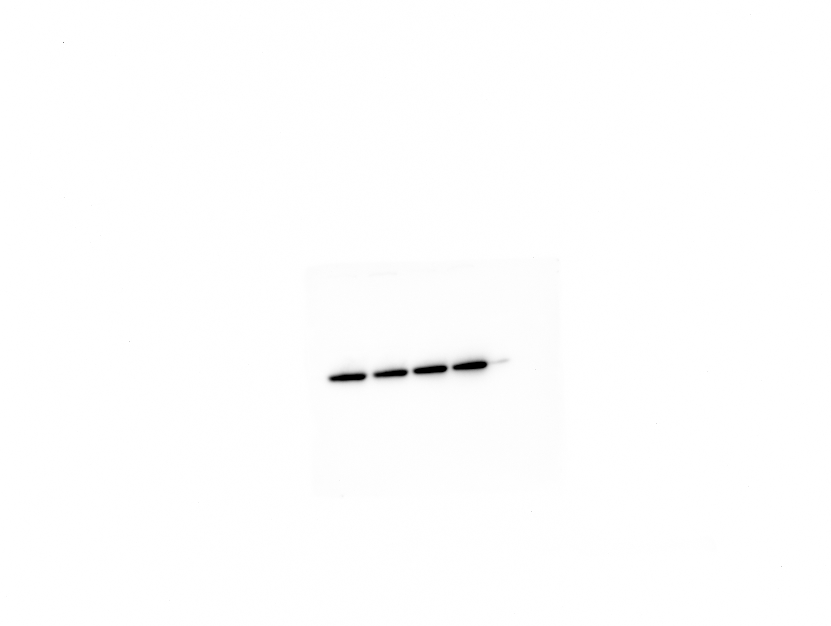

Supplement: Supplementary file 1 [file DataSheet3.ZIP › WB Original data/GSK3β/2 GAPDH.png]

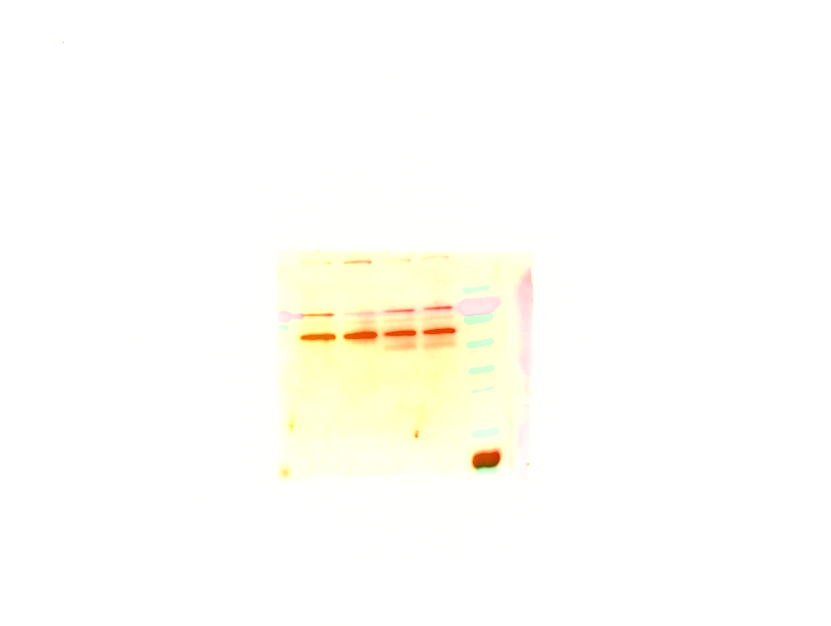

Supplement: Supplementary file 1 [file DataSheet3.ZIP › WB Original data/GSK3β/2 GSK3β with marker.png]

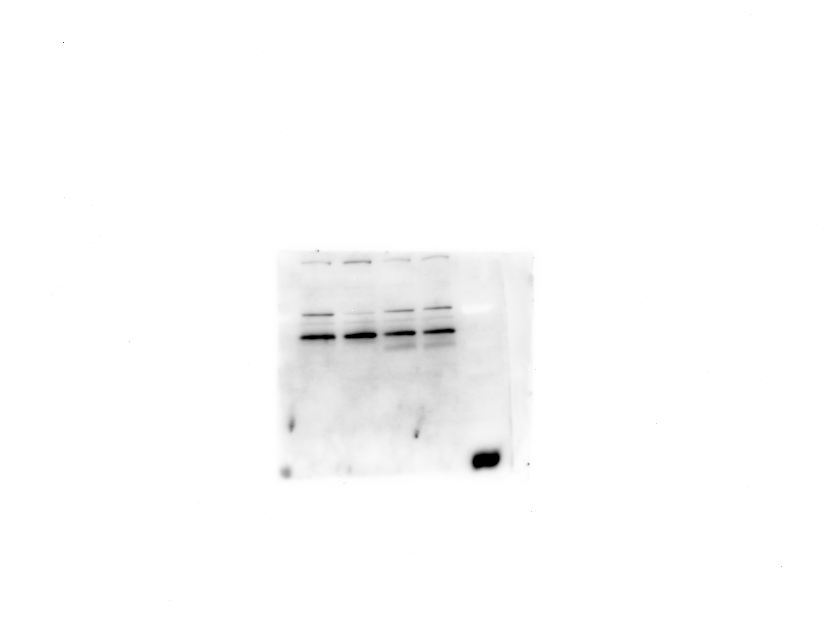

Supplement: Supplementary file 1 [file DataSheet3.ZIP › WB Original data/GSK3β/2 GSK3β.png]

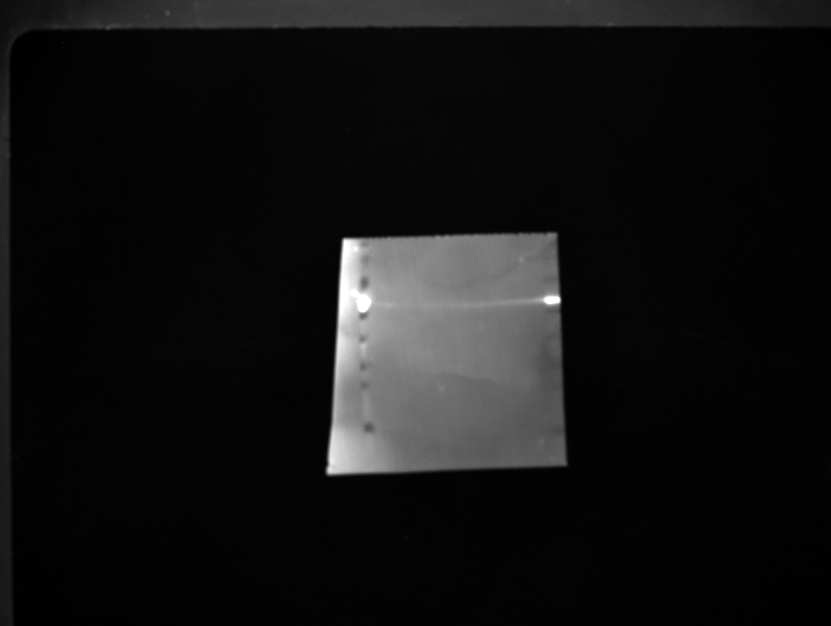

Supplement: Supplementary file 1 [file DataSheet3.ZIP › WB Original data/GSK3β/3 2023-04-09_15-54-35 _16bit.png]

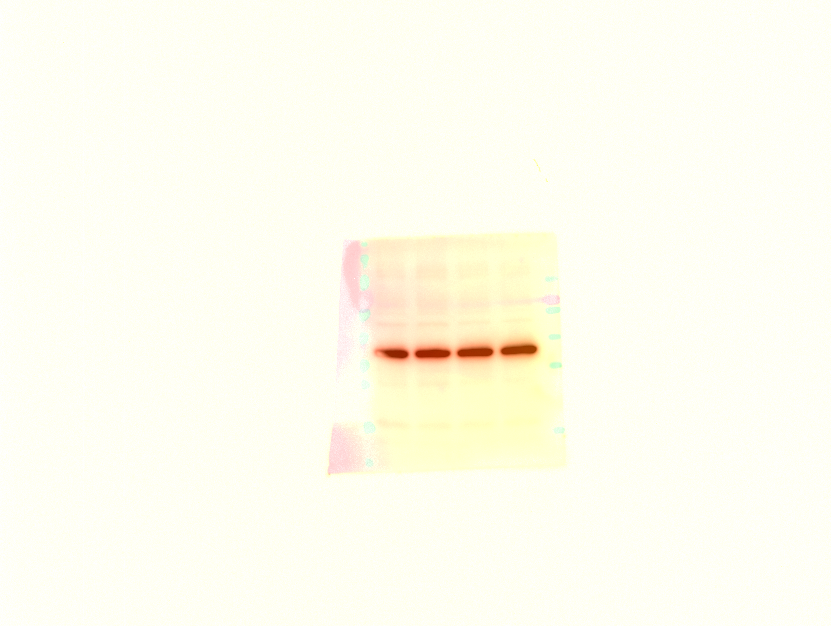

Supplement: Supplementary file 1 [file DataSheet3.ZIP › WB Original data/GSK3β/3 GAPDH with marker.png]

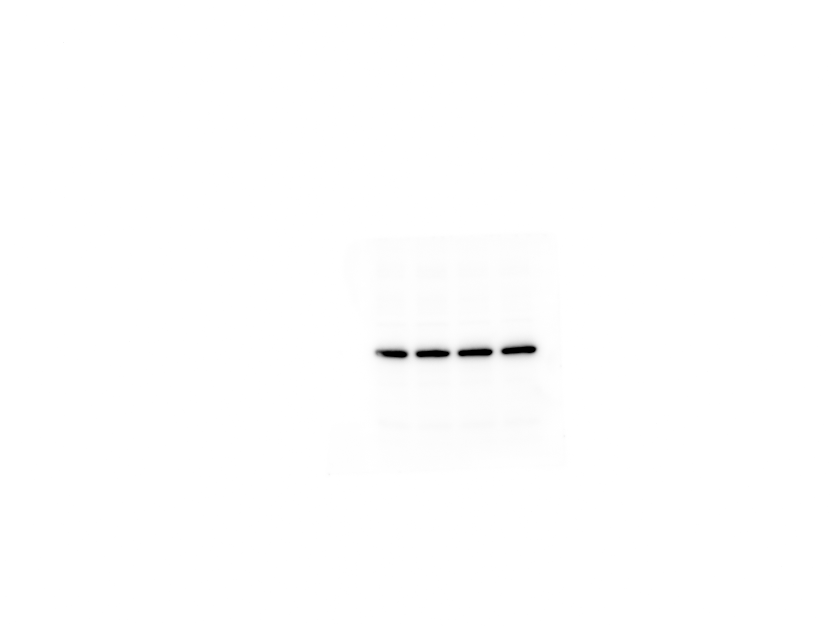

Supplement: Supplementary file 1 [file DataSheet3.ZIP › WB Original data/GSK3β/3 GAPDH.png]

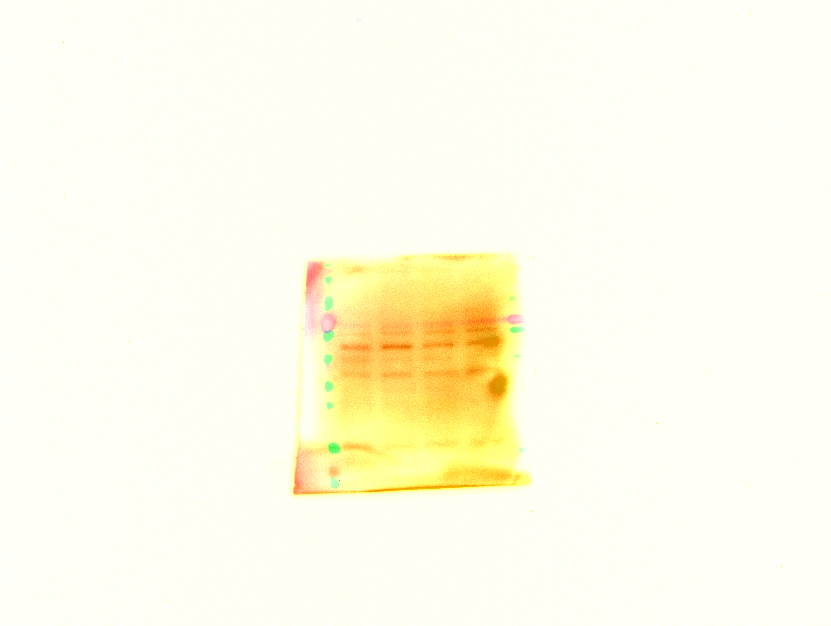

Supplement: Supplementary file 1 [file DataSheet3.ZIP › WB Original data/GSK3β/3 GSK3β with marker.png]

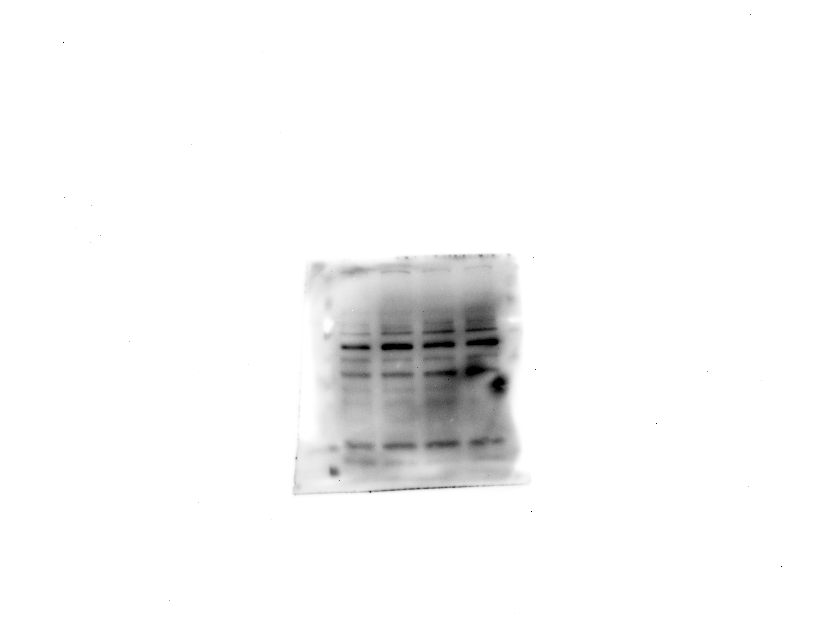

Supplement: Supplementary file 1 [file DataSheet3.ZIP › WB Original data/GSK3β/3 GSK3β.png]

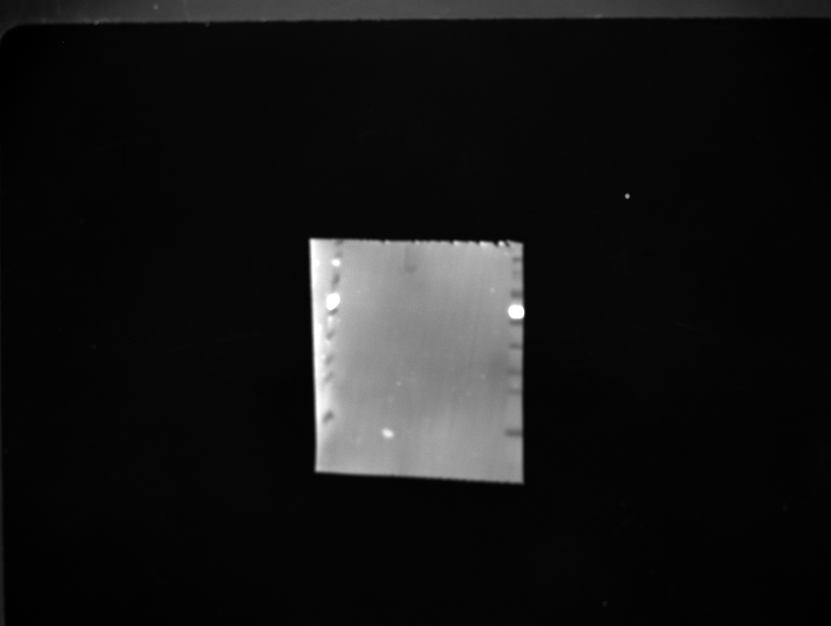

Supplement: Supplementary file 1 [file DataSheet3.ZIP › WB Original data/JNK/1 2023-04-09_16-06-15 _16bit.png]

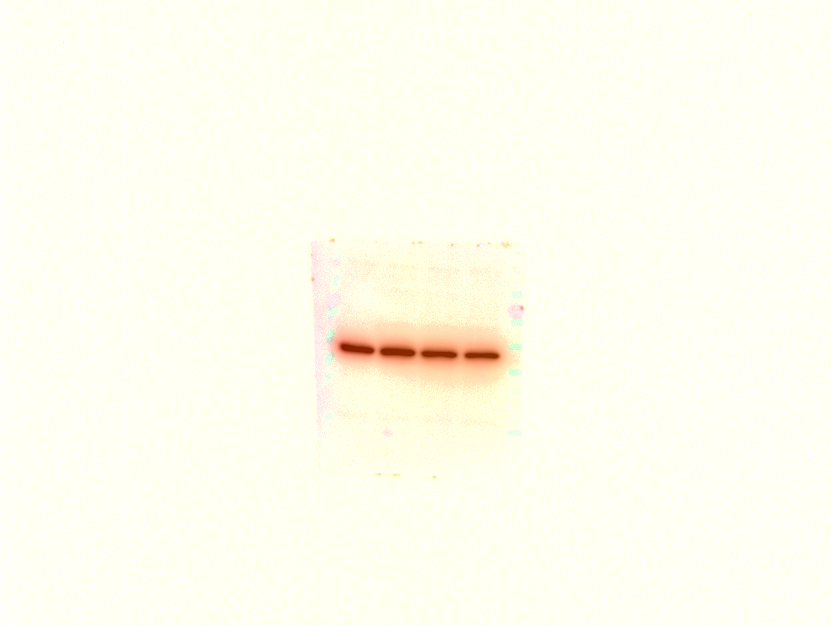

Supplement: Supplementary file 1 [file DataSheet3.ZIP › WB Original data/JNK/1 GAPDH with marker.png]

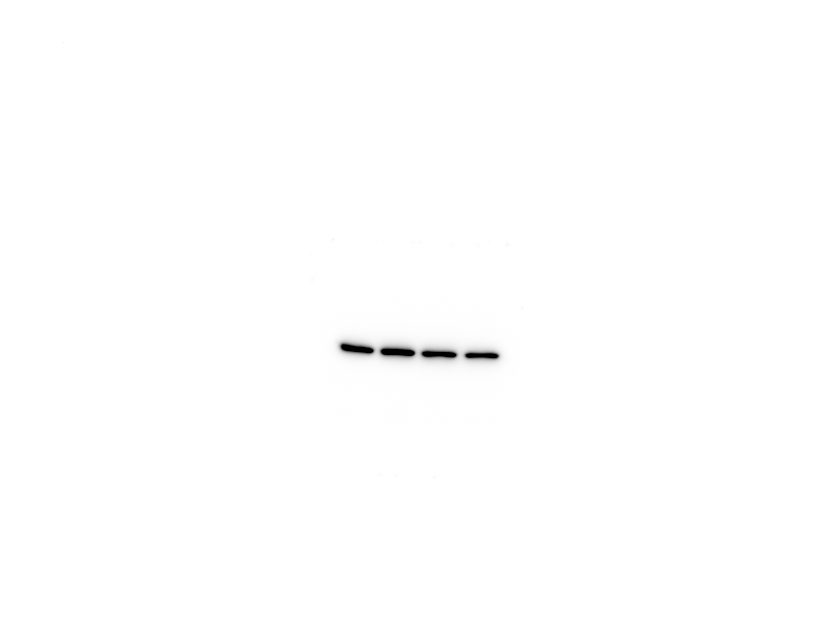

Supplement: Supplementary file 1 [file DataSheet3.ZIP › WB Original data/JNK/1 GAPDH.png]

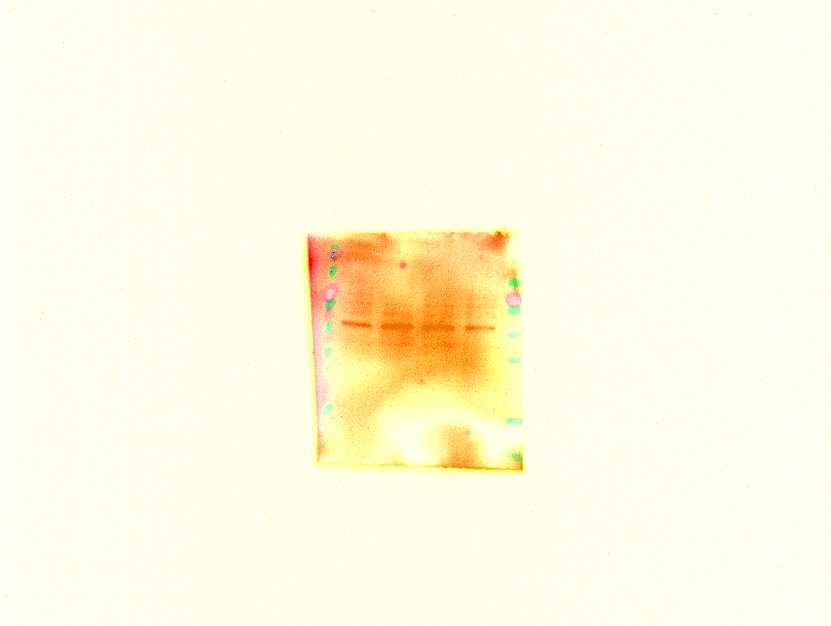

Supplement: Supplementary file 1 [file DataSheet3.ZIP › WB Original data/JNK/1 jnk with marker.png]

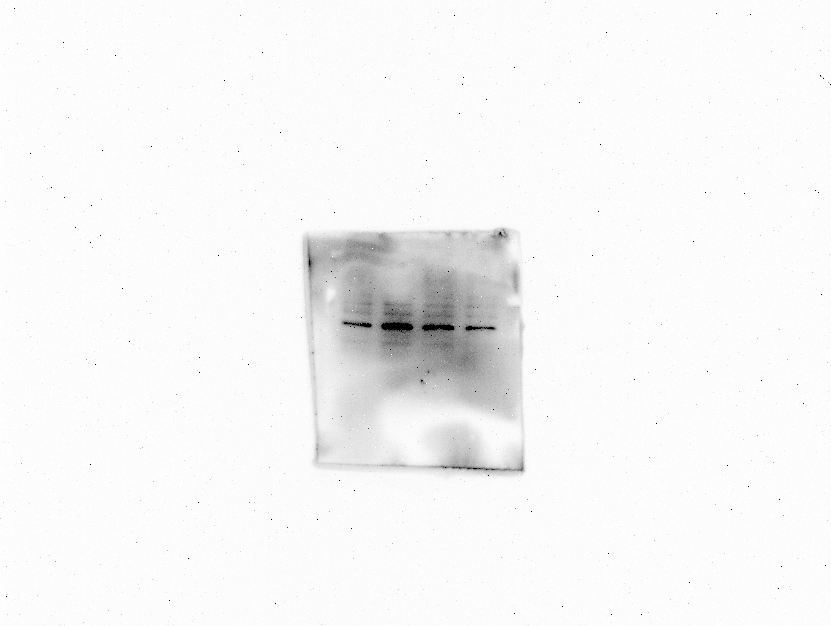

Supplement: Supplementary file 1 [file DataSheet3.ZIP › WB Original data/JNK/1 jnk.png]

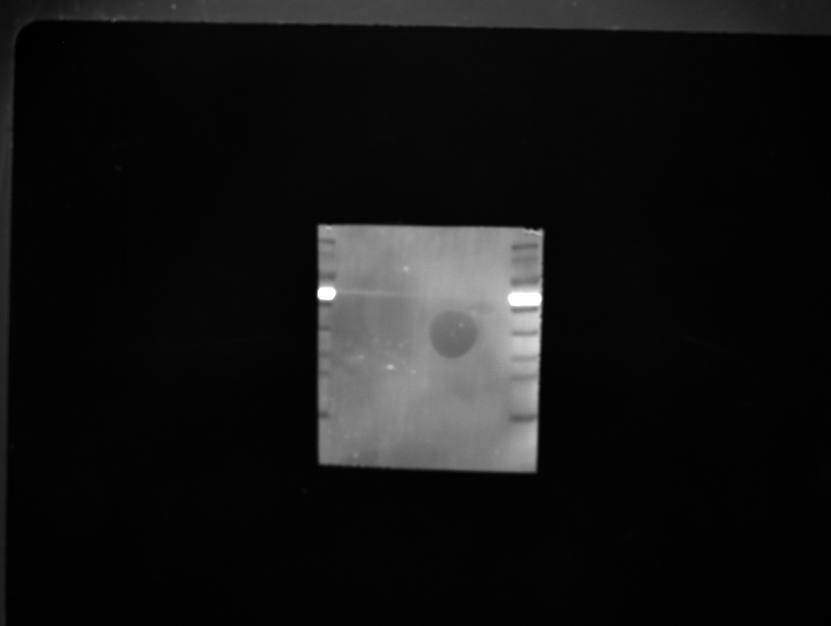

Supplement: Supplementary file 1 [file DataSheet3.ZIP › WB Original data/JNK/2 2023-04-09_16-10-36 16bit.png]

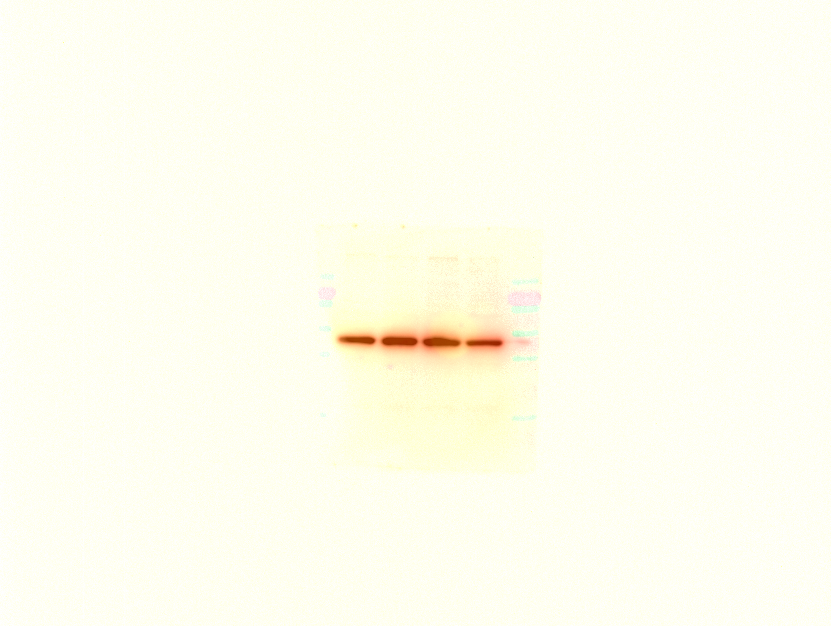

Supplement: Supplementary file 1 [file DataSheet3.ZIP › WB Original data/JNK/2 GAPDH with marker.png]

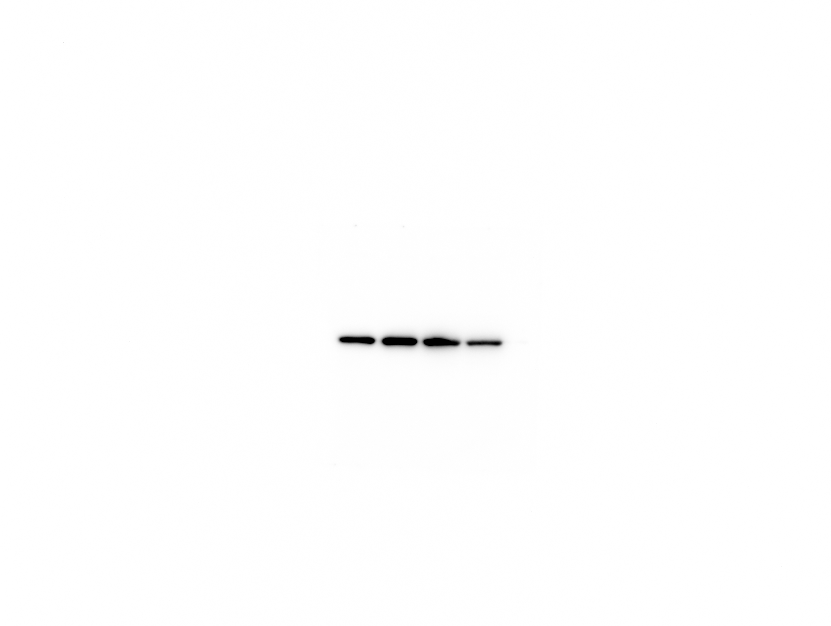

Supplement: Supplementary file 1 [file DataSheet3.ZIP › WB Original data/JNK/2 GAPDH.png]

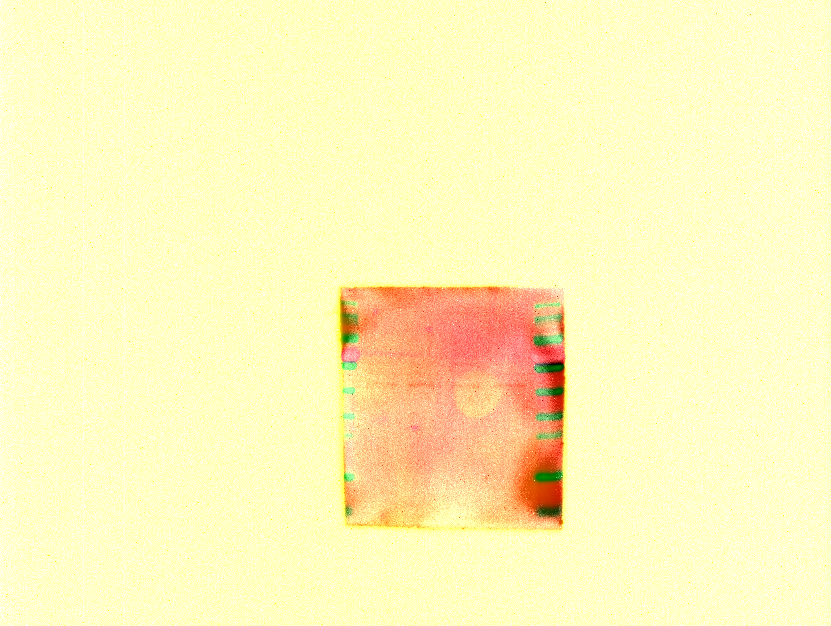

Supplement: Supplementary file 1 [file DataSheet3.ZIP › WB Original data/JNK/2 jnk with marker.png]

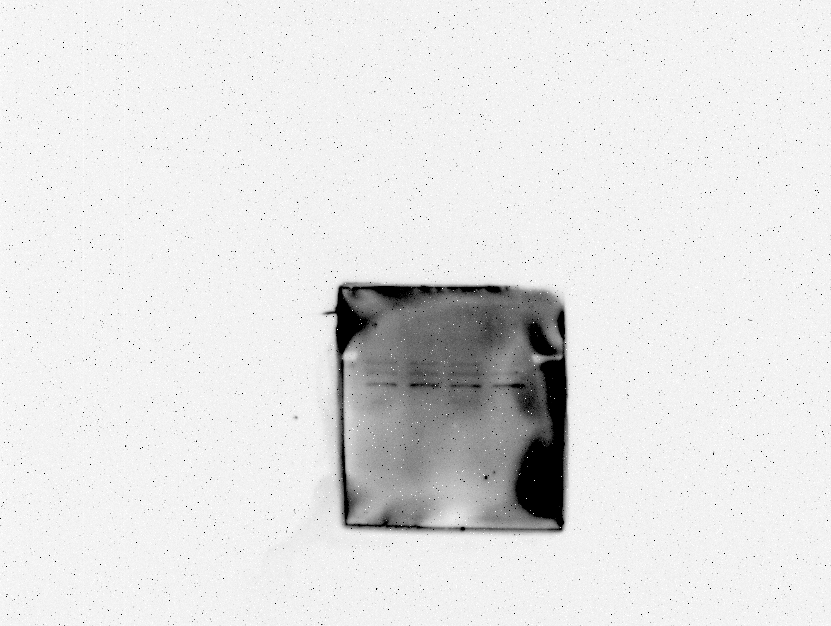

Supplement: Supplementary file 1 [file DataSheet3.ZIP › WB Original data/JNK/2 jnk.png]

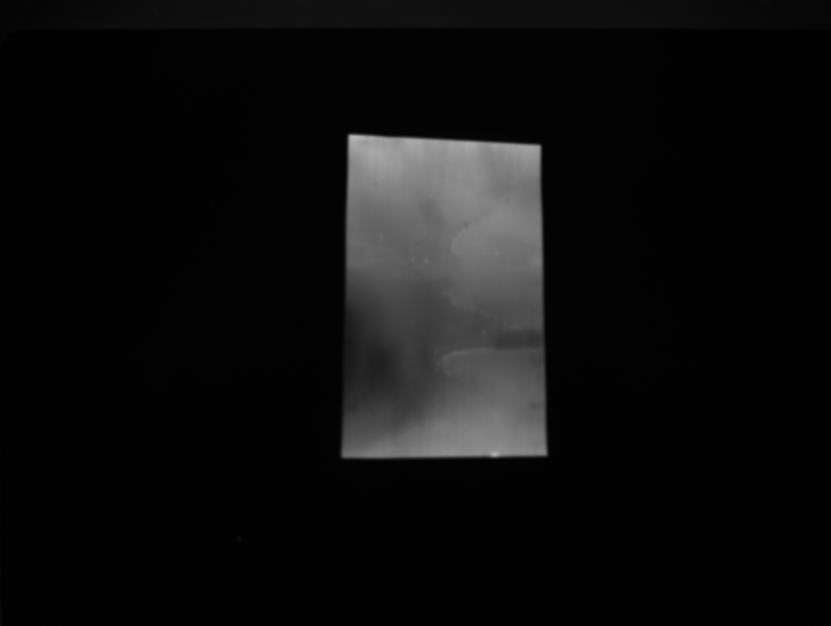

Supplement: Supplementary file 1 [file DataSheet3.ZIP › WB Original data/JNK/3 2023-04-04_21-25-05 _16bit.png]

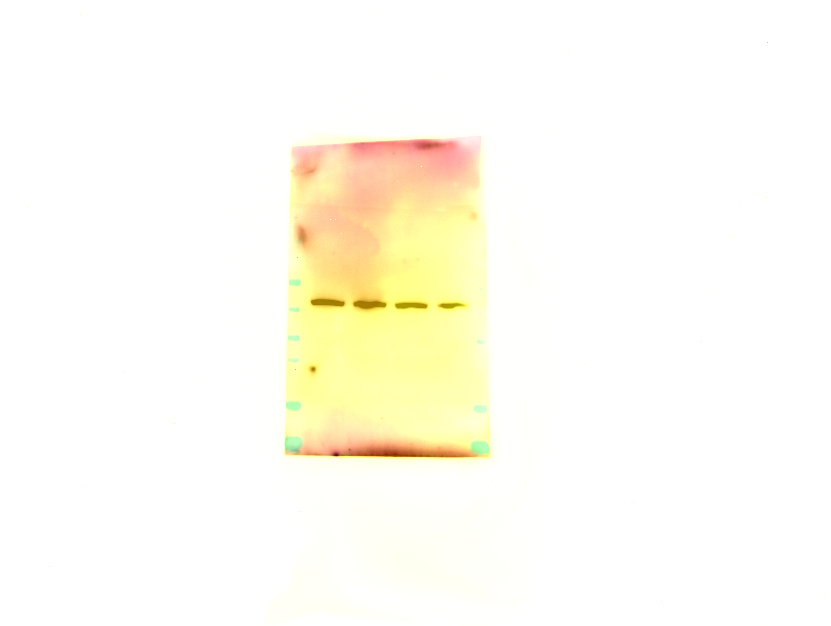

Supplement: Supplementary file 1 [file DataSheet3.ZIP › WB Original data/JNK/3 GAPDH with marker.png]

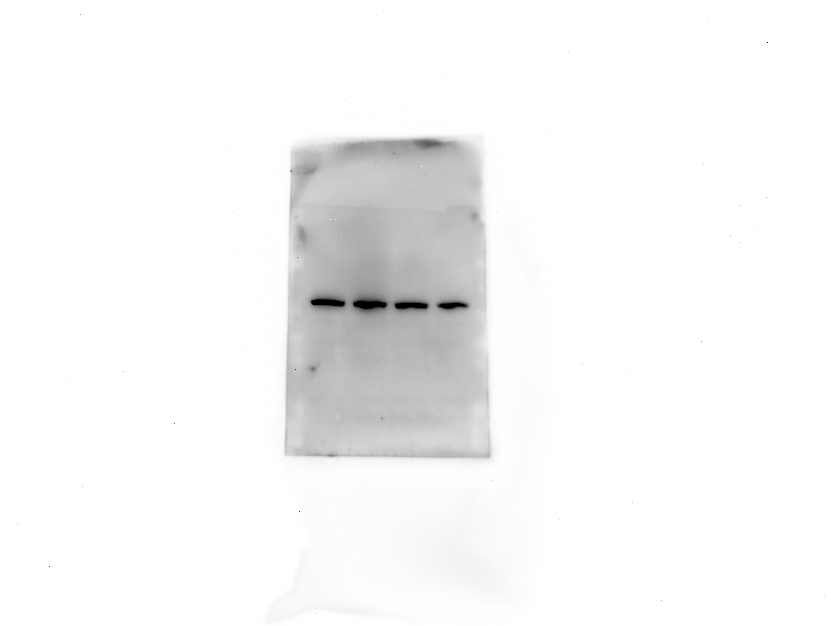

Supplement: Supplementary file 1 [file DataSheet3.ZIP › WB Original data/JNK/3 GAPDH.png]

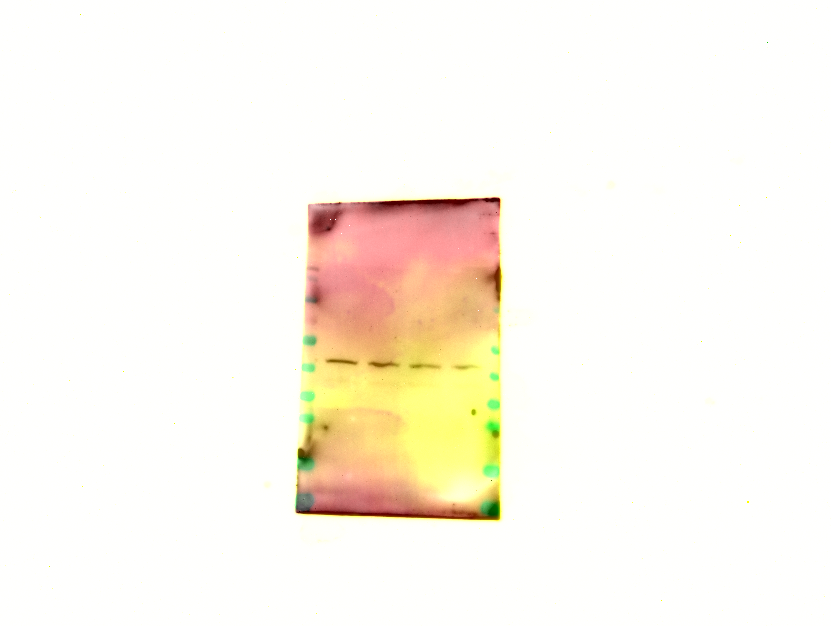

Supplement: Supplementary file 1 [file DataSheet3.ZIP › WB Original data/JNK/3 jnk with marker.png]

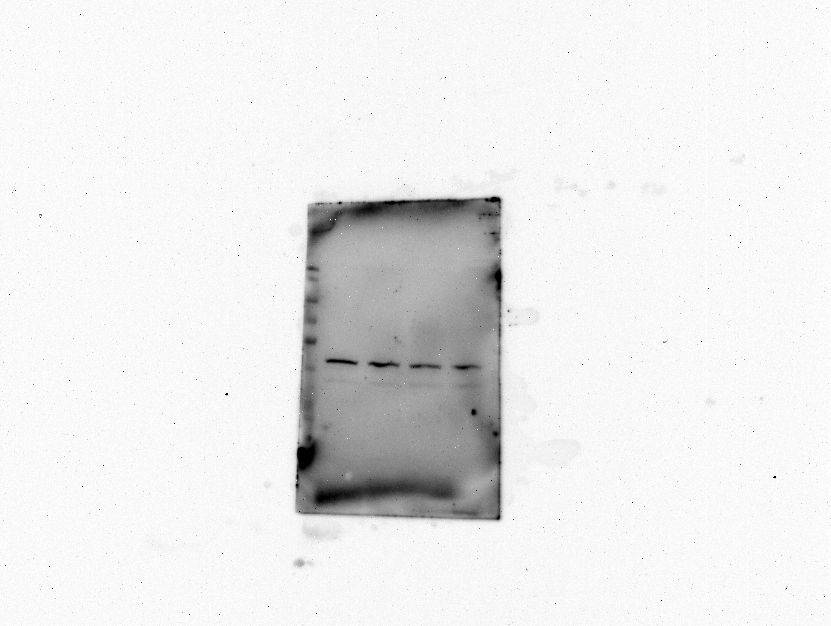

Supplement: Supplementary file 1 [file DataSheet3.ZIP › WB Original data/JNK/3 jnk.png]

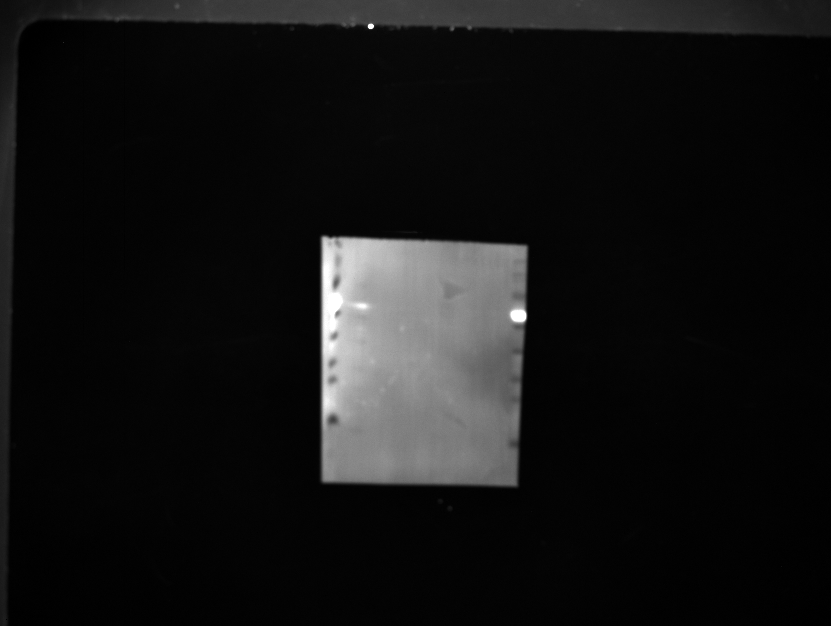

Supplement: Supplementary file 1 [file DataSheet3.ZIP › WB Original data/p-AKT/1 2023-04-08_18-02-17 16bit.png]

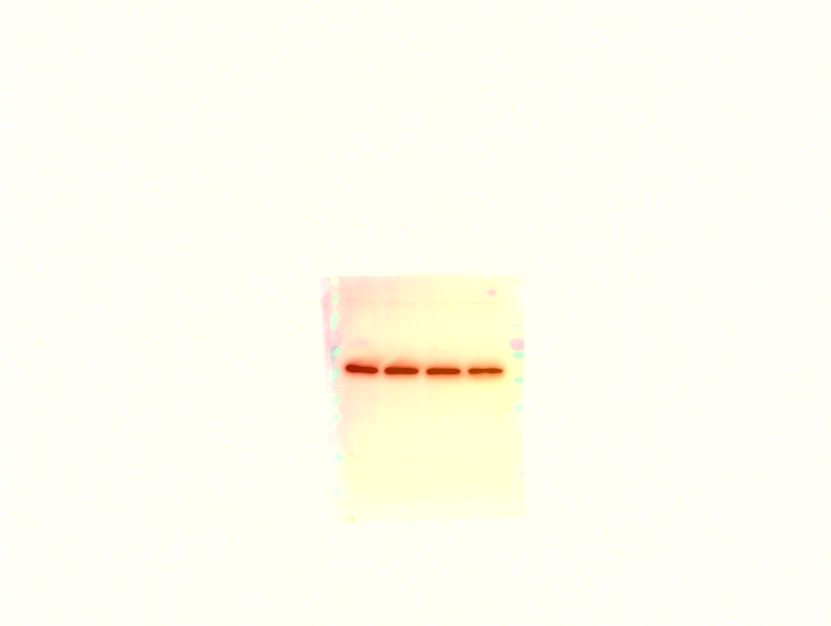

Supplement: Supplementary file 1 [file DataSheet3.ZIP › WB Original data/p-AKT/1 actin with marker.png]

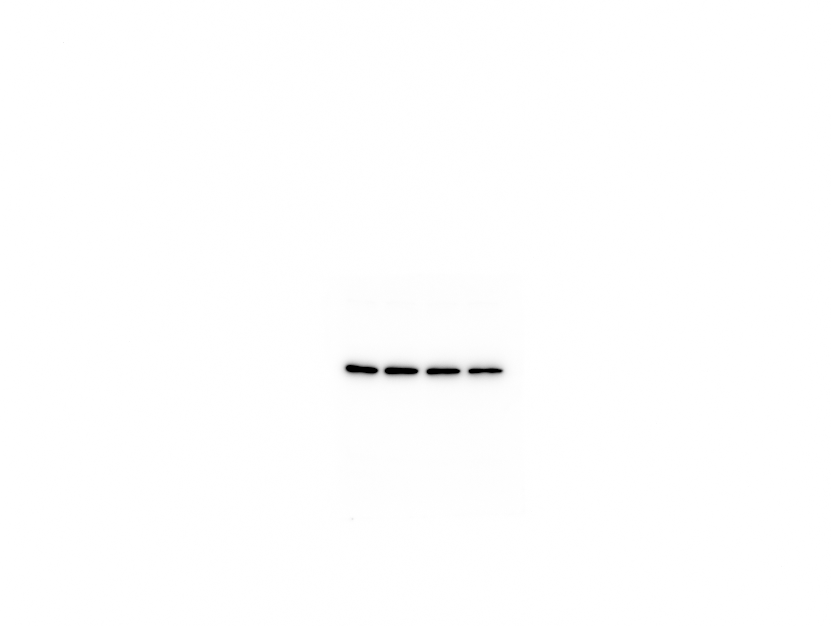

Supplement: Supplementary file 1 [file DataSheet3.ZIP › WB Original data/p-AKT/1 actin.png]

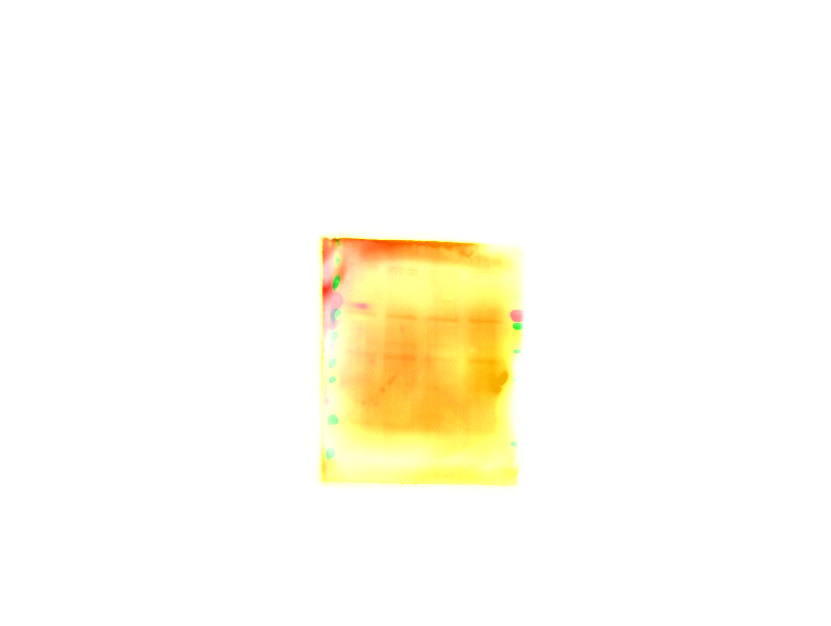

Supplement: Supplementary file 1 [file DataSheet3.ZIP › WB Original data/p-AKT/1 p-akt with marker.png]

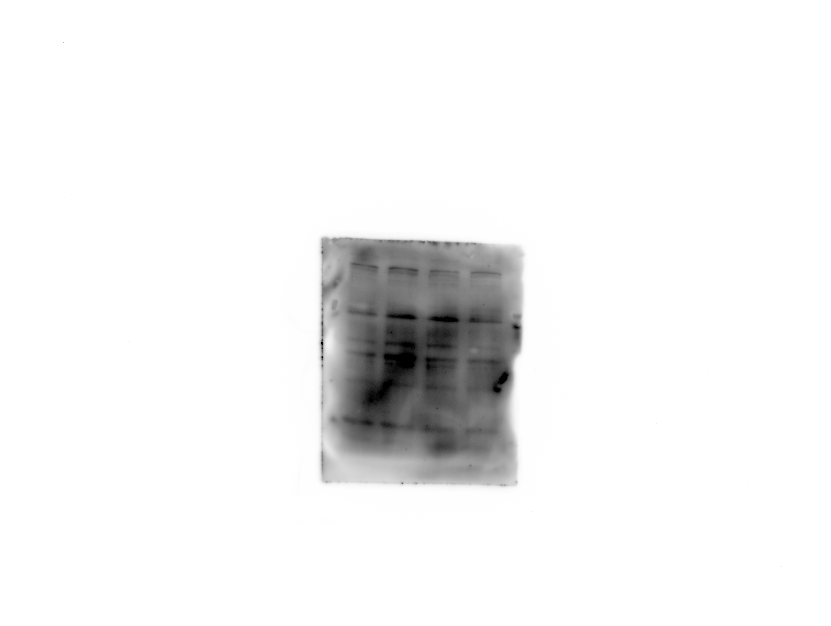

Supplement: Supplementary file 1 [file DataSheet3.ZIP › WB Original data/p-AKT/1 p-akt.png]

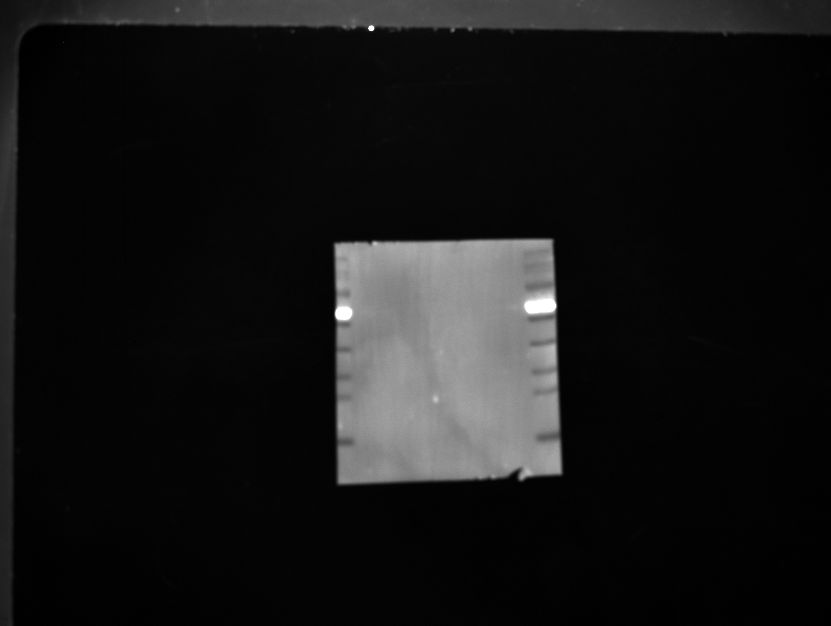

Supplement: Supplementary file 1 [file DataSheet3.ZIP › WB Original data/p-AKT/2 2023-04-08_18-08-39 16bit.png]

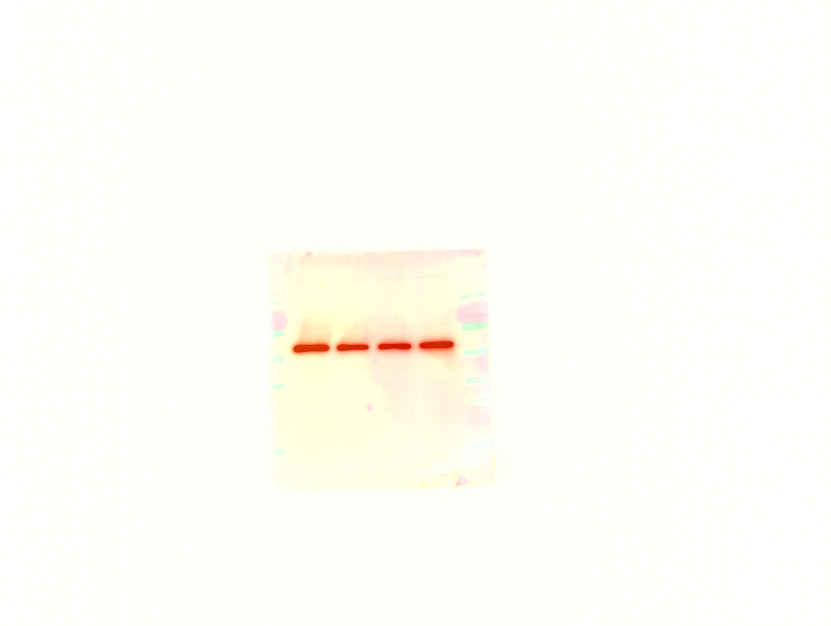

Supplement: Supplementary file 1 [file DataSheet3.ZIP › WB Original data/p-AKT/2 actin with marker.png]

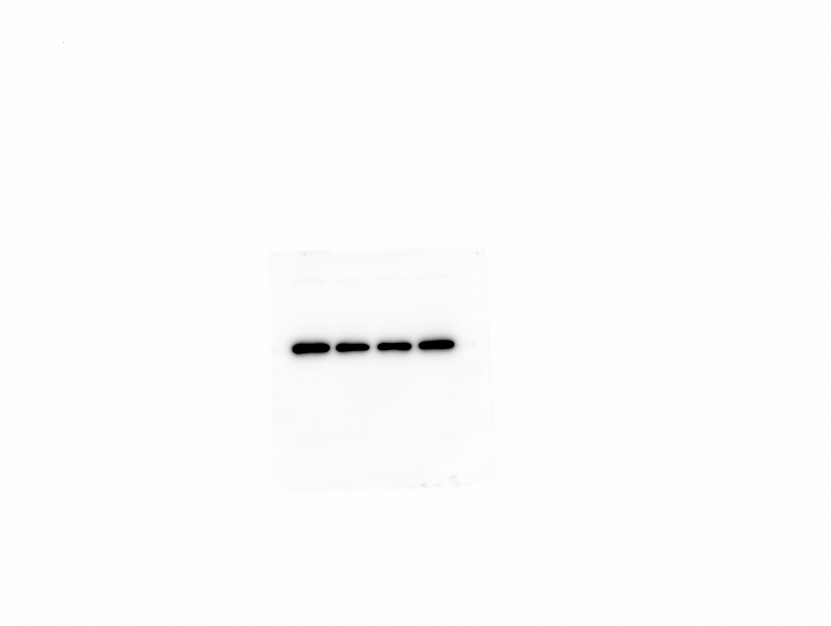

Supplement: Supplementary file 1 [file DataSheet3.ZIP › WB Original data/p-AKT/2 actin.png]

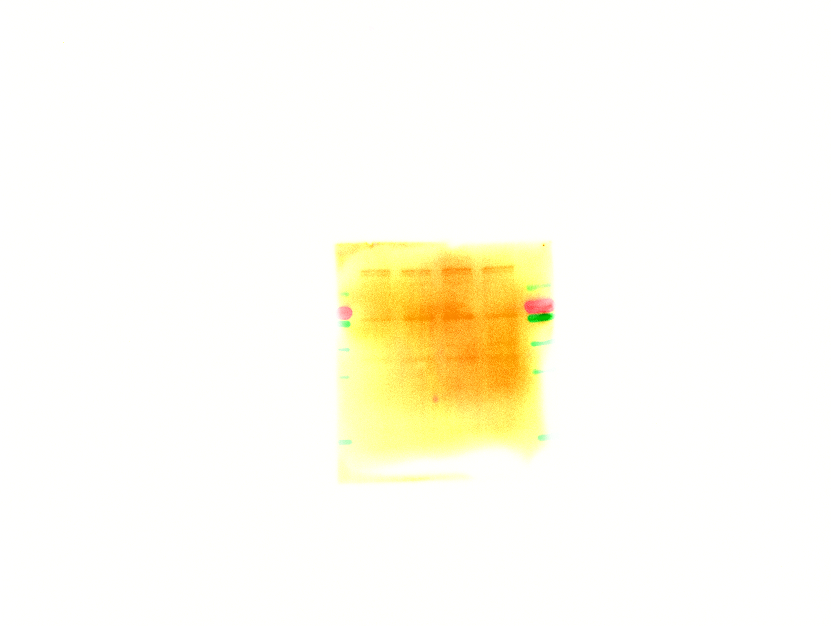

Supplement: Supplementary file 1 [file DataSheet3.ZIP › WB Original data/p-AKT/2 p-akt with marker.png]

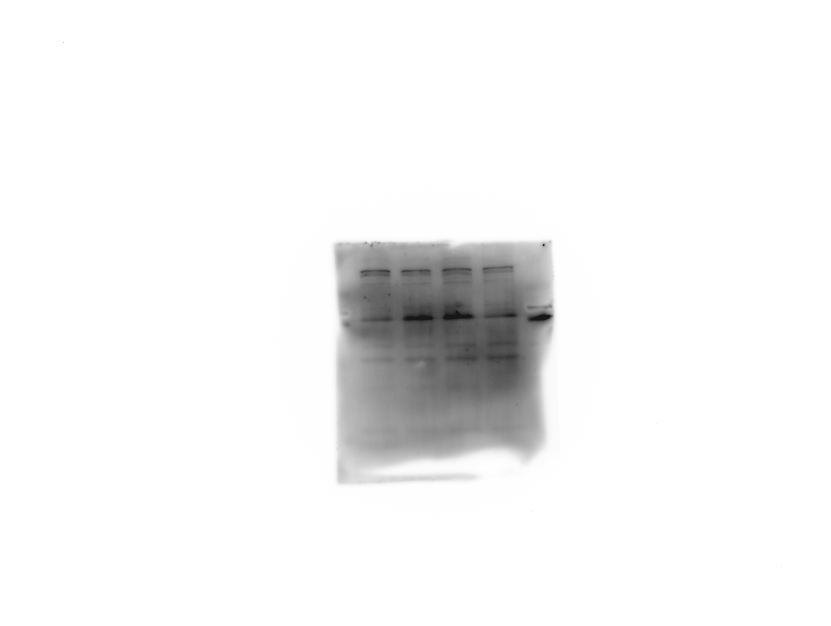

Supplement: Supplementary file 1 [file DataSheet3.ZIP › WB Original data/p-AKT/2 p-akt.png]

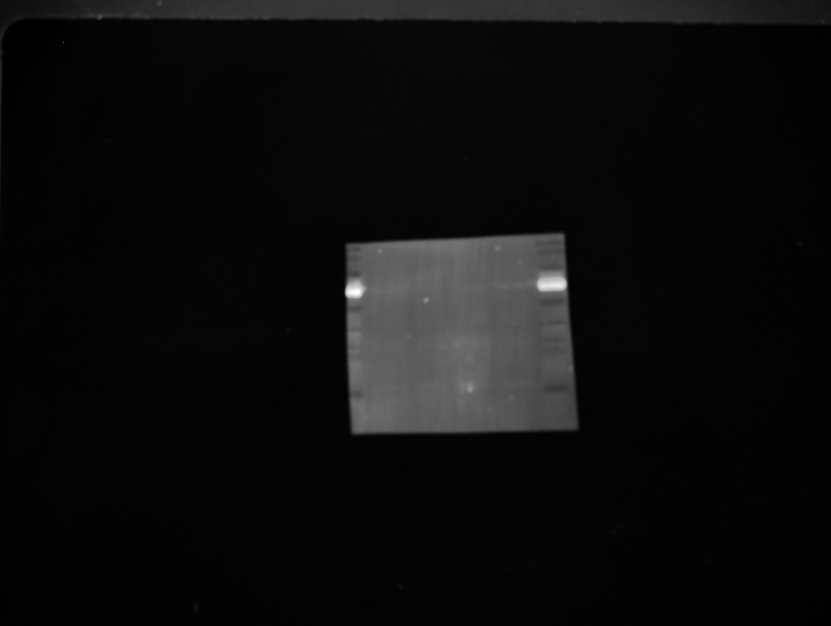

Supplement: Supplementary file 1 [file DataSheet3.ZIP › WB Original data/p-AKT/3 2023-05-09_21-52-16bit.png]

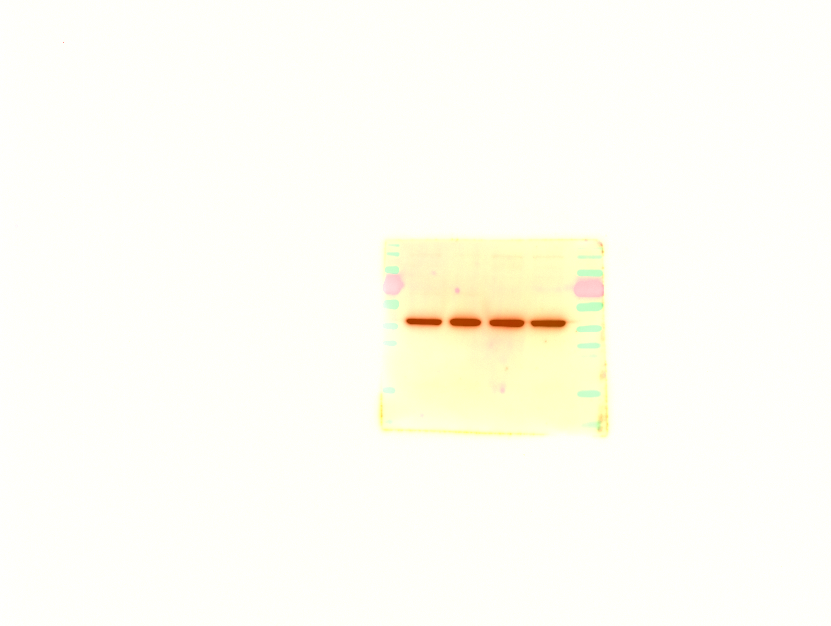

Supplement: Supplementary file 1 [file DataSheet3.ZIP › WB Original data/p-AKT/3 actin with marker.png]

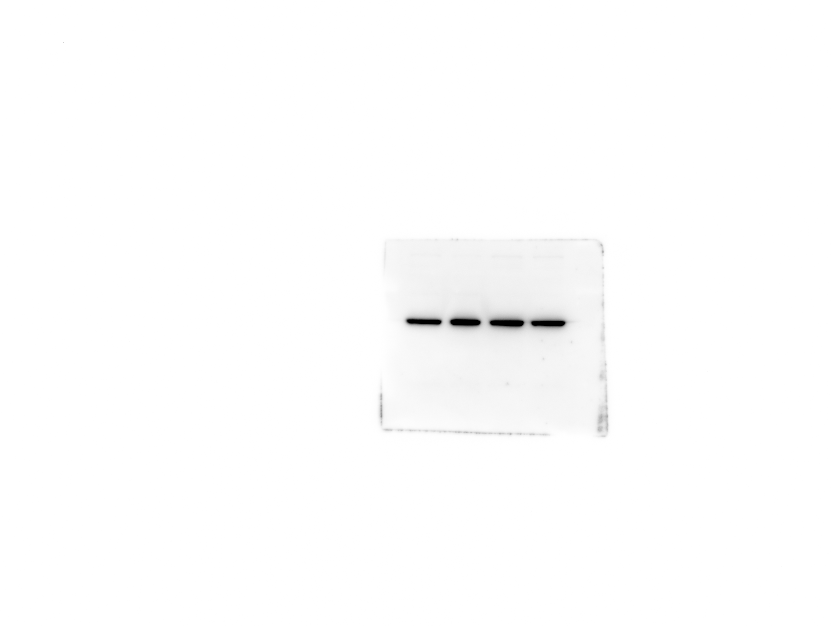

Supplement: Supplementary file 1 [file DataSheet3.ZIP › WB Original data/p-AKT/3 actin.png]

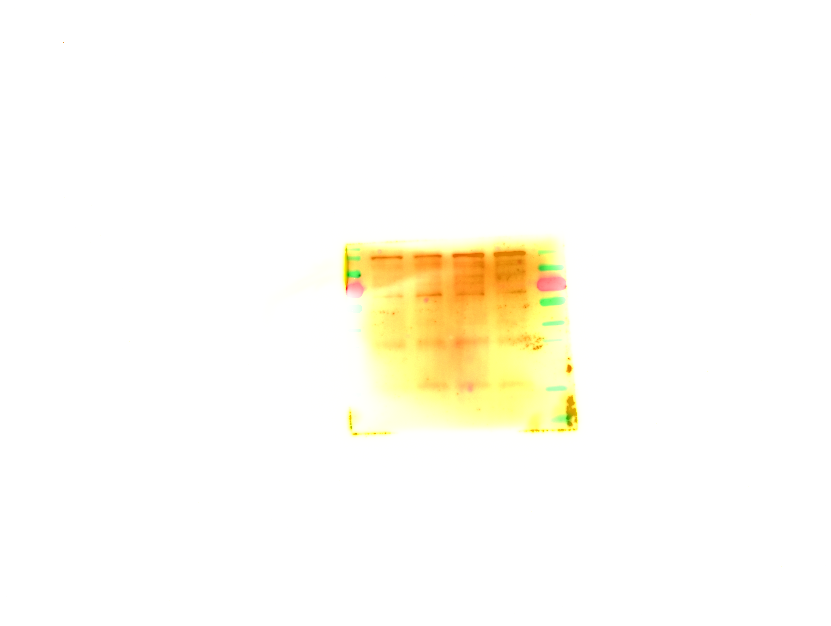

Supplement: Supplementary file 1 [file DataSheet3.ZIP › WB Original data/p-AKT/3 p-akt with marker.png]

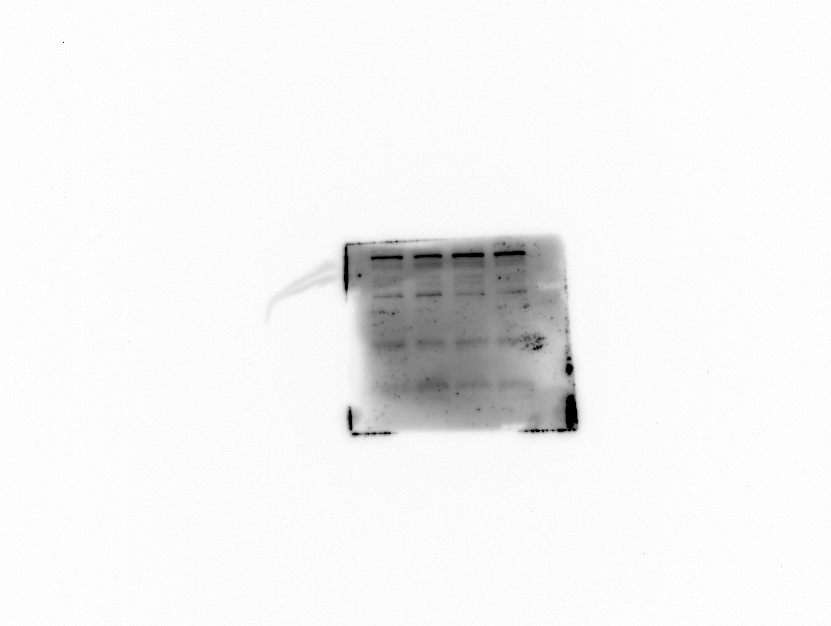

Supplement: Supplementary file 1 [file DataSheet3.ZIP › WB Original data/p-AKT/3 p-akt.png]

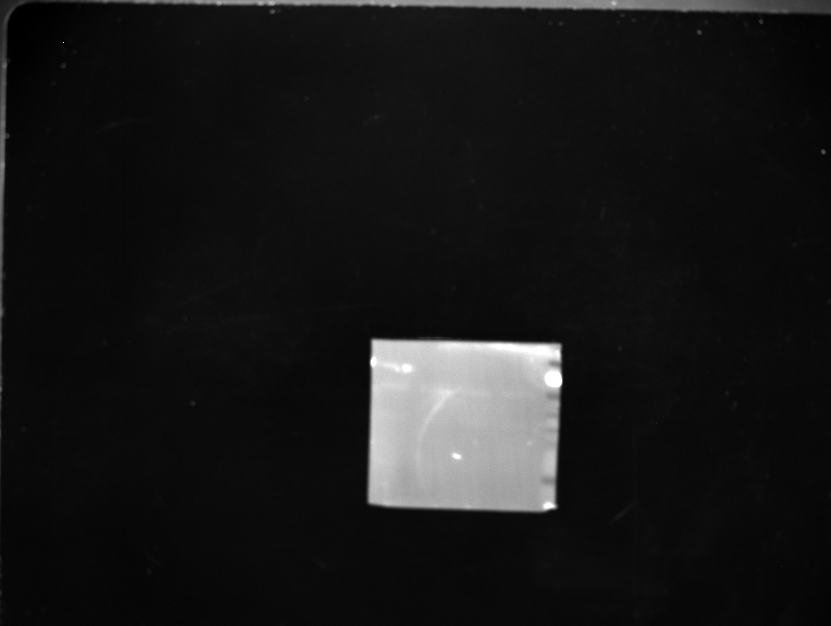

Supplement: Supplementary file 1 [file DataSheet3.ZIP › WB Original data/P-JNK/1 2023-05-19_ 1_3_16bit.png]

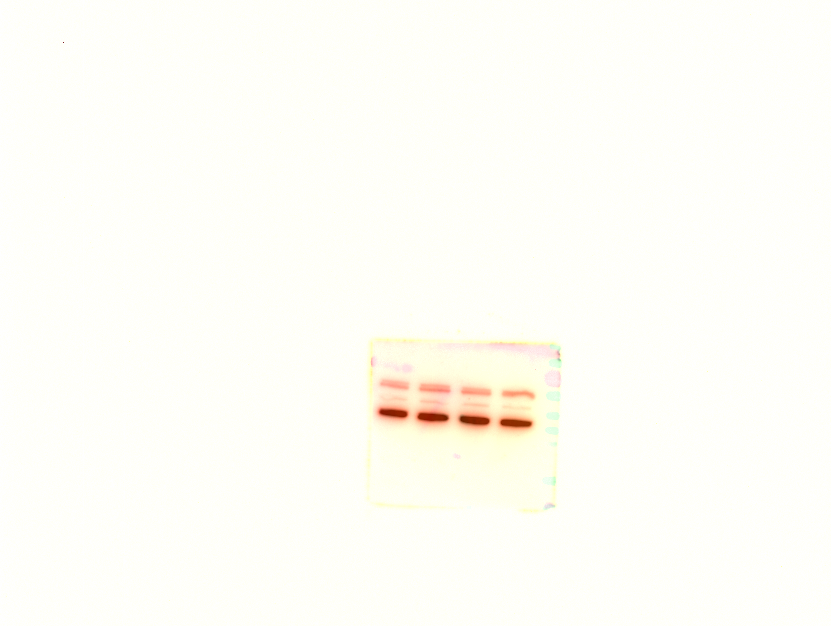

Supplement: Supplementary file 1 [file DataSheet3.ZIP › WB Original data/P-JNK/1 GAPDH with marker.png]

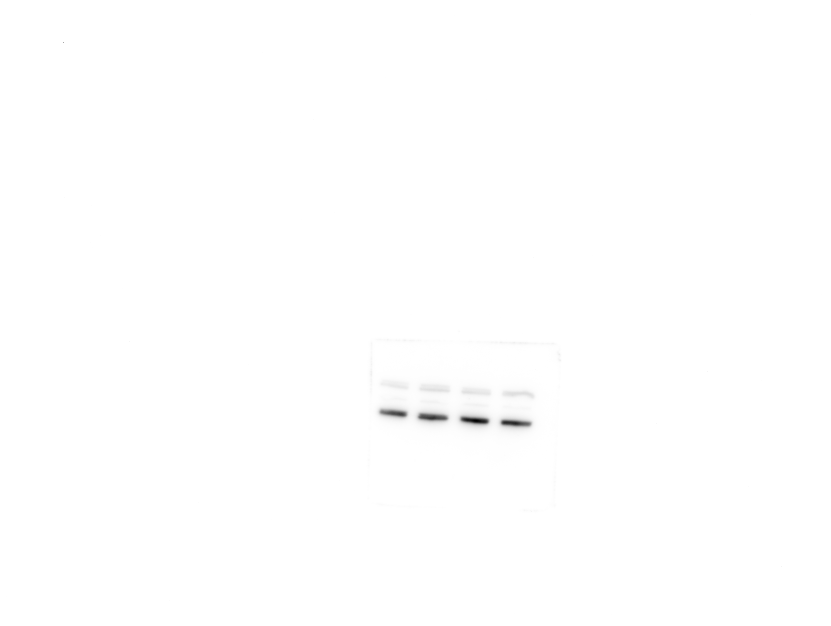

Supplement: Supplementary file 1 [file DataSheet3.ZIP › WB Original data/P-JNK/1 GAPDH.tif]

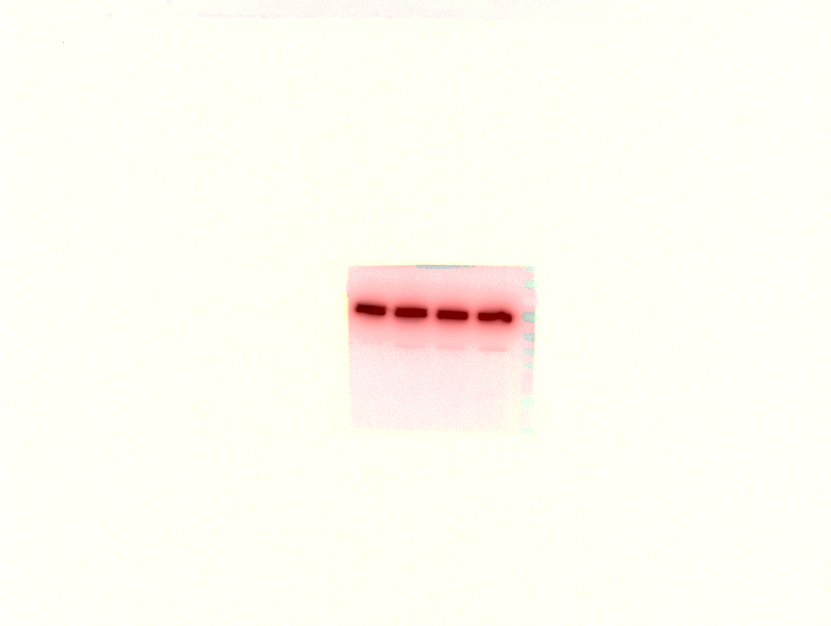

Supplement: Supplementary file 1 [file DataSheet3.ZIP › WB Original data/P-JNK/1 p-JNK with marker.png]

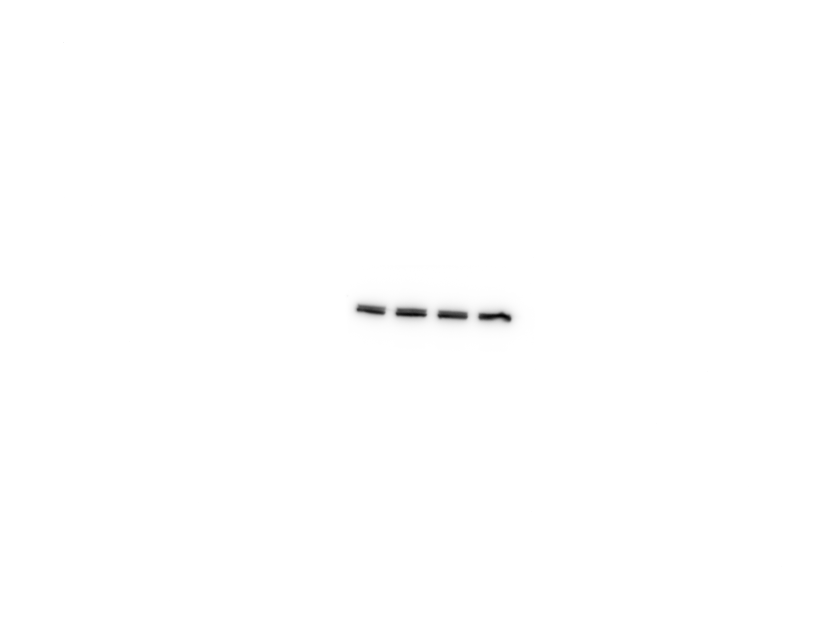

Supplement: Supplementary file 1 [file DataSheet3.ZIP › WB Original data/P-JNK/1 p-JNK.tif]

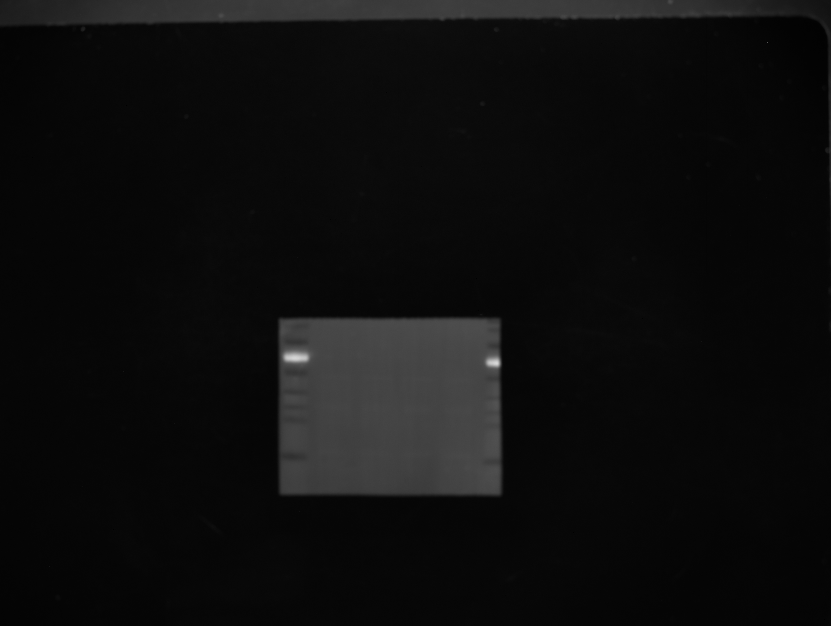

Supplement: Supplementary file 1 [file DataSheet3.ZIP › WB Original data/P-JNK/2 2023-05-19_19-08-26_3_16bit.png]

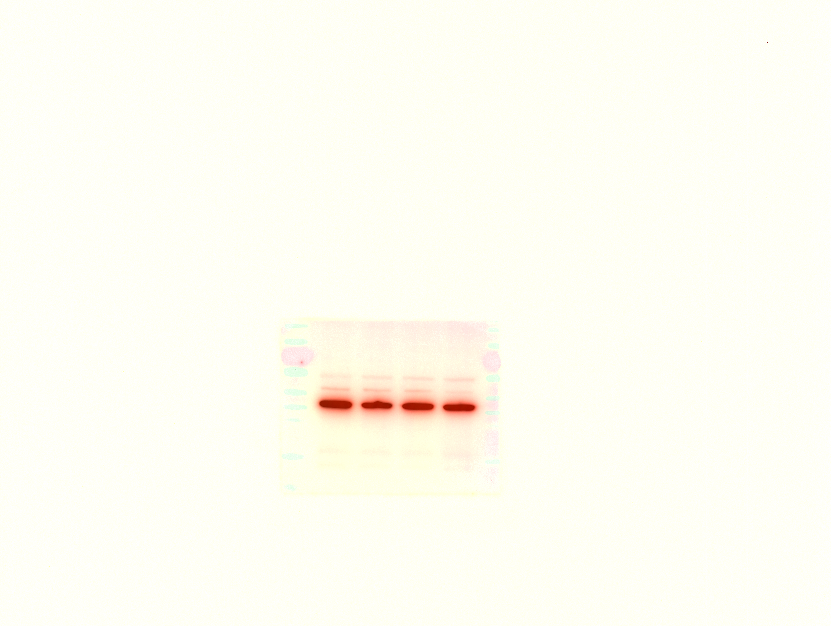

Supplement: Supplementary file 1 [file DataSheet3.ZIP › WB Original data/P-JNK/2 GAPDH with marker.png]

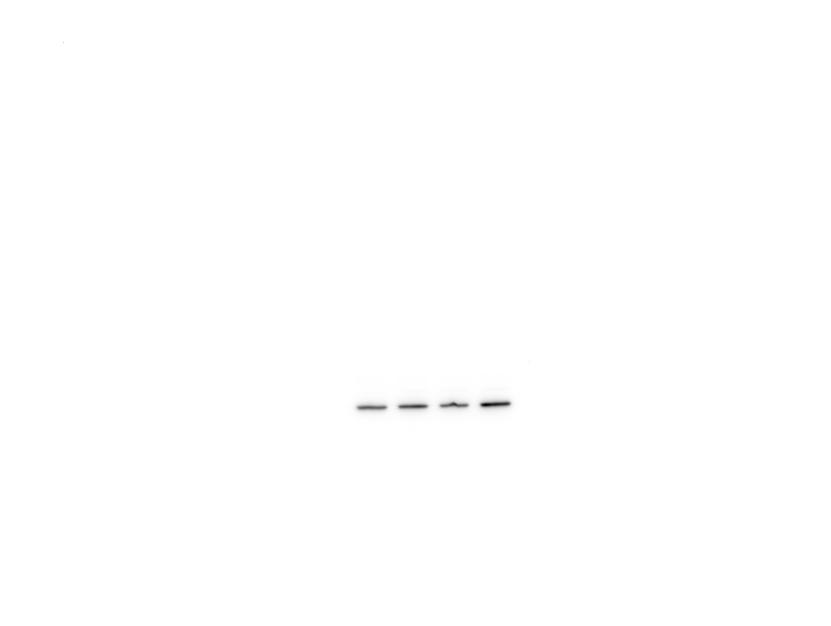

Supplement: Supplementary file 1 [file DataSheet3.ZIP › WB Original data/P-JNK/2 GAPDH.tif]

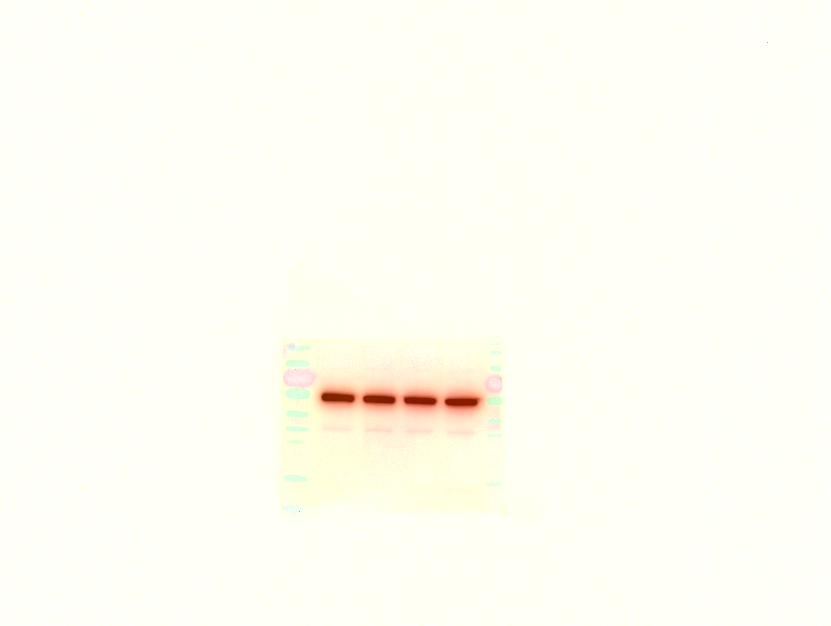

Supplement: Supplementary file 1 [file DataSheet3.ZIP › WB Original data/P-JNK/2 p-JNK with marker.png]

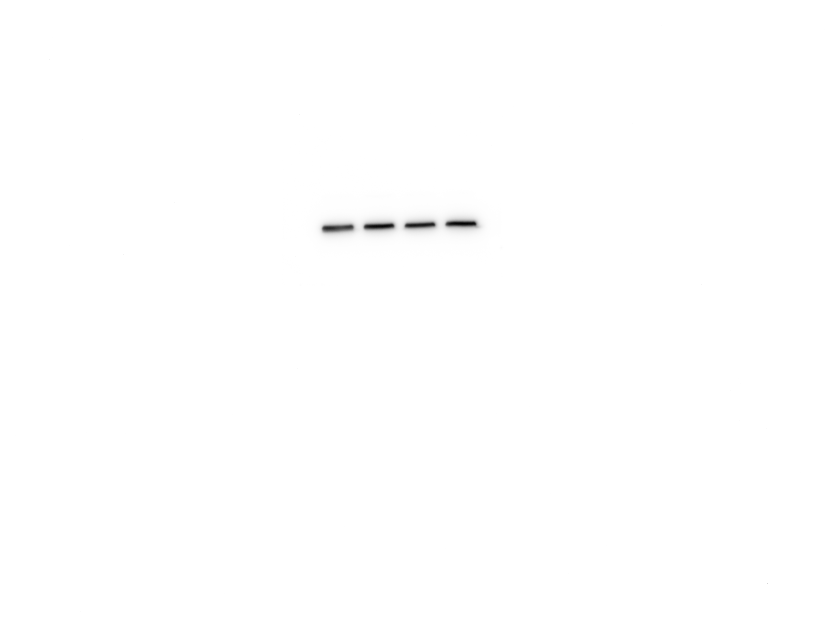

Supplement: Supplementary file 1 [file DataSheet3.ZIP › WB Original data/P-JNK/2 p-JNK.tif]

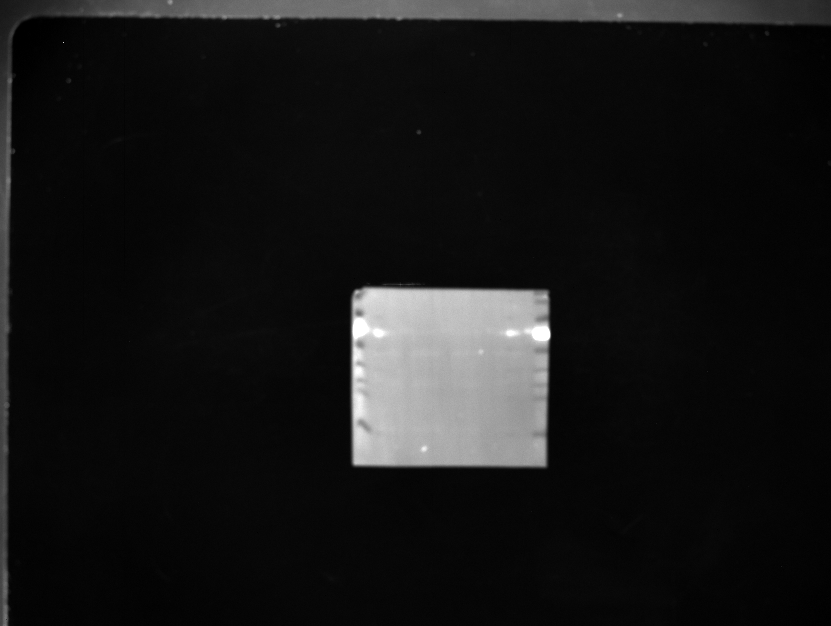

Supplement: Supplementary file 1 [file DataSheet3.ZIP › WB Original data/P-JNK/3 2023-05-19 3_3_16bit.png]

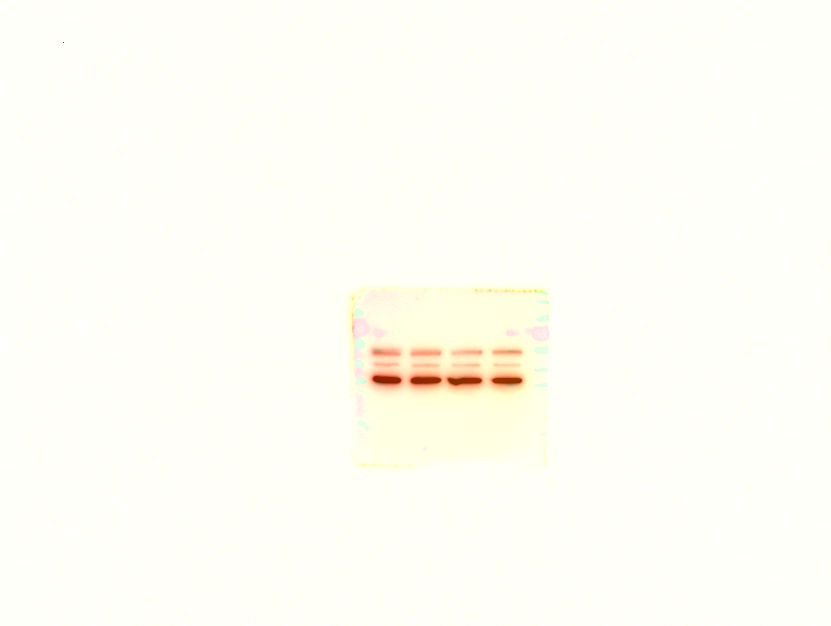

Supplement: Supplementary file 1 [file DataSheet3.ZIP › WB Original data/P-JNK/3 GAPDH with marker.png]

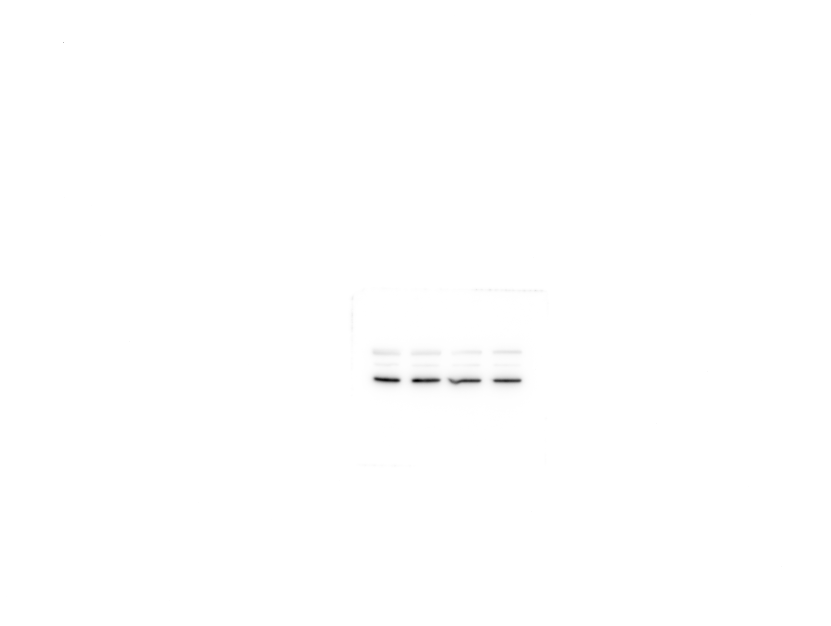

Supplement: Supplementary file 1 [file DataSheet3.ZIP › WB Original data/P-JNK/3 GAPDH.tif]

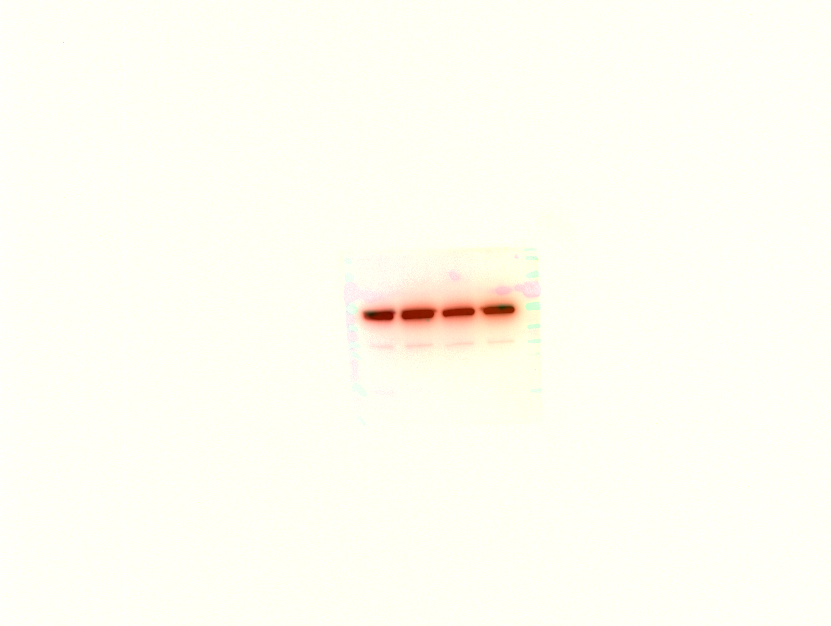

Supplement: Supplementary file 1 [file DataSheet3.ZIP › WB Original data/P-JNK/3 p-JNK with marker.png]

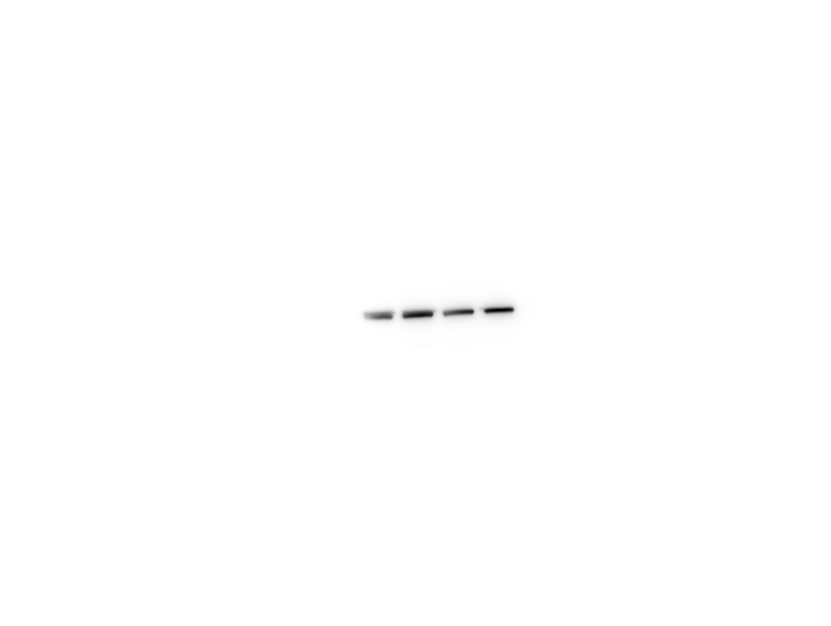

Supplement: Supplementary file 1 [file DataSheet3.ZIP › WB Original data/P-JNK/3 p-JNK.tif]

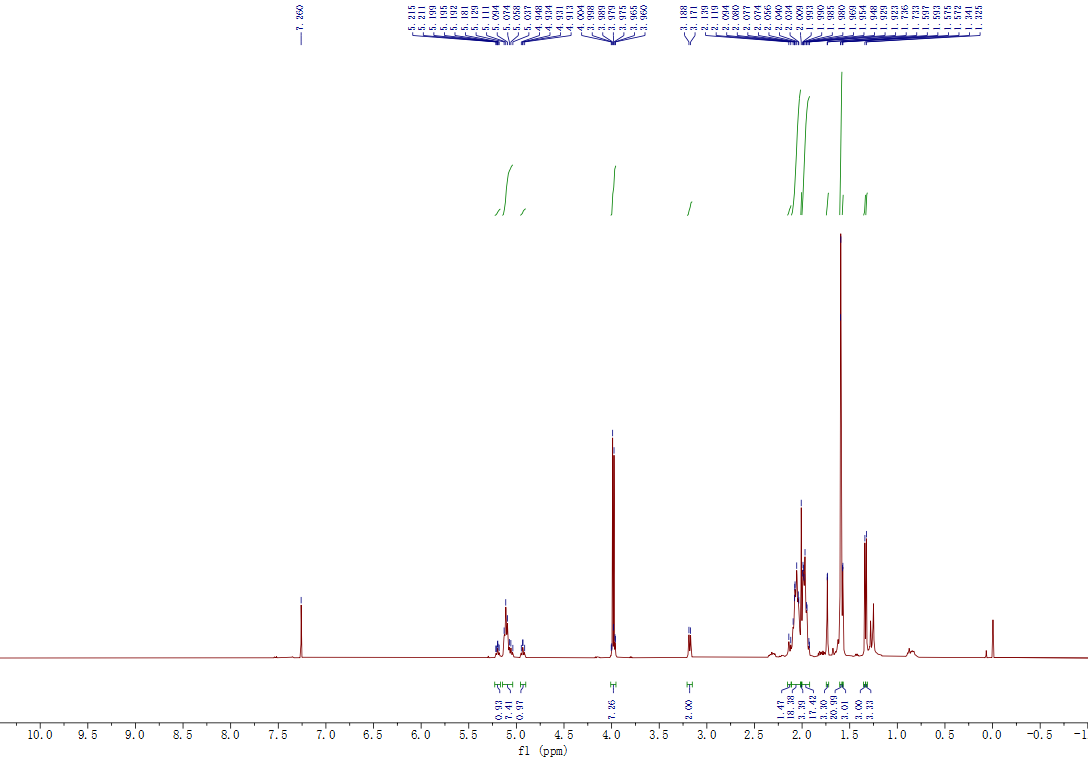


1H NMR of L-50 (CDCl3, 400 MHz)


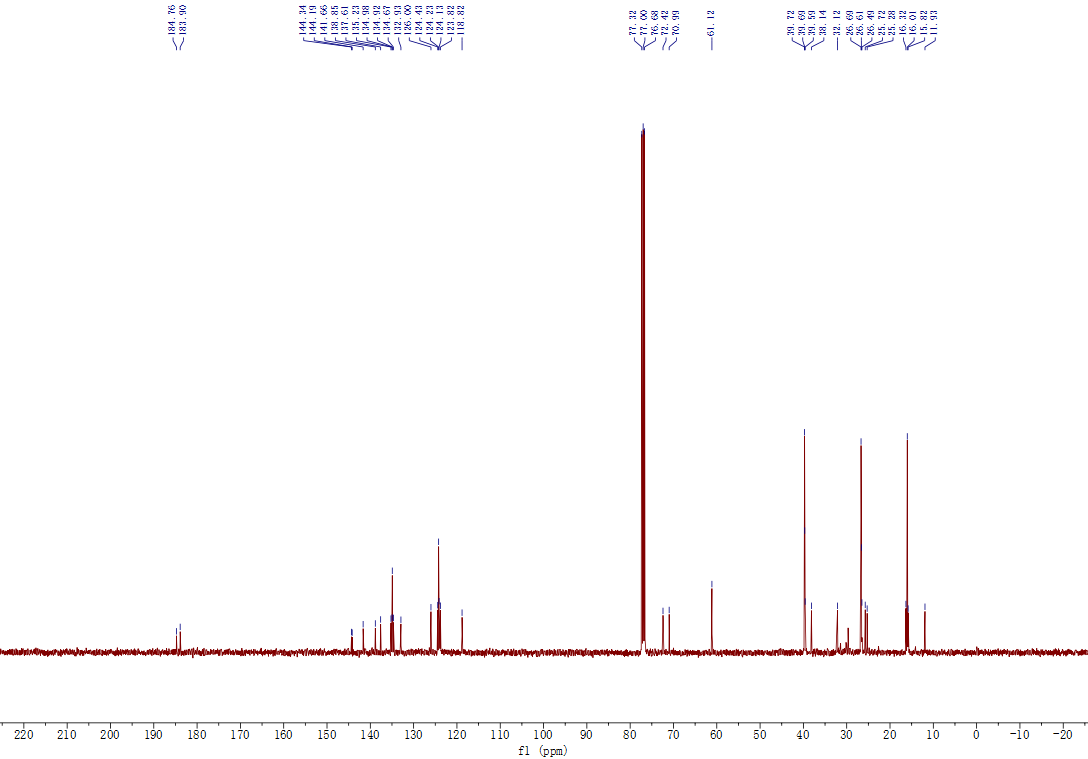


13C NMR of L-50 (CDCl3, 100 MHz)

Supplement: Supplementary file 2 [file DataSheet1.ZIP › 相关源数据/NMR谱图.docx]

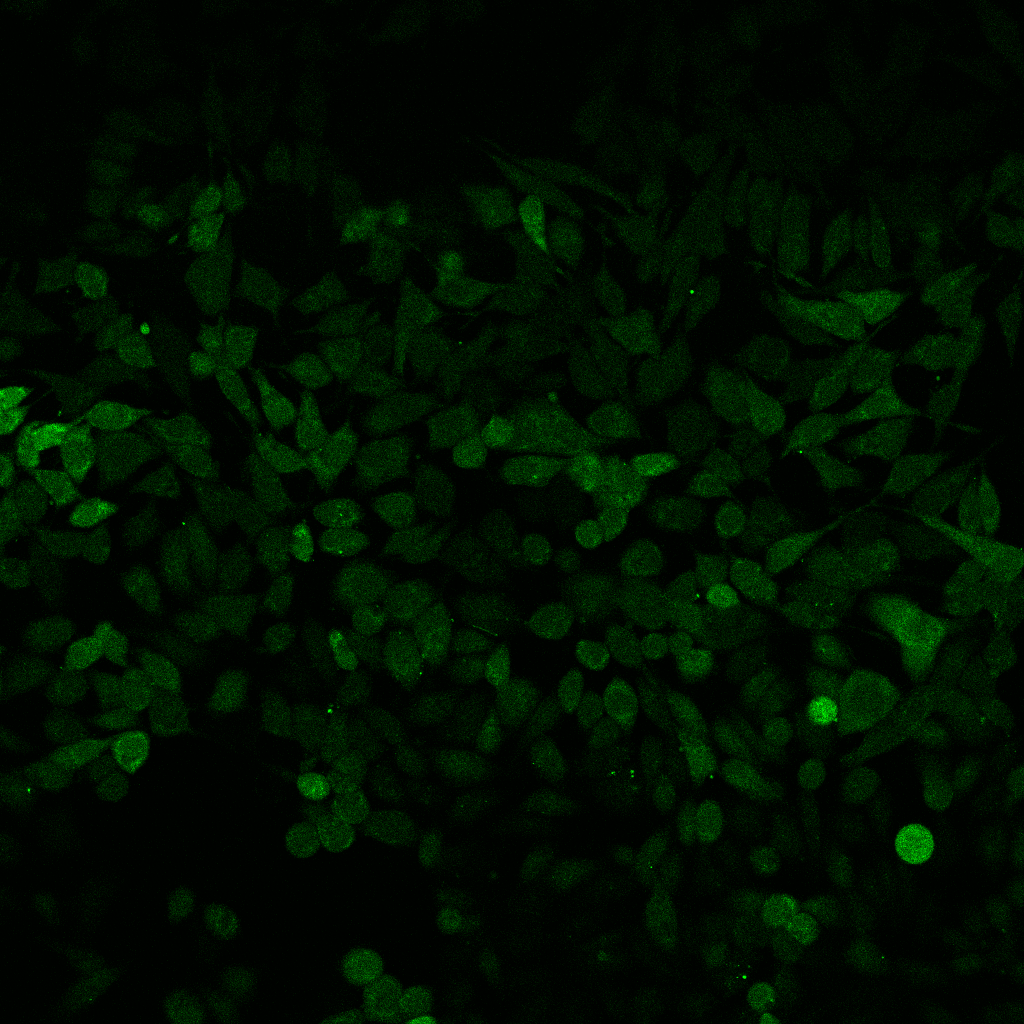

Supplement: Supplementary file 3 [file DataSheet2.ZIP › ROS Original data/100μML-50 (1).tif]

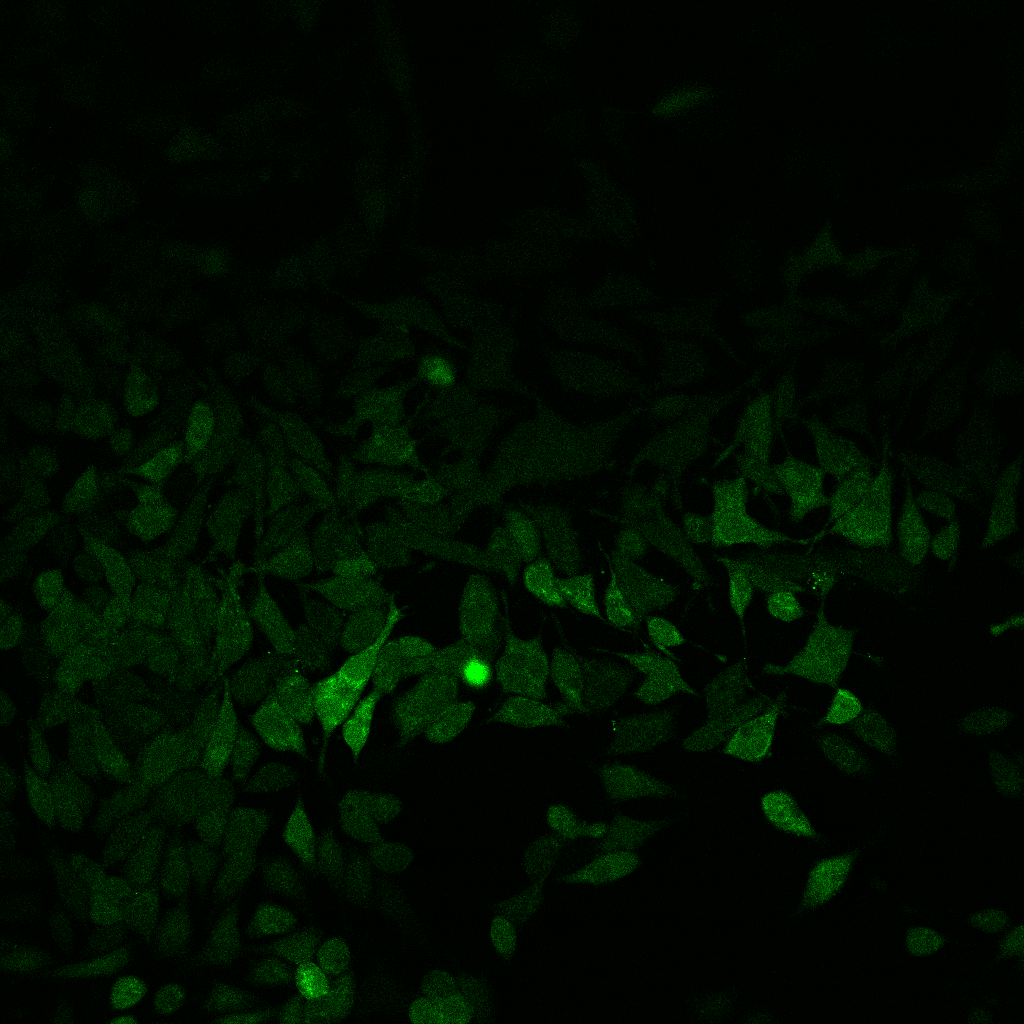

Supplement: Supplementary file 3 [file DataSheet2.ZIP › ROS Original data/100μML-50 (2).tif]

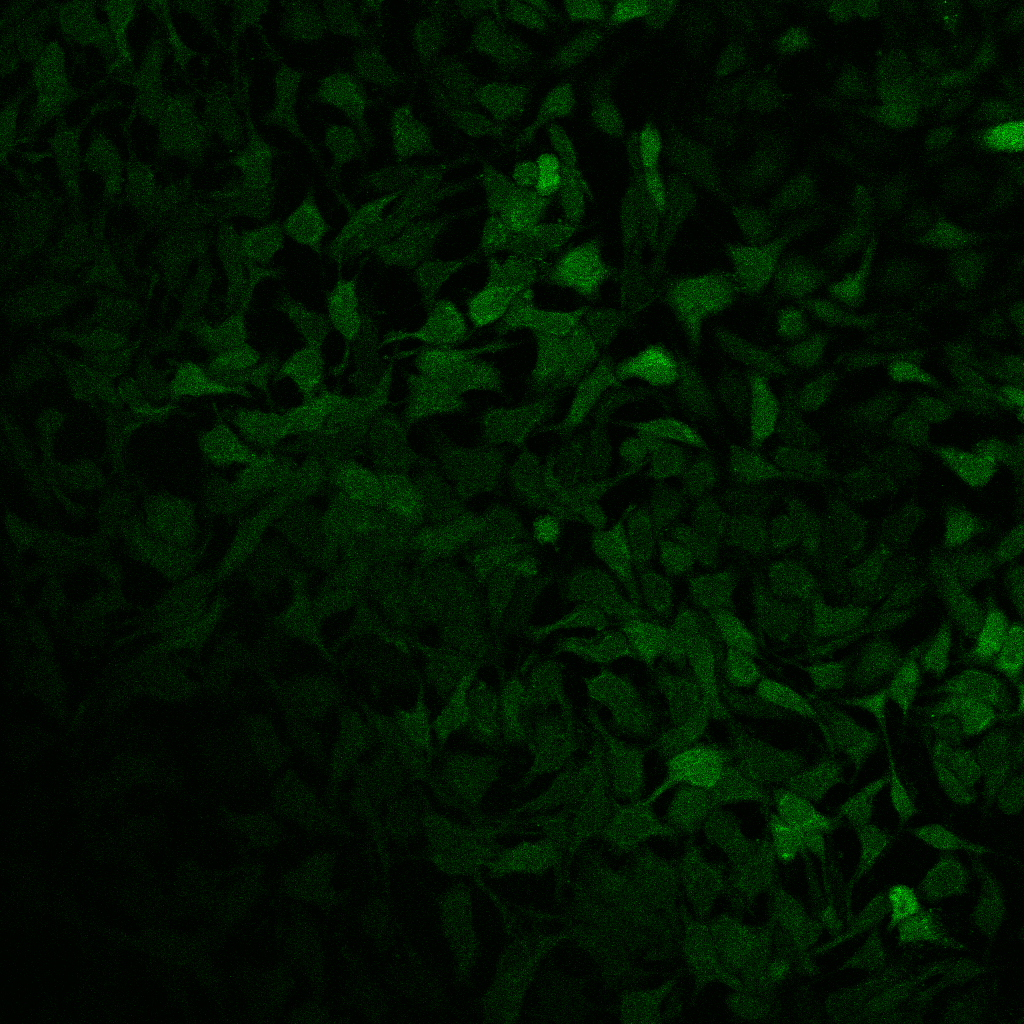

Supplement: Supplementary file 3 [file DataSheet2.ZIP › ROS Original data/100μML-50 (3).tif]

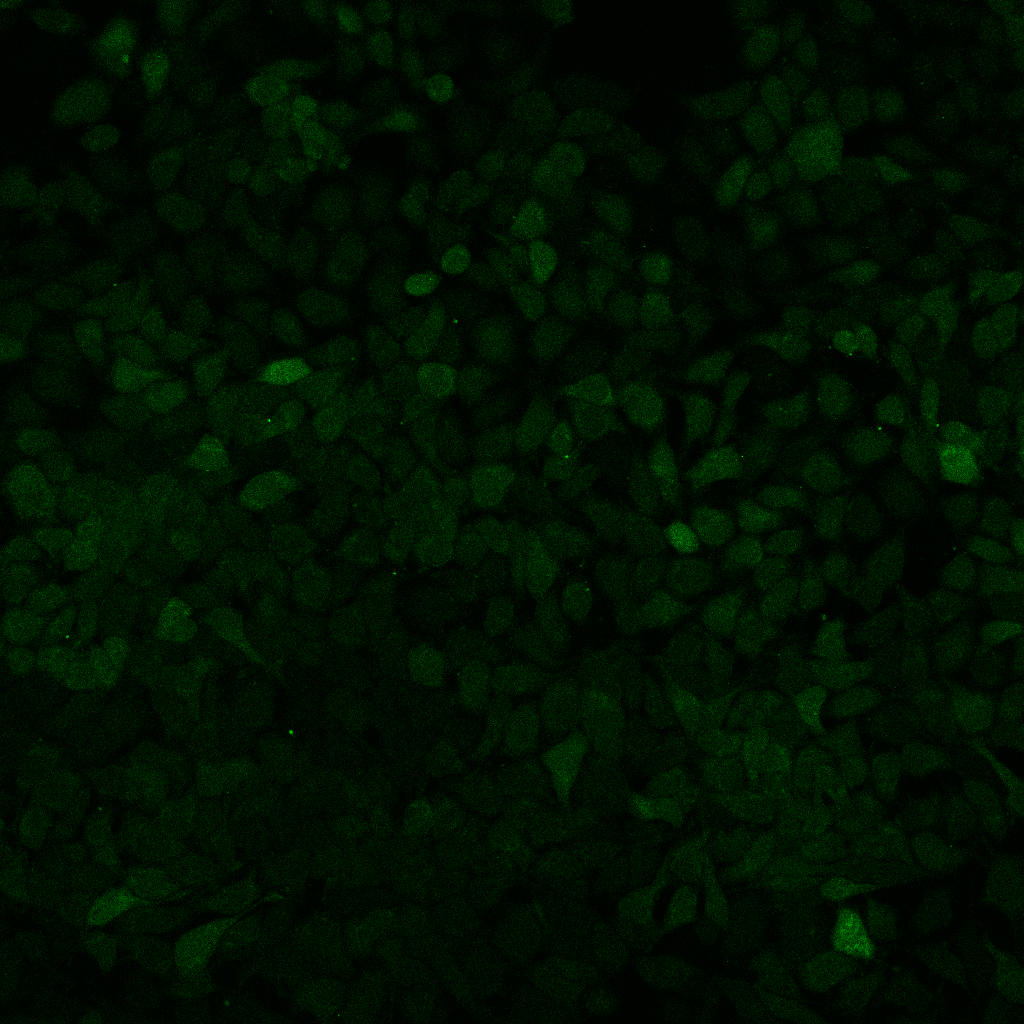

Supplement: Supplementary file 3 [file DataSheet2.ZIP › ROS Original data/10μML-50 (1).tif]

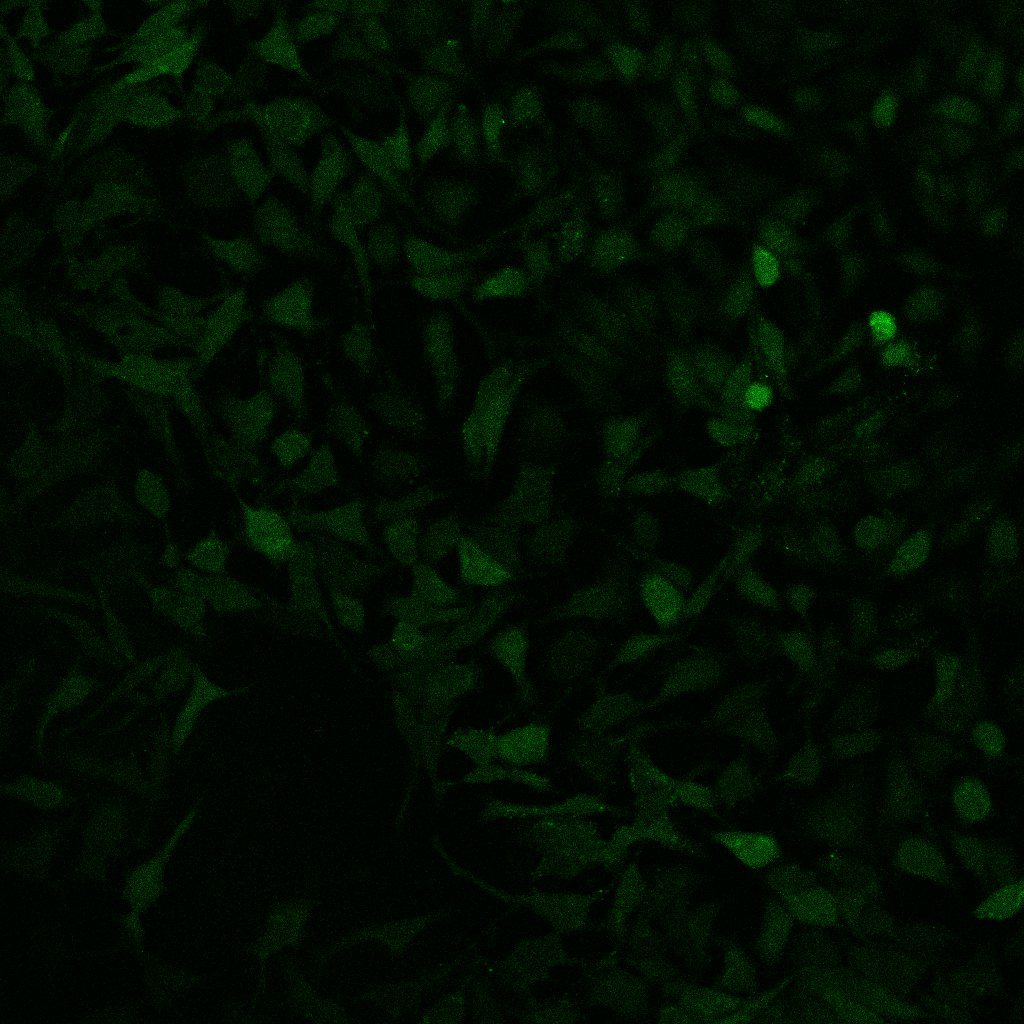

Supplement: Supplementary file 3 [file DataSheet2.ZIP › ROS Original data/10μML-50 (2).tif]

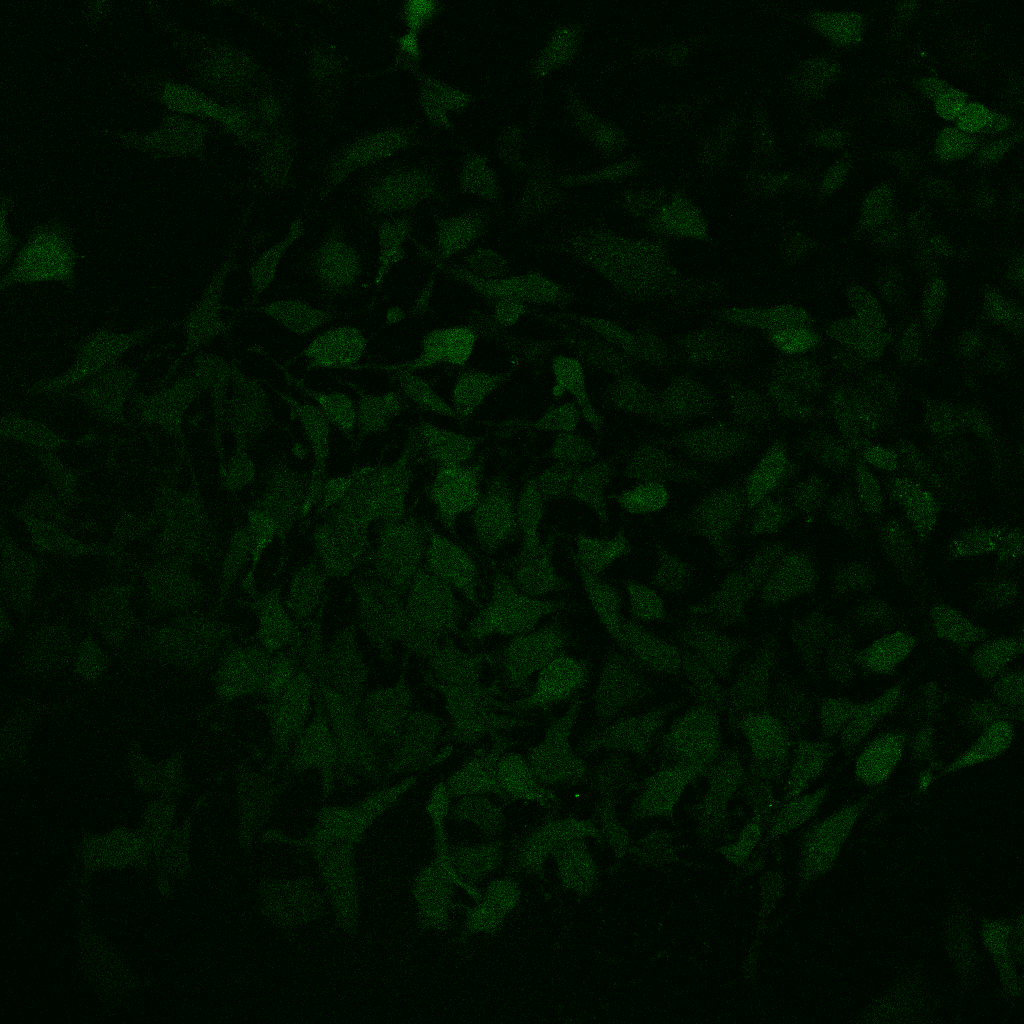

Supplement: Supplementary file 3 [file DataSheet2.ZIP › ROS Original data/10μML-50 (3).tif]

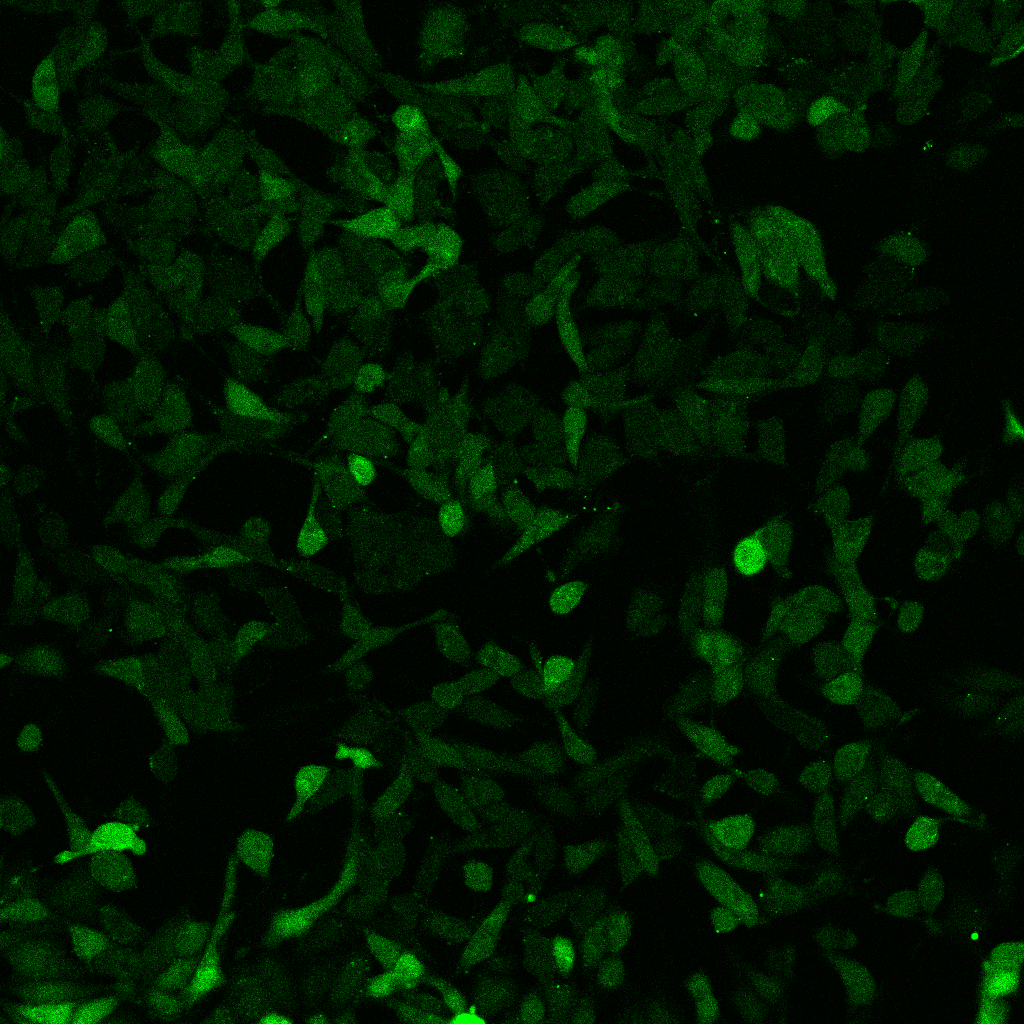

Supplement: Supplementary file 3 [file DataSheet2.ZIP › ROS Original data/1μML-50 (1).tif]

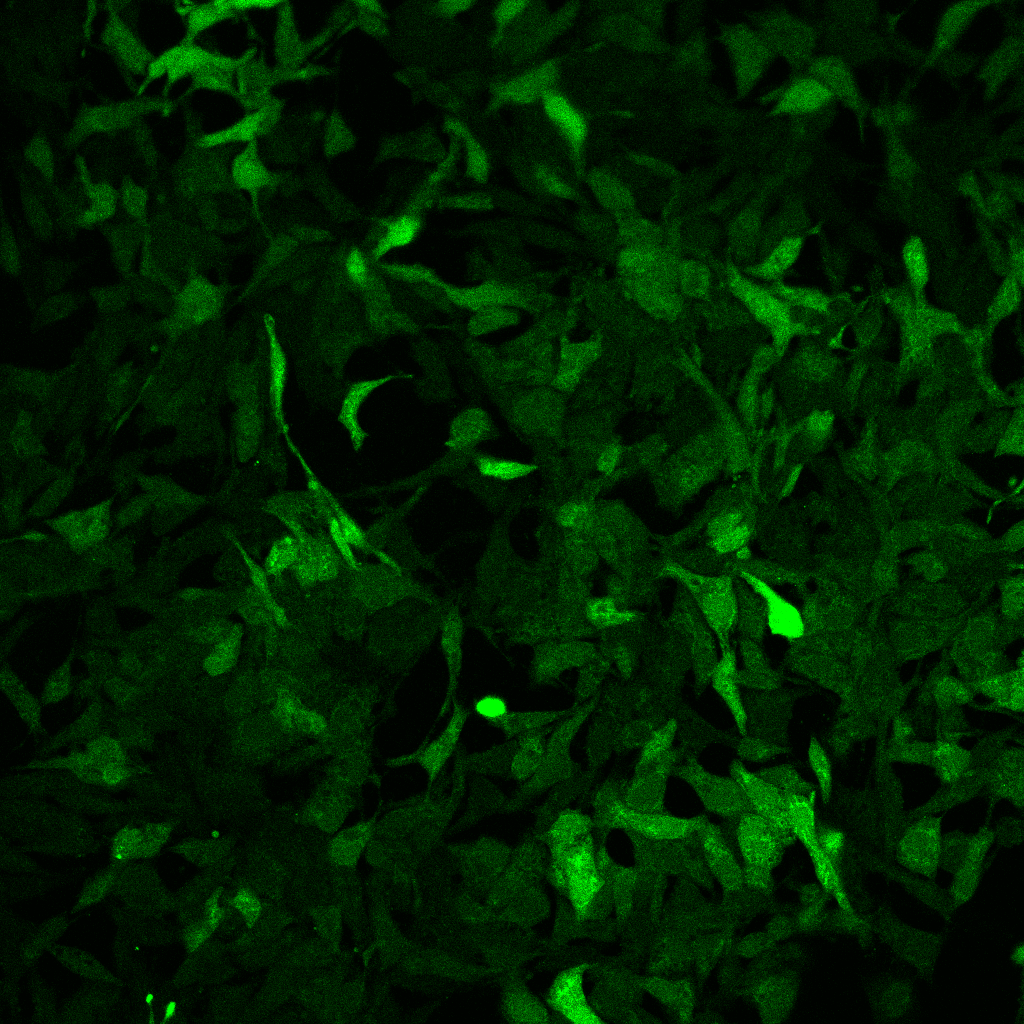

Supplement: Supplementary file 3 [file DataSheet2.ZIP › ROS Original data/1μML-50 (2).tif]

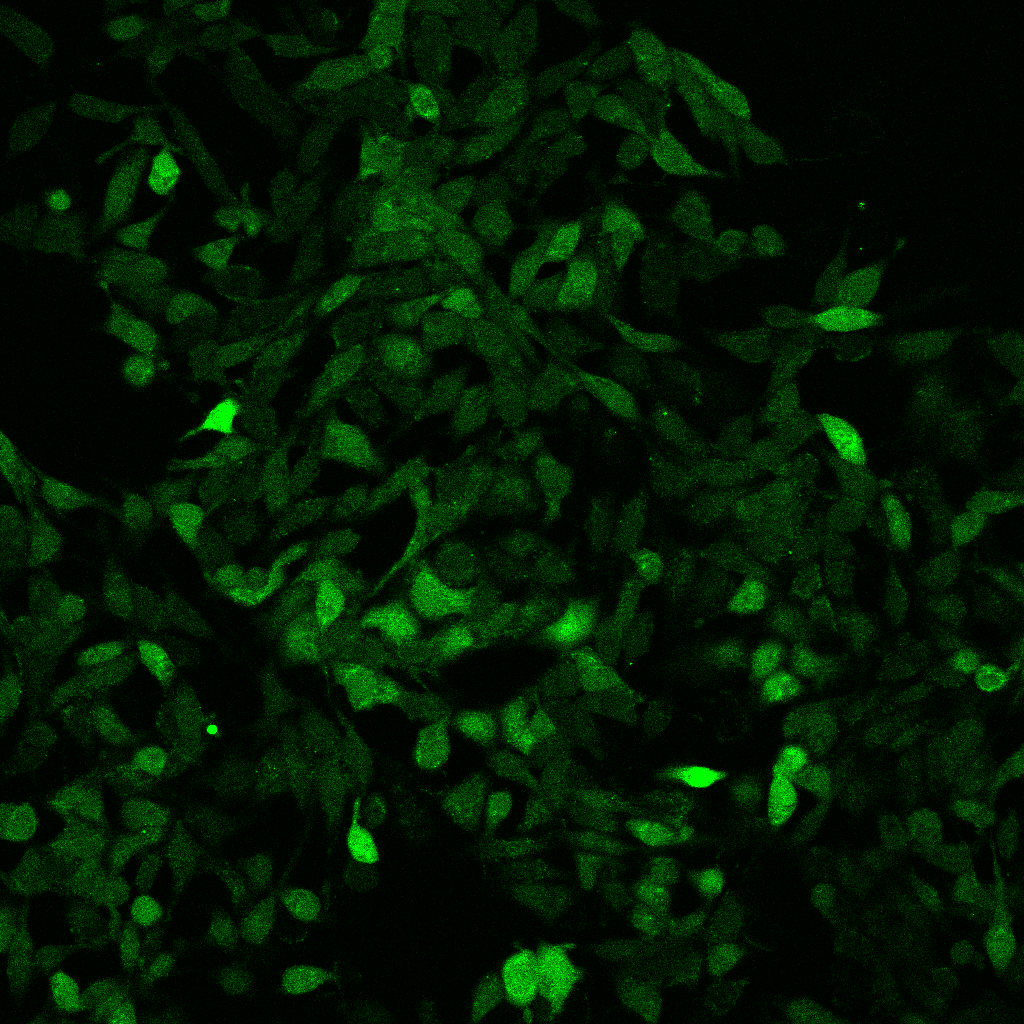

Supplement: Supplementary file 3 [file DataSheet2.ZIP › ROS Original data/1μML-50 (3).tif]

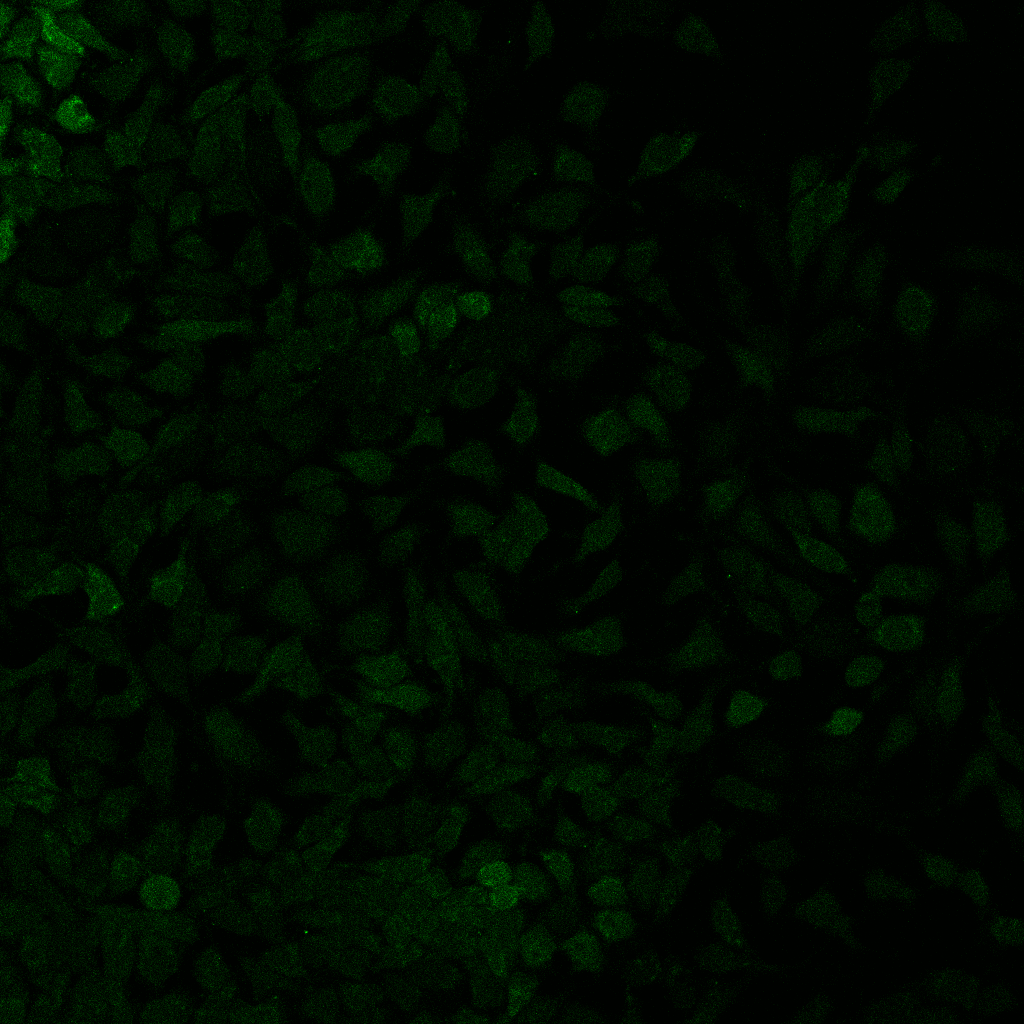

Supplement: Supplementary file 3 [file DataSheet2.ZIP › ROS Original data/Control (1).tif]

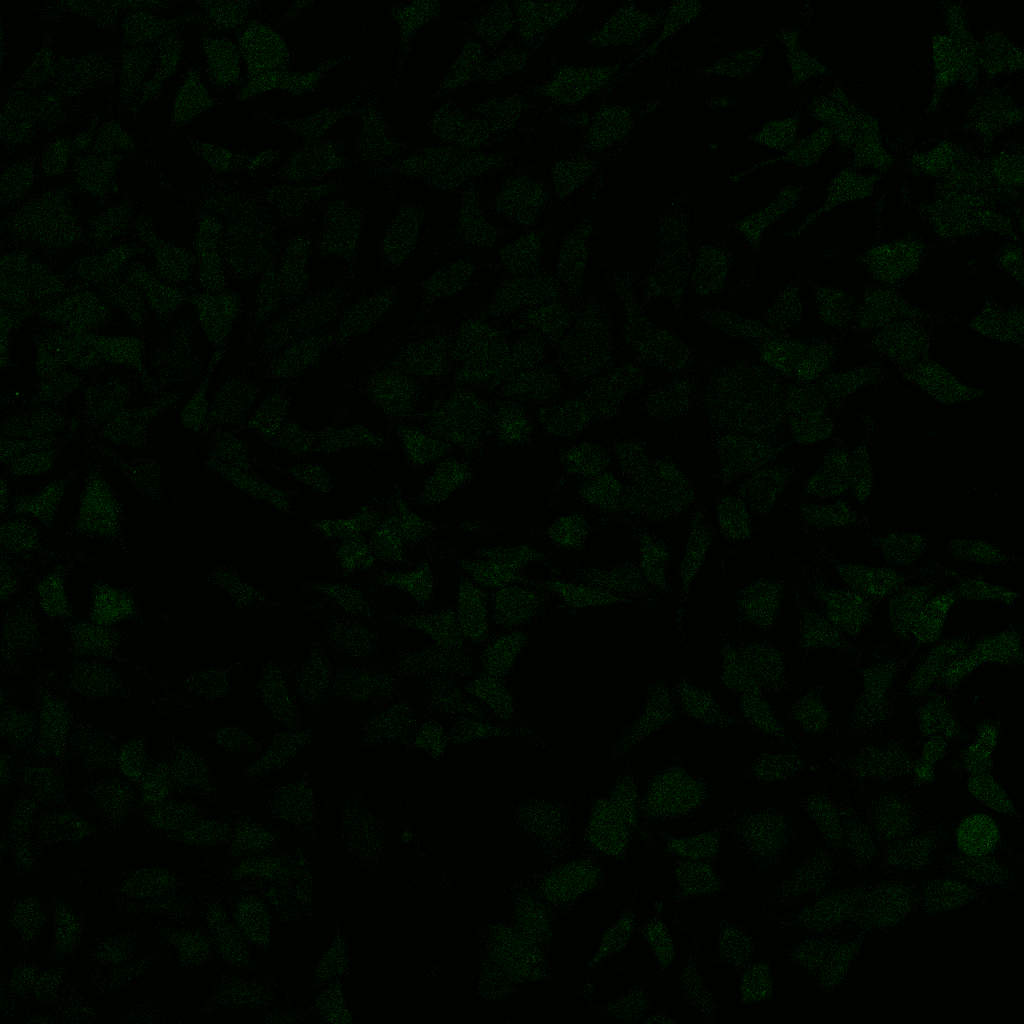

Supplement: Supplementary file 3 [file DataSheet2.ZIP › ROS Original data/Control (2).tif]

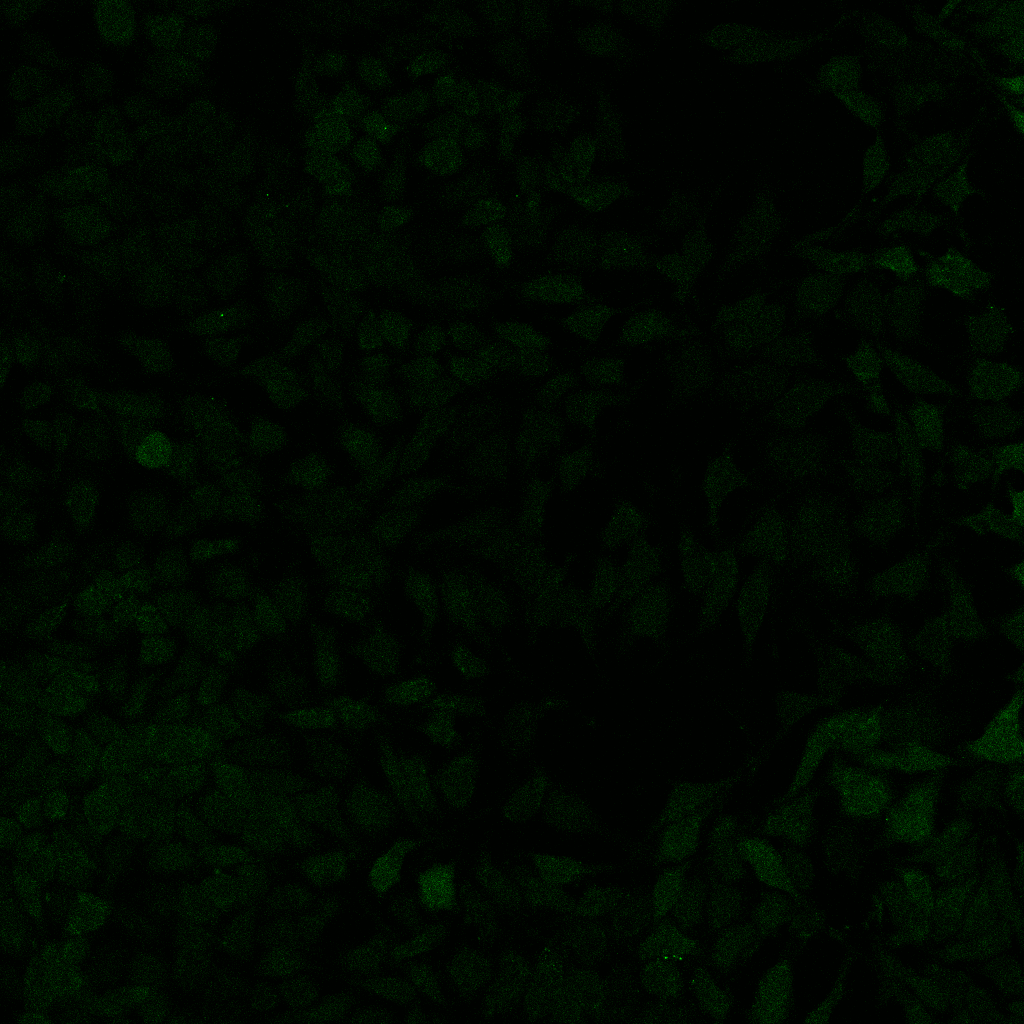

Supplement: Supplementary file 3 [file DataSheet2.ZIP › ROS Original data/Control (3).tif]

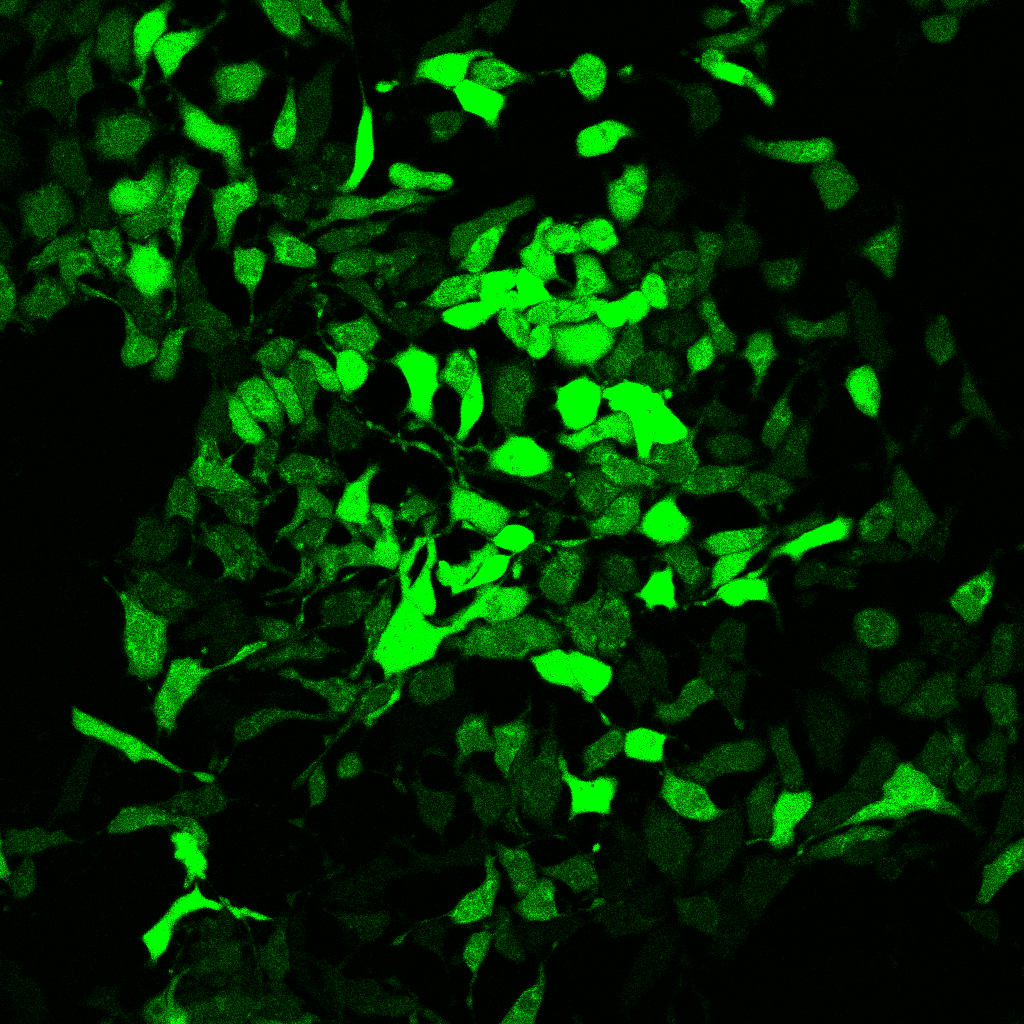

Supplement: Supplementary file 3 [file DataSheet2.ZIP › ROS Original data/Model (1).tif]

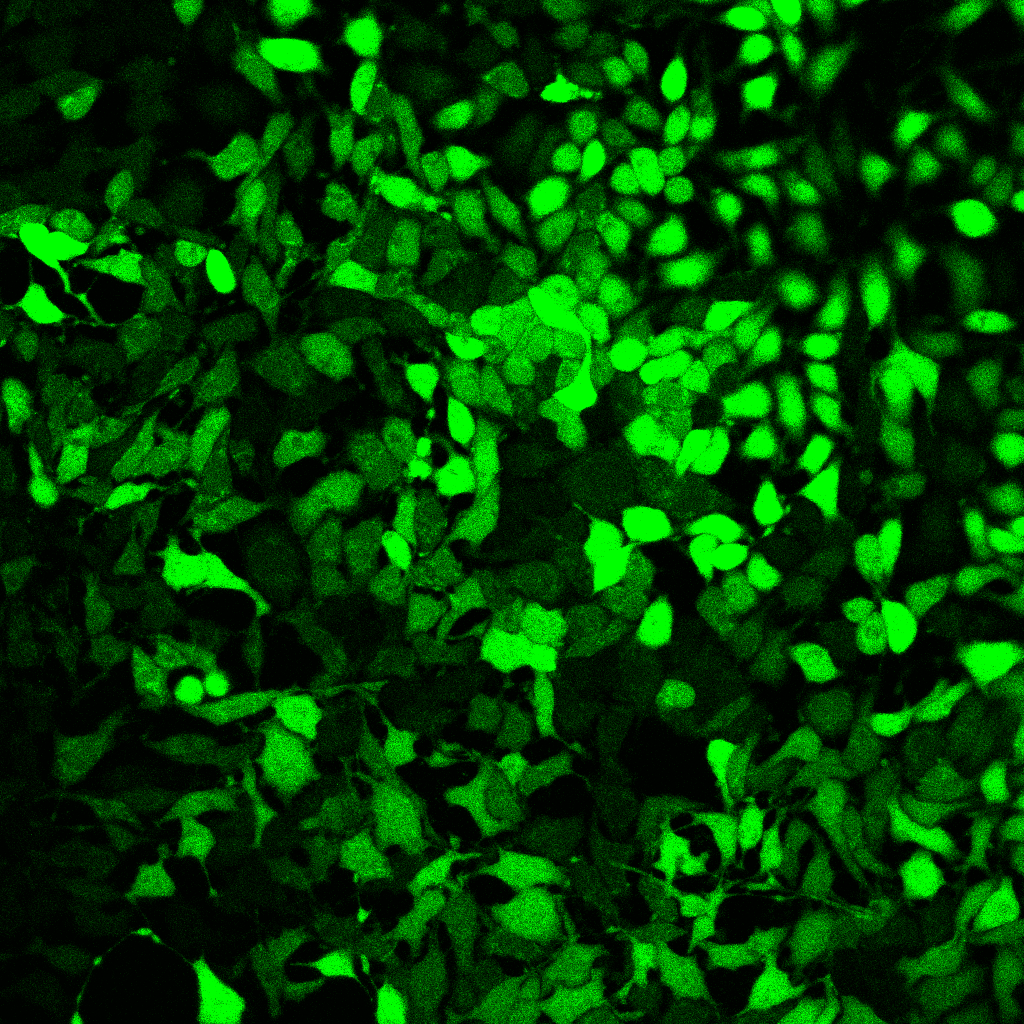

Supplement: Supplementary file 3 [file DataSheet2.ZIP › ROS Original data/Model (2).tif]

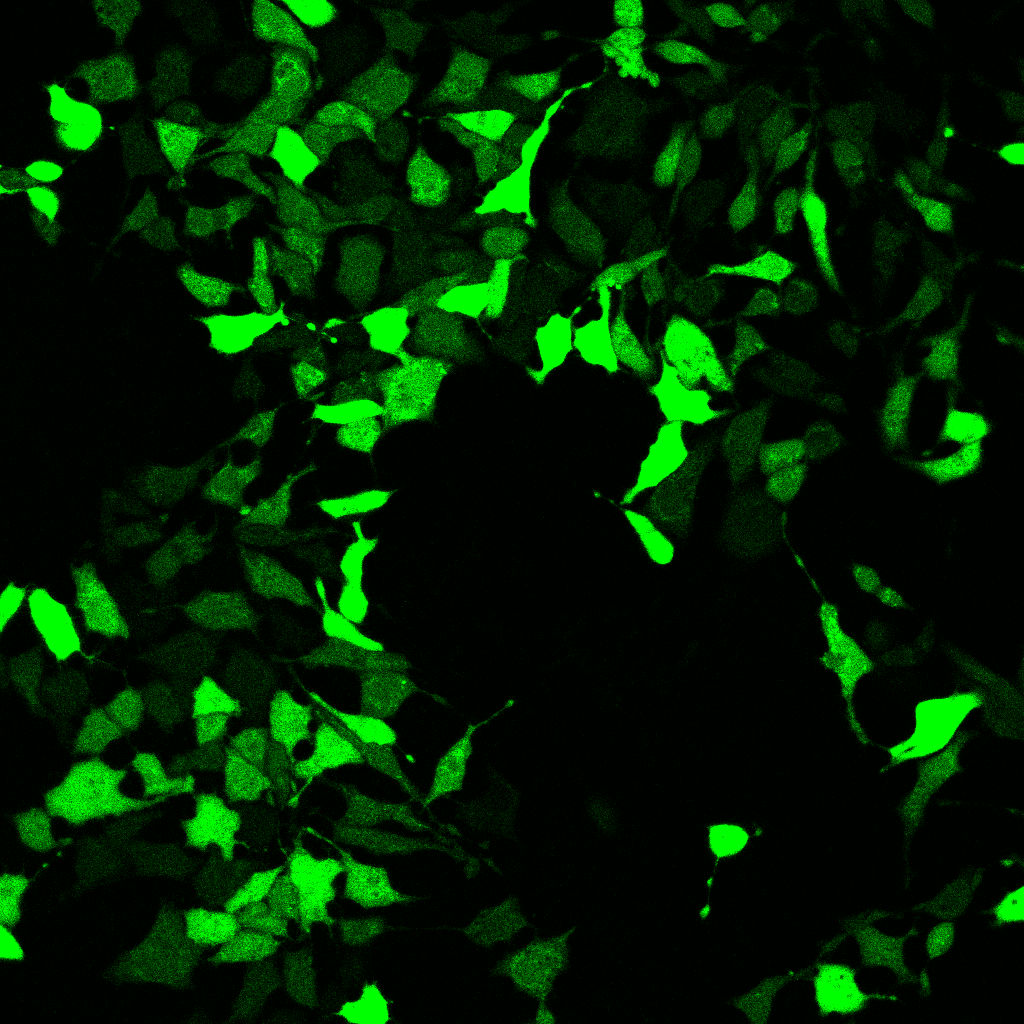

Supplement: Supplementary file 3 [file DataSheet2.ZIP › ROS Original data/Model (3).tif]

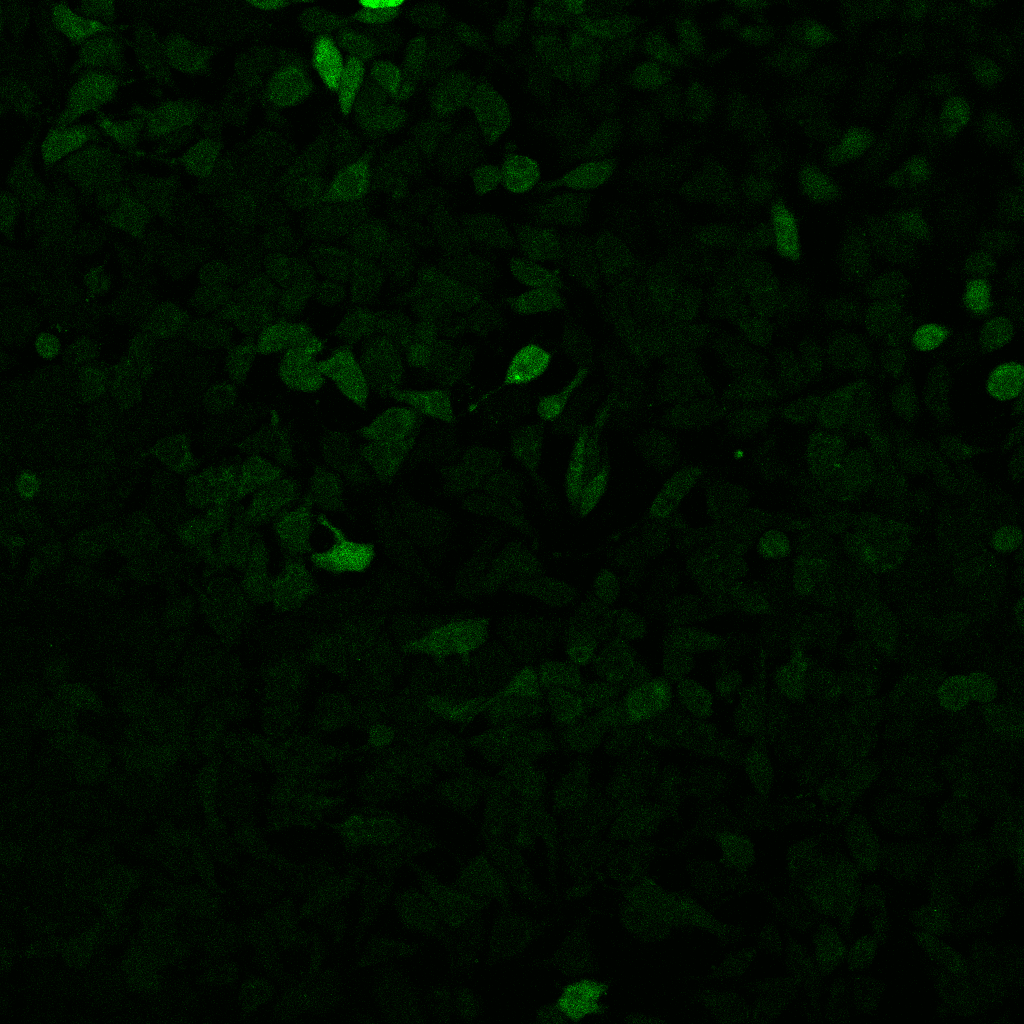

Supplement: Supplementary file 3 [file DataSheet2.ZIP › ROS Original data/Rosi (1).tif]

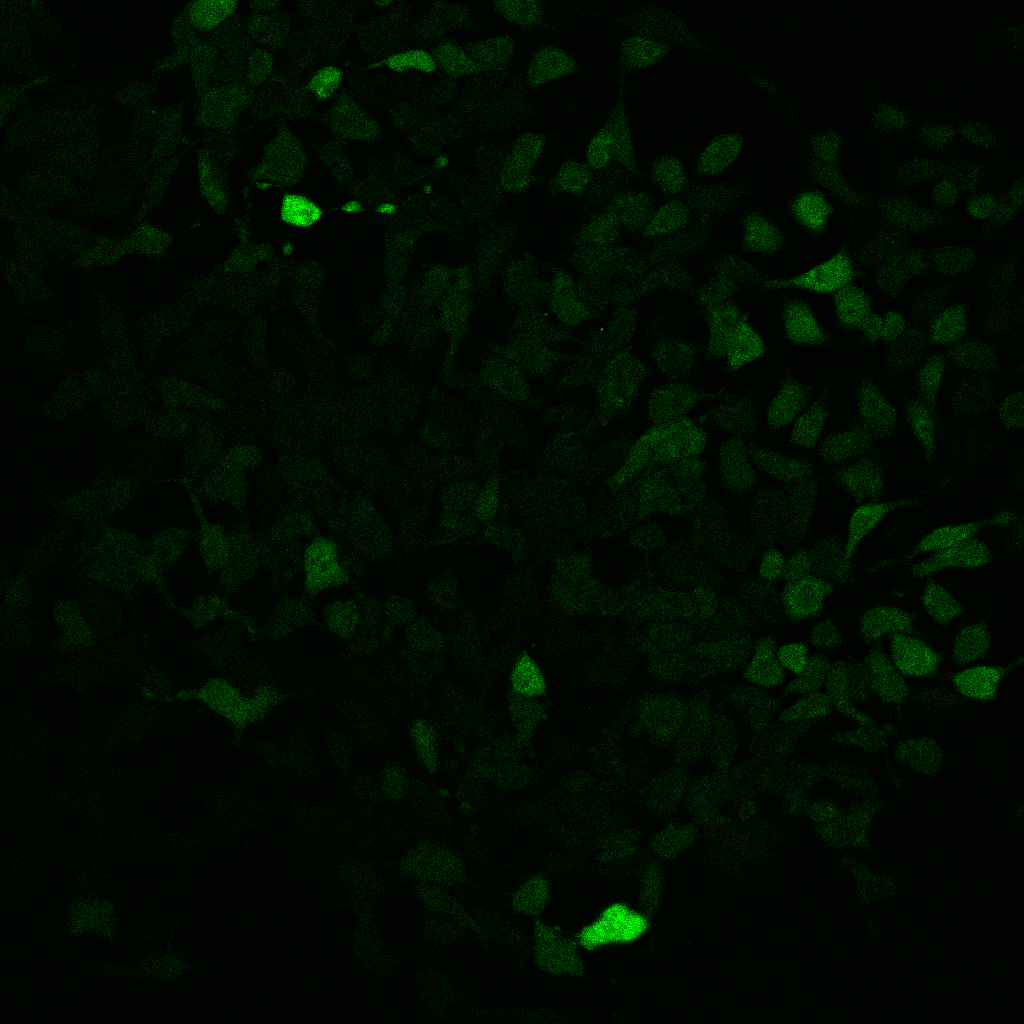

Supplement: Supplementary file 3 [file DataSheet2.ZIP › ROS Original data/Rosi (2).tif]

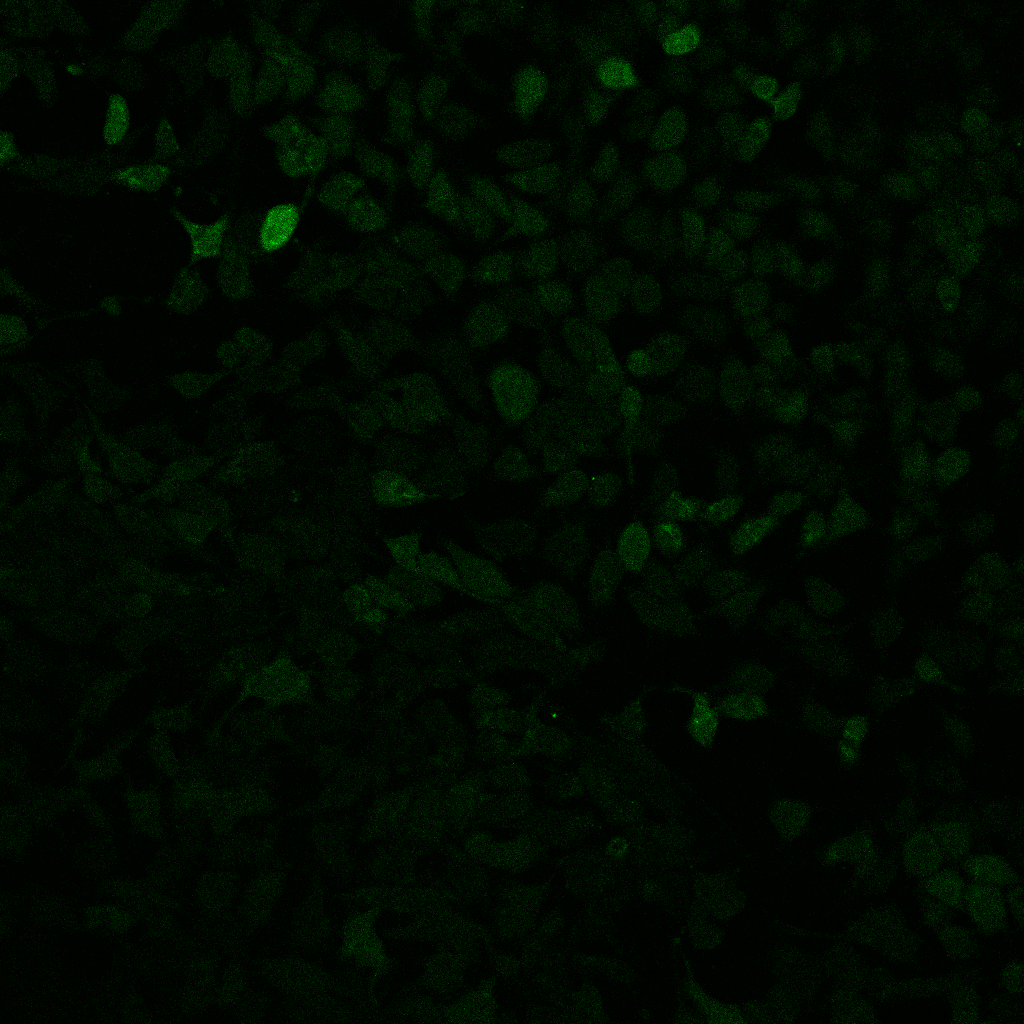

Supplement: Supplementary file 3 [file DataSheet2.ZIP › ROS Original data/Rosi (3).tif]
