# Supplementary material for: Investigation of anti-diabetic effect of a novel coenzyme Q10 derivative
Source: Front Chem. 2023 Oct 19;11:1280999. doi: 10.3389/fchem.2023.1280999 (PMC10620959; doi:10.3389/fchem.2023.1280999)
Supplement: Supplementary file 2 [file DataSheet1.ZIP › 相关源数据/HRMS.docx]

HRMS (ESI): *m/z* calcd for C_59_H_91_BrNaO_5_ [M+Na]^+^: 981.5948, found:981.5952.
